# Supplementary material for: Comprehensive genome-wide identification of angiosperm upstream ORFs with peptide sequences conserved in various taxonomic ranges using a novel pipeline, ESUCA
Source: BMC Genomics. 2020 Mar 30;21:260. doi: 10.1186/s12864-020-6662-5 (PMC7106846; doi:10.1186/s12864-020-6662-5)
Supplement: Supplementary file 4 — Additional file 4 : Supplementary Table S4. Plasmids used in this study and primers used for plasmid construction. [file 12864_2020_6662_MOESM4_ESM.pdf]

Supplementary Table S4

Supplementary Table S4. Plasmids used in this study and primers used for plasmid construction

Plasmid	Construct	Primer	
		Forward	Reverse
pNH92	*35S::HG46(WT):FLuc*	HG46 for	HG46 rev
pNH93	*35S::HG55(WT):FLuc*	HG55 for	HG55 rev
pNH94	*35S::HG57(WT):FLuc*	HG57 for	HG57 rev
pNH95	*35S::HG65(WT):FLuc*	HG65 for	HG65 rev
pNH96	*35S::HG66(WT):FLuc*	HG66 for	HG66 rev
pNH97	*35S::HG80(WT):FLuc*	HG80 for	HG80 rev
pNH98	*35S::HG81(WT):FLuc*	HG81 for	HG81 rev
pNH99	*35S::HG87(WT):FLuc*	HG87 for	HG87 rev
pNH100	*35S::HG88(WT):FLuc*	HG88 for	HG88 rev
pNH101	*35S::HG103(WT):FLuc*	HG103 for	HG103 rev
pNH102	*35S::HG107(WT):FLuc*	35S XbaI SLiCE-F	FLUC SalI SLiCE-R
pNH103	*35S::HG46(fs):FLuc*	HG46 fs for1	HG46 fs rev1
		HG46 fs for2	HG46 fs rev2
pNH104	*35S::HG55(fs):FLuc*	HG55 fs for1	HG55 fs rev1
pNH105	*35S::HG57(fs):FLuc*	HG57 fs for1	HG57 fs rev1
		HG57 fs for2	HG57 fs rev2
pNH106	*35S::HG063(fs):FLuc*	HG65 fs for1	HG65 fs rev1
		HG65 fs for2	HG65 fs rev2
pNH107	*35S::HG066(fs):FLuc*	HG66 fs for1	HG66 fs rev1
		HG66 fs for2	HG66 fs rev2
pNH108	*35S::HG080(fs):FLuc*	HG80 fsf for1	HG80 fs rev1
		HG80 fs for2	HG80 fs rev2
pNH109	*35S::HG81(fs):FLuc*	HG81 fs for1	HG81 fs rev1
pNH110	*35S::HG87(fs):FLuc*	HG87 fs for1	HG87 fs rev1
		HG87 fs for2	HG87 fs rev2
pNH111	*35S::HG88(fs):FLuc*	HG88 fs for1	HG88 fs rev1
		HG88 fs for2	HG88 fs rev2
pNH113	*35S::HG103(fs):FLuc*	HG103 fs for1	HG103 fs rev1
		HG103 fs for2	HG103 fs rev2
pNH112	*35S::HG107(fs):FLuc*	HG107 fs for	HG107 fs rev
