# Supplementary material for: Comprehensive genome-wide identification of angiosperm upstream ORFs with peptide sequences conserved in various taxonomic ranges using a novel pipeline, ESUCA
Source: BMC Genomics. 2020 Mar 30;21:260. doi: 10.1186/s12864-020-6662-5 (PMC7106846; doi:10.1186/s12864-020-6662-5)
Supplement: Supplementary file 5 — Additional file 5 : Supplementary Table S5. Resourse numbers of Poplus nigra full-length cDNA clones used for cloning of 5′-UTRs. [file 12864_2020_6662_MOESM5_ESM.pdf]

Supplementary Table S5

Supplymentary Table S5. Resourse numbers of *Poplus nigra* full-length cDNA clones used for cloning of 5′-UTRs.

Poplus trichocarpa gene name	Homology Group (HG)	*Poplus nigra* fulle-length cDNA resource number
POPTR_0006s24280	HG46	pds25559
POPTR_0014s10960	HG55	pds10965
POPTR_0006s23570	HG57	pds14390
POPTR_0002s09080	HG65	pds12940
POPTR_0009s15140	HG66	pds13862
POPTR_0009s16830	HG80	pds15817
POPTR_0001s36900	HG81	pds28294
POPTR_0011s04730	HG87	pds26157
POPTR_0004s05670	HG88	pds14623
POPTR_0009s15460	HG103	pds23234
