# Supplementary material for: Comprehensive genome-wide identification of angiosperm upstream ORFs with peptide sequences conserved in various taxonomic ranges using a novel pipeline, ESUCA
Source: BMC Genomics. 2020 Mar 30;21:260. doi: 10.1186/s12864-020-6662-5 (PMC7106846; doi:10.1186/s12864-020-6662-5)
Supplement: Supplementary file 6 — Additional file 6 : Supplementary Table S6. Primers used in this study. [file 12864_2020_6662_MOESM6_ESM.pdf]

Supplementary Table S6

Supplementary Table S6. Primers used in this study

Name	Primer sequence
HG46 for	5´-CTCTCTAGAAGAAGCCAAAAAAAAAAGAAAAGATACA-3´
HG46 rev	5´-TCTGTCGACCTCATTTCCCCAAAATTCCAATTTC-3´
HG55 for	5´-CACGGGGGACTCTAGACTCTTTCTATGTACTATACCTCTCACC-3´
HG55 rev	5´-GTCTTCCATGGTCGACTCCATAACGAGTACCCAAAAGATC-3´
HG57 for	5´-CTCTCTAGAAACAGATACAACACAATCTCCATAC-3´
HG57 rev	5´-TCTGTCGACGCCATTCGTTTCCCTTAAATC-3´
HG65 for	5´-CTCTCTAGAACTGGTATCTCTCTCTCCCTTTTCTA-3´
HG65 rev	5´-TCTGTCGACCCCATCTTAAAACCCTAAAACA-3´
HG66 for	5´-CTCTCTAGAAGCCCAGAATGTCCATCTCCTA-3´
HG66 rev	5´-TCTGTCGACGGCATAATTGTAAGCGACAGTA-3´
HG80 for	5´-CTCTCTAGAGAGATTATAATCAAGGTGGTCAATTGA-3´
HG80 rev	5´-TCTGTCGACTGCATTGAAAATTTGTATCCAACAAC-3´
HG81 for	5´-CTCTCTAGAAAATGTCCTCTTCATTGATTGAGA-3´
HG81 rev	5´-TCTGTCGACCTCATTAAGACTGAGAGAGGGGGA-3´
HG87 for	5´-CACGGGGGACTCTAGAAAGGAAAGGGTGCTGAGTATATCA-3´
HG87 rev	5´-GTCTTCCATGGTCGACCCCATCTCTTAAAACACTACAAGA-3´
HG88 for	5´-CTCTCTAGATGATTAAACAATTTCGAAGACTTTCC-3´
HG88 rev	5´-TCTGTCGACGGCATGATGCAAAGAATGTAGC-3´
HG103 for	5´-CTCTCTAGAGACAACCCTCTCCAAACTC-3´
HG103 rev	5´-TCTGTCGACTCCATCTCTAATAAAAATAAAAATTGG-3´
35S XbaI SLiCE-F	5´-AGAACACGGGGGACTCTAGA-3´
FLUC SalI SLiCE-R	5´-GGCGTCTTCCATGGTCGA-3´
HG46 fs for1	5´-TGTGAACTTTCCCATTCGCTTCTGGGTG-3´
HG46 fs rev1	5´-CACCCAGAAGCGAATGGGAAAGTTCACA-3´
HG46 fs for2	5´-TCAAGCTTCAGAGTGGGATAATTACTATTATCAC-3´
HG46 fs rev2	5´-GTGATAATAGTAATTATCCCACTCTGAAGCTTGA-3´
HG55 fs for1	5´-TGTACTTTTGGGGAAGAAGCGACCGCTGATTAG-3´
HG55 fs rev1	5´-GCTTCTTCCCCAAAAGTACAAAAAAACCAACCCACACACC-3´
HG57 fs for1	5´-GTTTGGAGTTCCTTTGTTCAAGGA-3´
HG57 fs rev1	5´-TCCTTGAACAAAGGAACTCCAAAC-3´
HG57 fs for2	5´-GGAGAAAGAGCAGATAGATATTAGCTCA-3´
HG57 fs rev2	5´-TGAGCTAATATCTATCTGCTCTTTCTCC-3´
HG65 fs for1	5´-TAACACATTAAGATTCGTTACTCCCG-3´
HG65 fs rev1	5´-CGGGAGTAACGAATCTTAATGTGTTA-3´
HG65 fs for2	5´-TTGTTTCTACTTCTTACCCCTGAAA-3´
HG65 fs rev2	5´-TTTCAGGGGTAAGAAGTAGAAACAA-3´
HG66 fs for1	5´-AACGCTCCTCTCTGCTCTCGGTTTC-3´
HG66 fs rev1	5´-GAAACCGAGAGCAGAGAGGAGCGTTG-3´
HG66 fs for2	5´-CTACCGCCGTCTTGAGGTAACC-3´
HG66 fs rev2	5´-GGTTACCTCAAGACGGCGGTAG-3´
HG80 fs for1	5´-CATATTGGGTTCTTCGGATACAAGC-3´
HG80 fs rev1	5´-GCTTGTATCCGAAGAACCCAATATG-3´
HG80 fs for2	5´-TCTTTTGTGTTTCGGTCGTGATAAAAG-3´
HG80 fs rev2	5´-CTTTTATCACGACCGAAACACAAAAGA-3´
HG81 fs for1	5´-CGTGTTTTGTAGCAAAACCGGTTTCTGGTTGAGGATTTA-3´
HG81 fs rev1	5´-CGGTTTTGCTACAAAACACGATCGTTTCAGCCTCA-3´
HG87 fs for1	5´-TCACTTGAGCACATATTTCACTGTTC-3´
HG87 fs rev1	5´-GAACAGTGAAATATGTGCTCAAGTGA-3´
HG87 fs for2	5´-CCAAGGTCCAAGGCTAGCCAGTTA-3´
HG87 fs rev2	5´-TAACTGGCTAGCCTTGGACCTTGG-3´
HG88 fs for1	5´-TAATGGCAATGCATTATATTGCTGTC-3´
HG88 fs rev1	5´-GACAGCAATATAATGCATTGCCATTA-3´
HG88 fs for2	5´-TGCAGAGAGAGACAACTTGCTTTTG-3´
HG88 fs rev2	5´-CAAAAGCAAGTTGTCTCTCTCTGCA-3´

Supplementary Table S6 (continued)

Name	Primer sequence
HG103 fs for1	5´-CAAGAACAACAACACAAGAAAATCCA-3´
HG103 fs rev1	5´-TGGATTTTCTTGTGTTGTTGTTCTTG-3´
HG103 fs for2	5´-GAACCTAACAGACCATAAAAAATCTC-3´
HG103 fs rev2	5´-GAGATTTTTTATGGTCTGTTAGGTTC-3´
HG107 fs for	5´-TCCGGTTCATCCGCTTCTCTCTCTTCTTCTCTGGAGGCCTGATTGA-3´
HG107 fs rev	5´-GAGAAGCGGATGAACCGGAAAGGGTGTAAGTGGA-3´

The recognition sites of restriction enzymes are underlined.
