# Supplementary material for: Comprehensive genome-wide identification of angiosperm upstream ORFs with peptide sequences conserved in various taxonomic ranges using a novel pipeline, ESUCA
Source: BMC Genomics. 2020 Mar 30;21:260. doi: 10.1186/s12864-020-6662-5 (PMC7106846; doi:10.1186/s12864-020-6662-5)
Supplement: Supplementary file 7 — Additional file 7 : Supplementary Figure S1. Alignments of the newly identified CPuORF sequences. [file 12864_2020_6662_MOESM7_ESM.pdf]

Supplementary Figure S1

HG002.2

POPTR_0001s22450

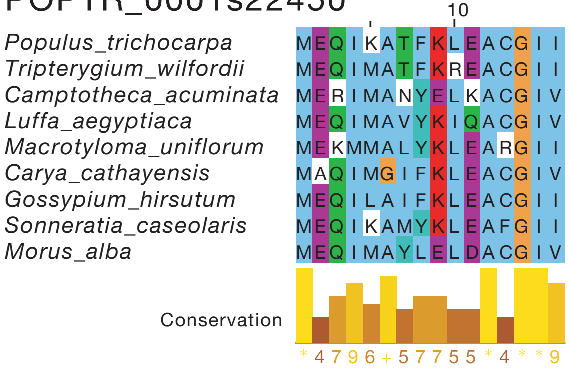

HG009.2

POPTR_0003s01750

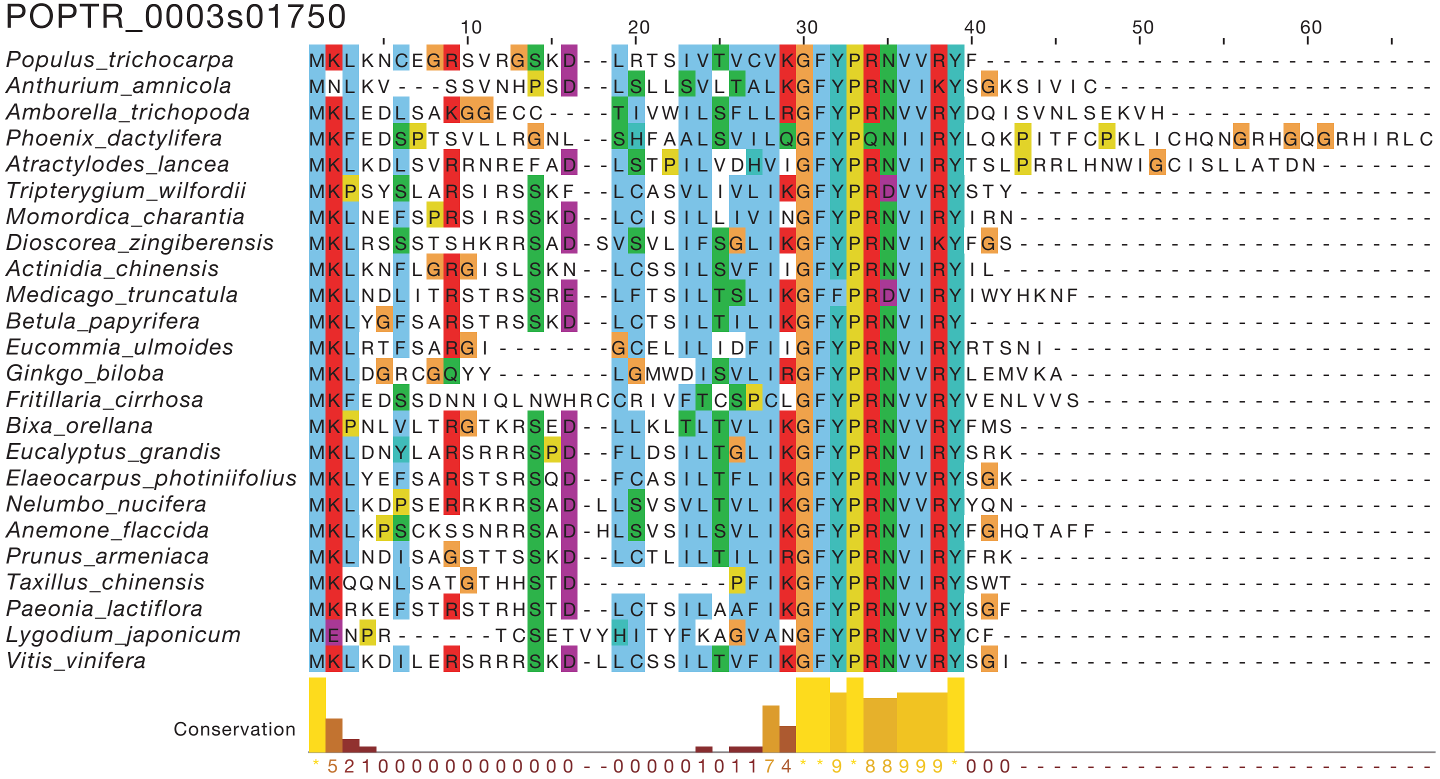

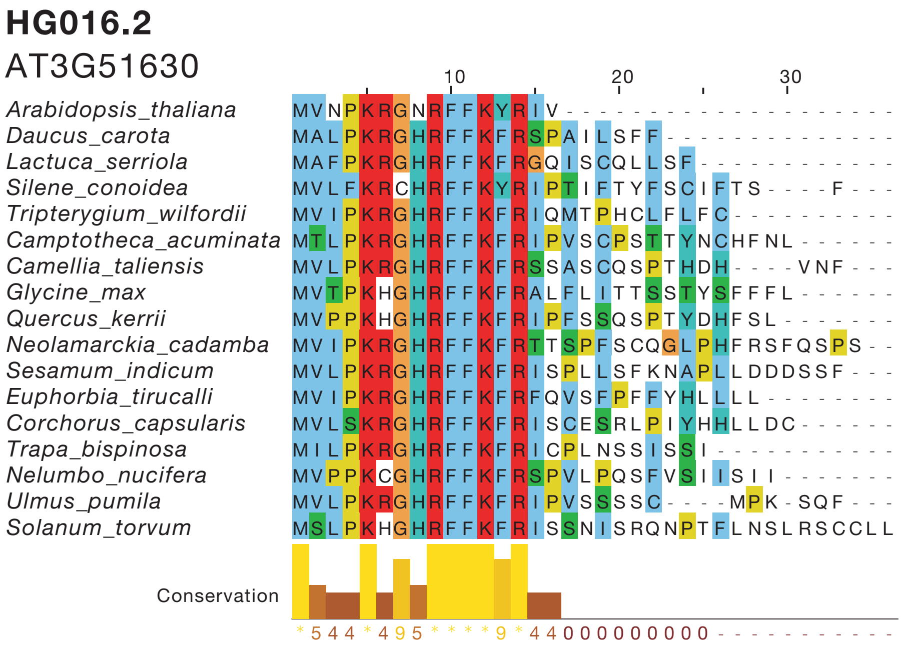

HG016.2
AT3G51630
10
20
30
Arabidopsis_thaliana
Daucus_carota
Lactuca_serriola
Silene_conoidea
Tripterygium_wilfordii
Camptotheca_acuminata
Camellia_taliensis
Glycine_max
Quercus_kerrii
Neolamarckia_cadamba
Sesamum_indicum
Euphorbia_tirucalli
Corchorus_capsularis
Trapa_bispinosa
Nelumbo_nucifera
Ulmus_pumila
Solanum_torvum
Conservation

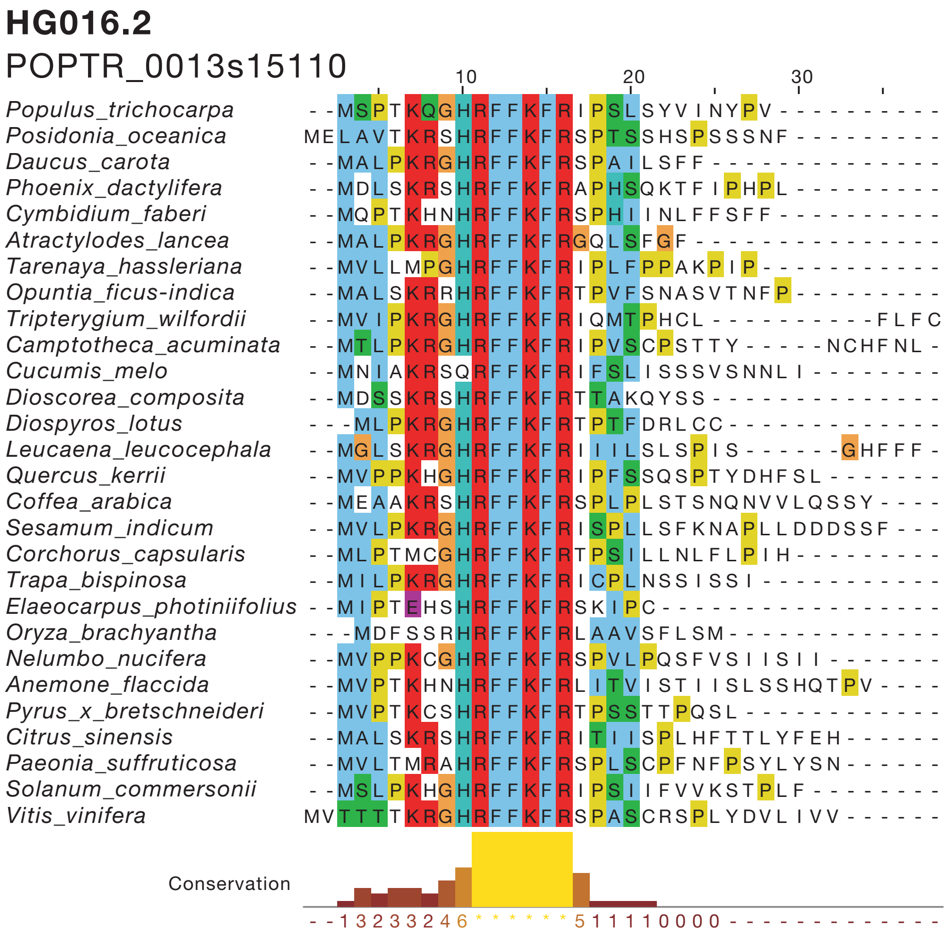

HG016.2
POPTR_0013s15110
10
20
30
Populus_trichocarpa
Posidonia_oceanica
Daucus_carota
Phoenix_dactylifera
Cymbidium_faberi
Atractylodes_lancea
Tarenaya_hassleriana
Opuntia_ficus-indica
Tripterygium_wilfordii
Camptotheca_acuminata
Cucumis_melo
Dioscorea_composita
Diospyros_lotus
Leucaena_leucocephala
Quercus_kerrii
Coffea_arabica
Sesamum_indicum
Corchorus_capsularis
Trapa_bispinosa
Elaeocarpus_photiniifolius
Oryza_brachyantha
Nelumbo_nucifera
Anemone_flaccida
Pyrus_x_bretschneideri
Citrus_sinensis
Paeonia_suffruticosa
Solanum_commersonii
Vitis_vinifera
Conservation

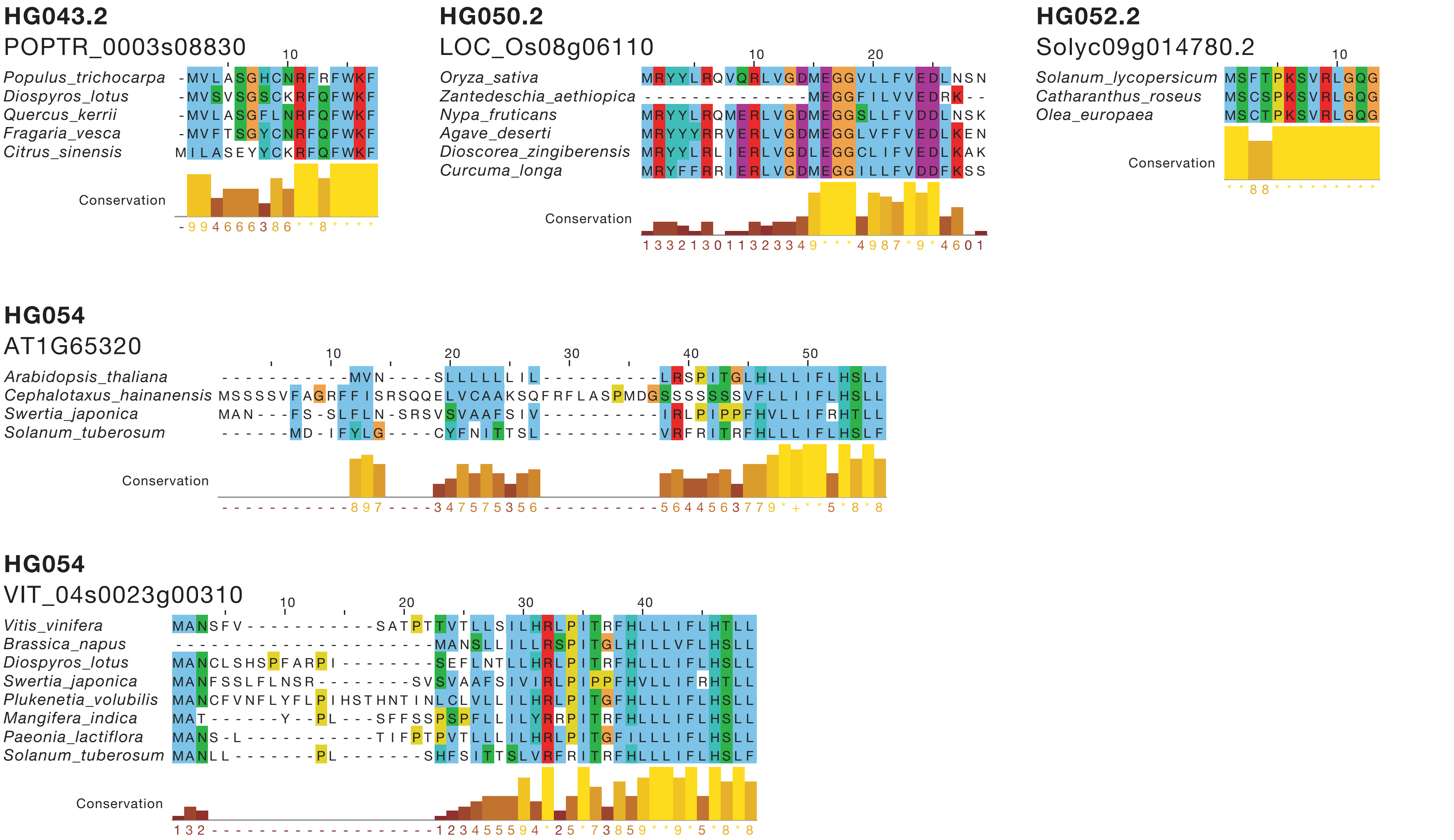
HG043.2
POPTR_0003s08830
Populus_trichocarpa -MVLASGHCNRFRFWKF
Diospyros_lotus -MVSVSGSCKRFQFWKF
Quercus_kerrii -MVLASGFLNRFQFWKF
Fragaria_vesca -MVFTSGYCNRFQFWKF
Citrus_sinensis MILASEYYCKRFQFWKF
Conservation
-9946663386**8****
HG050.2
LOC_Os08g06110
Oryza_sativa MRYYLRQVQRLVGDMEGGVLLFVEDLNSN
Zantedeschia_aethiopica --------------MEGGFILVVEDRK--
Nypa_fruticans MRYYLRQMERLVGDMEGGSLLFVDDLNSK
Agave_deserti MRYYYRRVERLVGDMEGGLVFFVEDLKEN
Dioscorea_zingiberensis MRYYLRLIERLVGDLEGGCLIFVEDLKAK
Curcuma_longa MRYFFRRIERLVGDMEGGILLFVDDFKSS
Conservation
1332130113233349***4987*9*4601
HG052.2
Solyc09g014780.2
Solanum_lycopersicum MSFTPKSVRLGQG
Catharanthus_roseus MSCSPKSVRLGQG
Olea_europaea MSCTPKSVRLGQG
Conservation
88*******
HG054
AT1G65320
Arabidopsis_thaliana -----------MVN----SLLLLLIL----------LRSPITGLHLLIFLHSLL
Cephalotaxus_hainanensis MSSSSVFAGRFFISRSQQELVCAAKSQFRFLASPMDGSSSSSSVFLLIIFLHSLL
Swertia_japonica MAN---FS-SLFLN-SRSVSVAAFSIV----------IRLPIPPFHVLLIFRHTLL
Solanum_tuberosum ------MD-IFYLG----CYFNITTSL----------VRFRITRFHLLLIFLHSLF
Conservation
-----------897----347575356----------5644563779*+**5*8*8
HG054
VIT_04s0023g00310
Vitis_vinifera MANSFV-----------SATPTTVTLLSILHRLPITRFHLLLIFLHTLL
Brassica_napus ----------------------MANSLLILLRSPITGLHILLVFLHSLL
Diospyros_lotus MANCLSHSPFARPI--------SEFLNTLLHRLPITRFHLLLIFLHSLL
Swertia_japonica MANFSSLFLNSR--------SVSVAAFSIVIRLPIPPFHVLLIFRHTLL
Plukenetia_volubilis MANCFVNFLYFLPIHSTHNTINLCLVLLILHRLPITGFHLLLIFLHSLL
Mangifera_indica MAT------Y--PL---SFFSSPSPFLLILYRRPITRFHLLLIFLHSLL
Paeonia_lactiflora MANS-L-----------TIFPTPVTLLLILHRLPITGFILLLIFLHSLL
Solanum_tuberosum MANLL-------PL--------SHFSITTSLVRFRITRFHLLLIFLHSLF
Conservation
132-------------------1234555594*25*73859**9*5*8*8

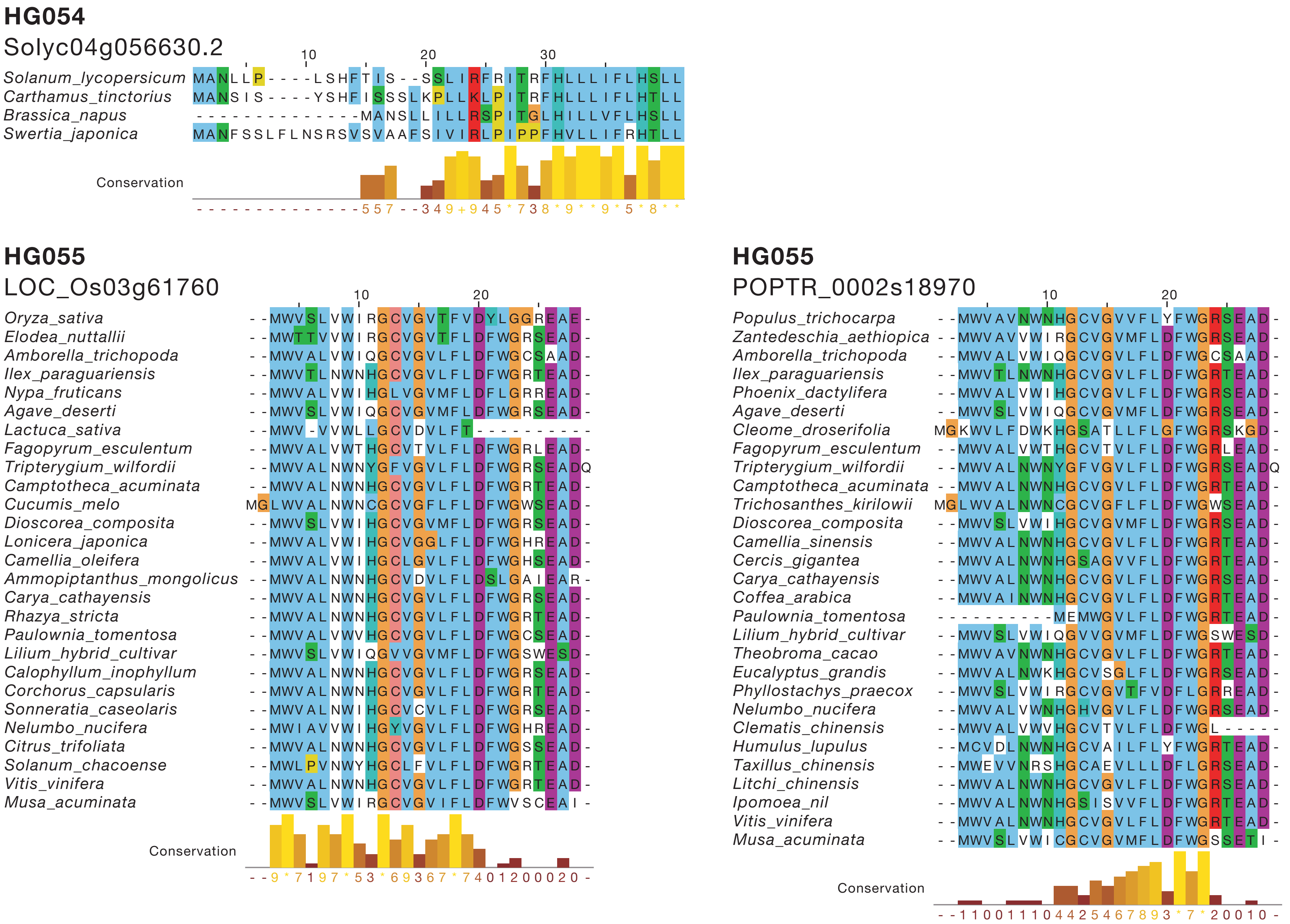
HG054
Solyc04g056630.2
Solanum_lycopersicum
Carthamus_tinctorius
Brassica_napus
Swertia_japonica
Conservation
HG055
LOC_Os03g61760
Oryza_sativa
Elodea_nuttallii
Amborella_trichopoda
Ilex_paraguariensis
Nypa_fruticans
Agave_deserti
Lactuca_sativa
Fagopyrum_esculentum
Tripterygium_wilfordii
Camptotheca_acuminata
Cucumis_melo
Dioscorea_composita
Lonicera_japonica
Camellia_oleifera
Ammopiptanthus_mongolicus
Carya_cathayensis
Rhazya_stricta
Paulownia_tomentosa
Lilium_hybrid_cultivar
Calophyllum_inophyllum
Corchorus_capsularis
Sonneratia_caseolaris
Nelumbo_nucifera
Citrus_trifoliata
Solanum_chacoense
Vitis_vinifera
Musa_acuminata
Conservation
HG055
POPTR_0002s18970
Populus_trichocarpa
Zantedeschia_aethiopica
Amborella_trichopoda
Ilex_paraguariensis
Phoenix_dactylifera
Agave_deserti
Cleome_droserifolia
Fagopyrum_esculentum
Tripterygium_wilfordii
Camptotheca_acuminata
Trichosanthes_kirilowii
Dioscorea_composita
Camellia_sinensis
Cercis_gigantea
Carya_cathayensis
Coffea_arabica
Paulownia_tomentosa
Lilium_hybrid_cultivar
Theobroma_cacao
Eucalyptus_grandis
Phyllostachys_praecox
Nelumbo_nucifera
Clematis_chinensis
Humulus_lupulus
Taxillus_chinensis
Litchi_chinensis
Ipomoea_nil
Vitis_vinifera
Musa_acuminata
Conservation

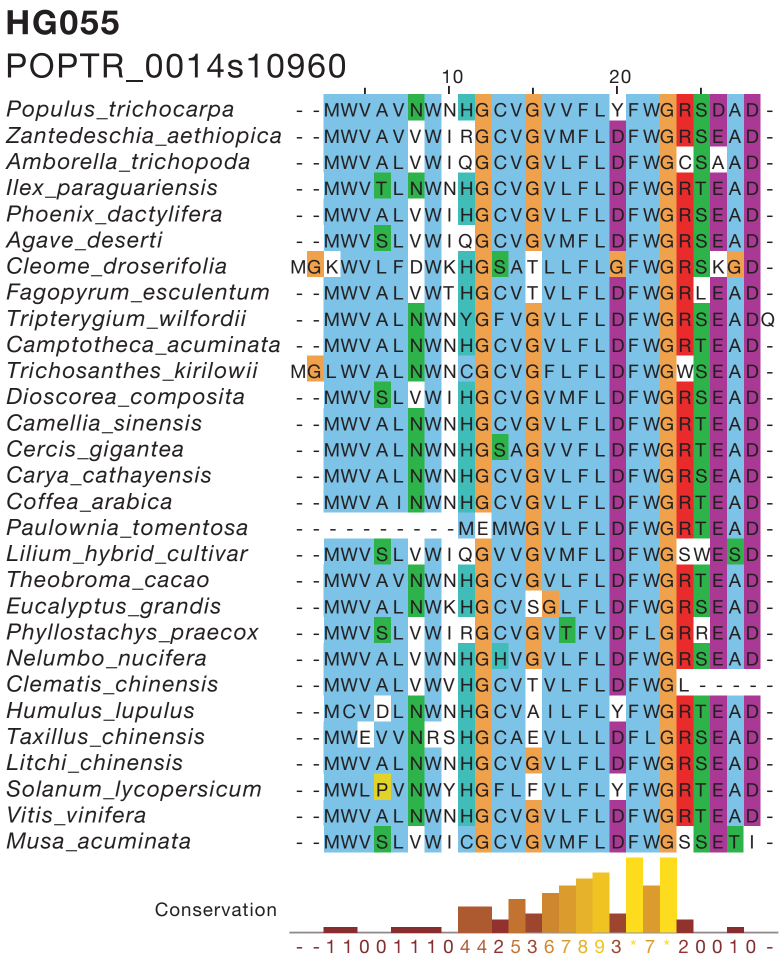
HG055
POPTR_0014s10960
Populus_trichocarpa
Zantedeschia_aethiopica
Amborella_trichopoda
Ilex_paraguariensis
Phoenix_dactylifera
Agave_deserti
Cleome_droserifolia
Fagopyrum_esculentum
Tripterygium_wilfordii
Camptotheca_acuminata
Trichosanthes_kirilowii
Dioscorea_composita
Camellia_sinensis
Cercis_gigantea
Carya_cathayensis
Coffea_arabica
Paulownia_tomentosa
Lilium_hybrid_cultivar
Theobroma_cacao
Eucalyptus_grandis
Phyllostachys_praecox
Nelumbo_nucifera
Clematis_chinensis
Humulus_lupulus
Taxillus_chinensis
Litchi_chinensis
Solanum_lycopersicum
Vitis_vinifera
Musa_acuminata
Conservation

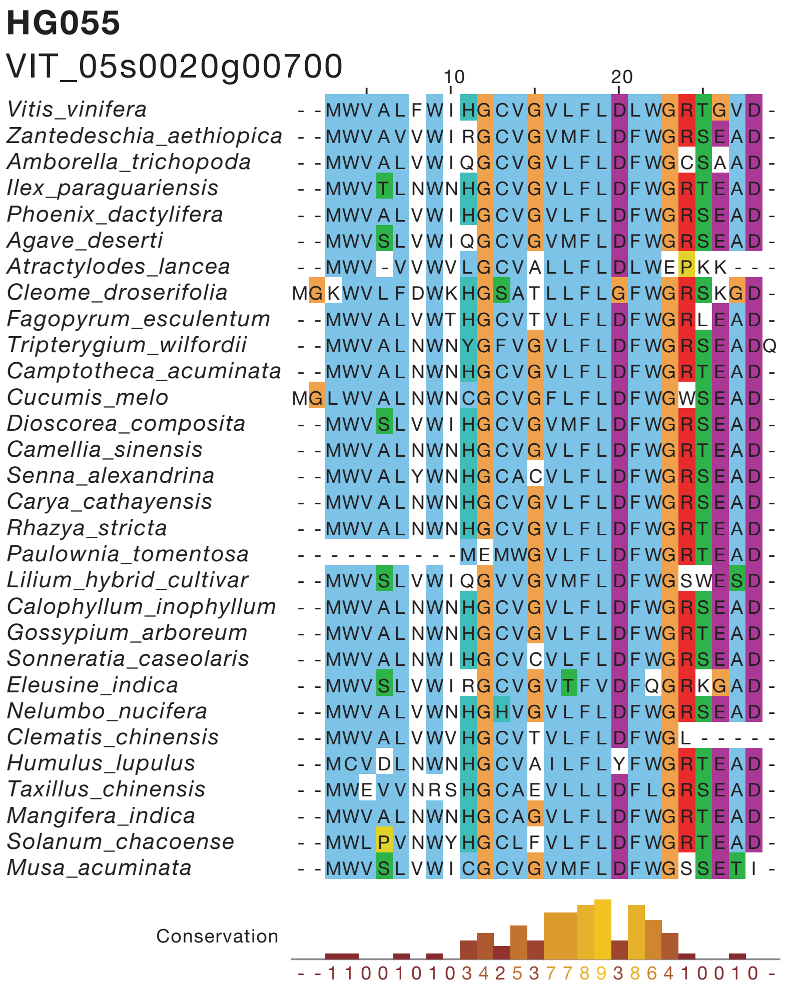
HG055
VIT_05s0020g00700
Vitis_vinifera
Zantedeschia_aethiopica
Amborella_trichopoda
Ilex_paraguariensis
Phoenix_dactylifera
Agave_deserti
Atractylodes_lancea
Cleome_droserifolia
Fagopyrum_esculentum
Tripterygium_wilfordii
Camptotheca_acuminata
Cucumis_melo
Dioscorea_composita
Camellia_sinensis
Senna_alexandrina
Carya_cathayensis
Rhazya_stricta
Paulownia_tomentosa
Lilium_hybrid_cultivar
Calophyllum_inophyllum
Gossypium_arboreum
Sonneratia_caseolaris
Eleusine_indica
Nelumbo_nucifera
Clematis_chinensis
Humulus_lupulus
Taxillus_chinensis
Mangifera_indica
Solanum_chacoense
Musa_acuminata
Conservation

HG055

VIT_07s0005g02260

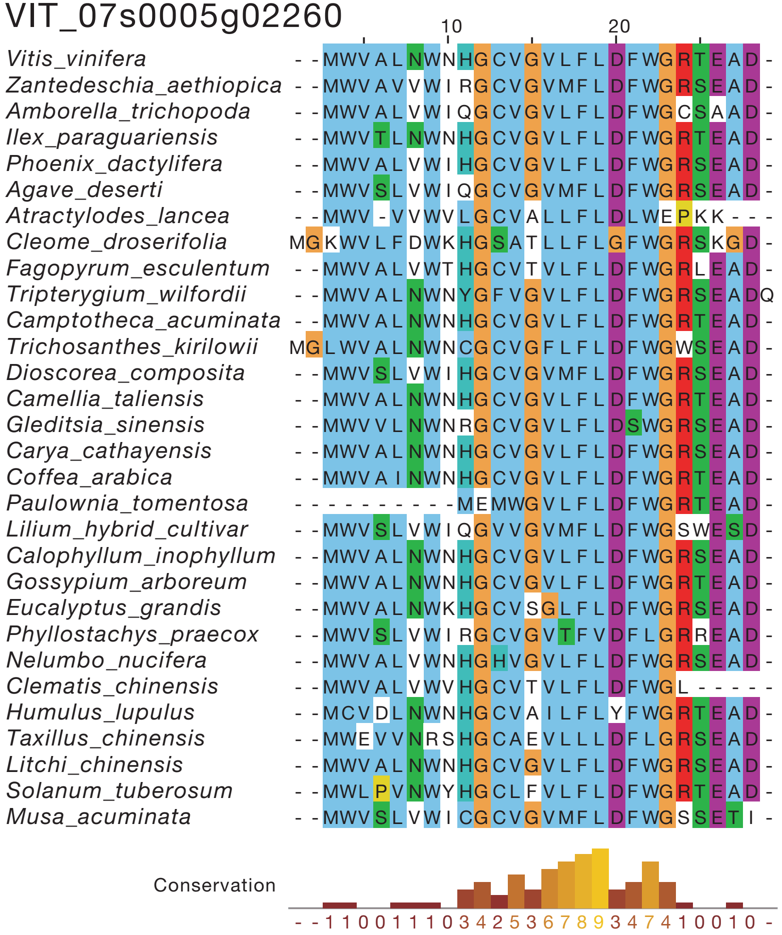

HG056.1

POPTR_0004s05490

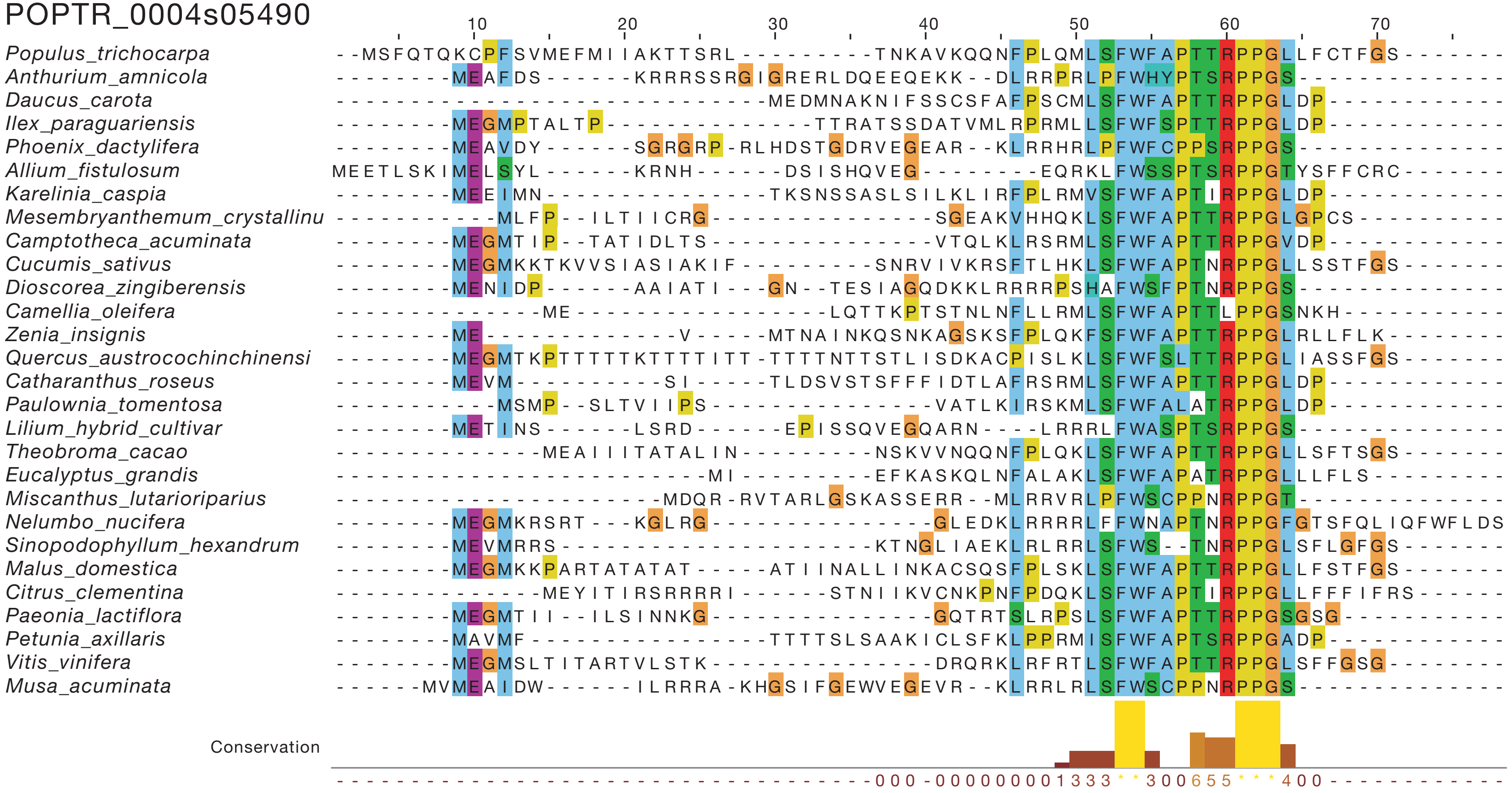

HG056.1

Solyc02g076920.2

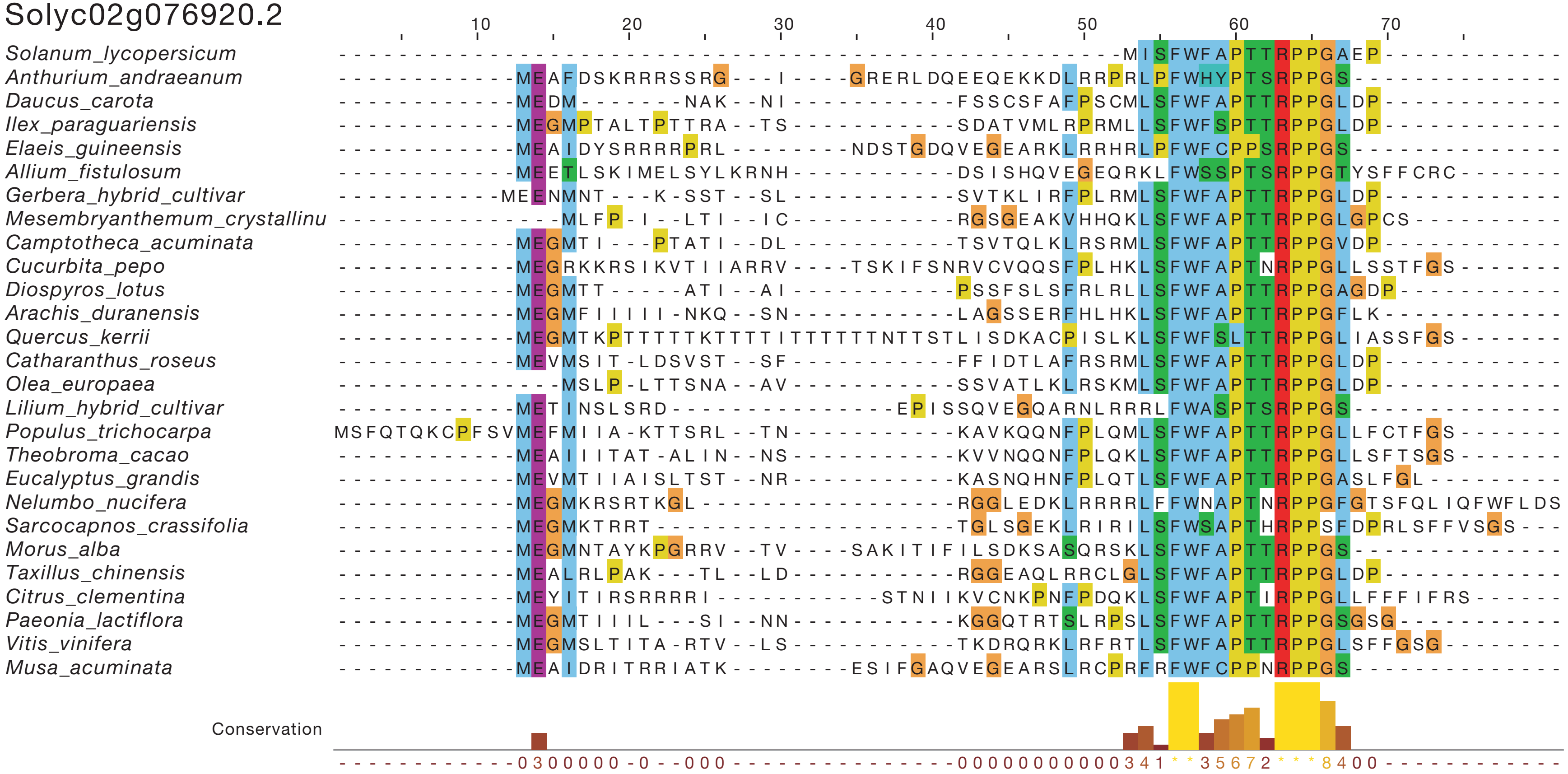

HG056.1

Solyc07g053290.2

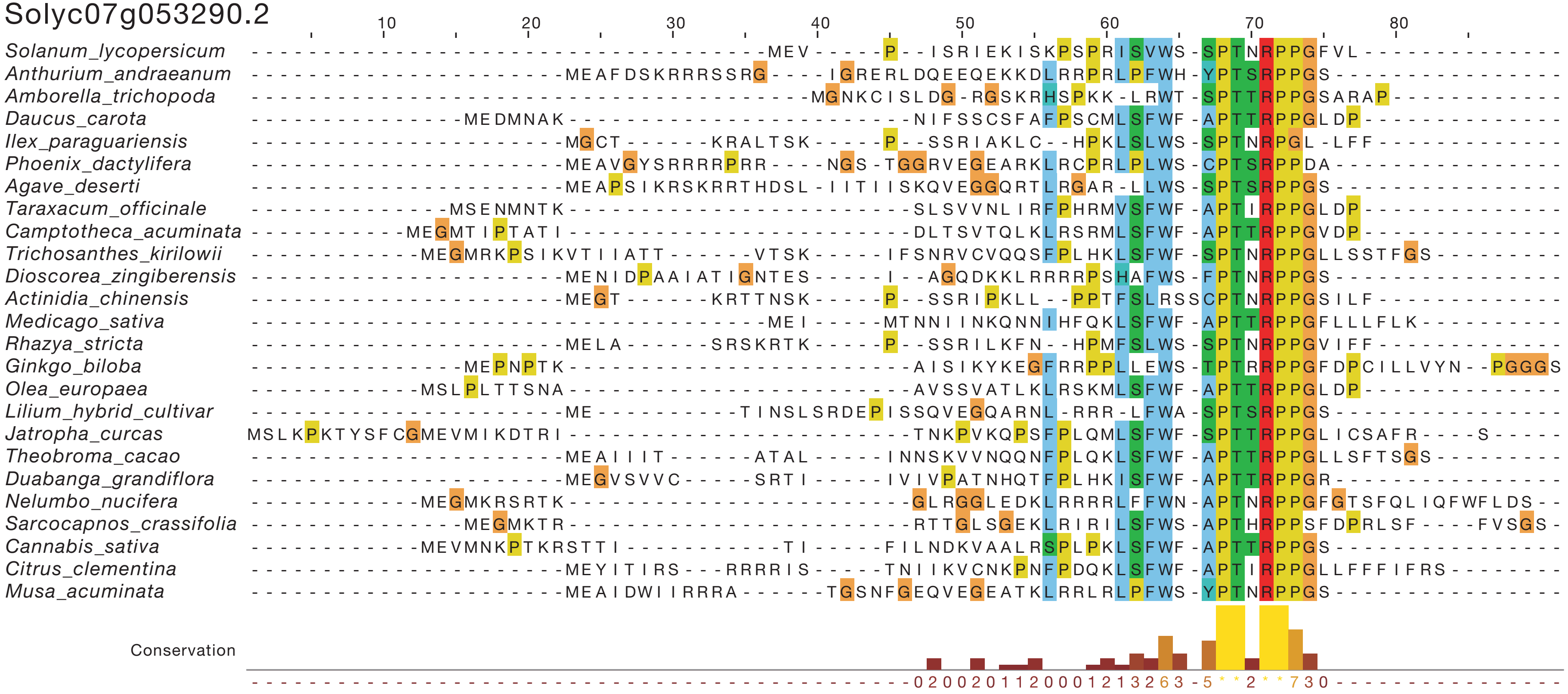

HG056.1

VIT_10s0003g01170

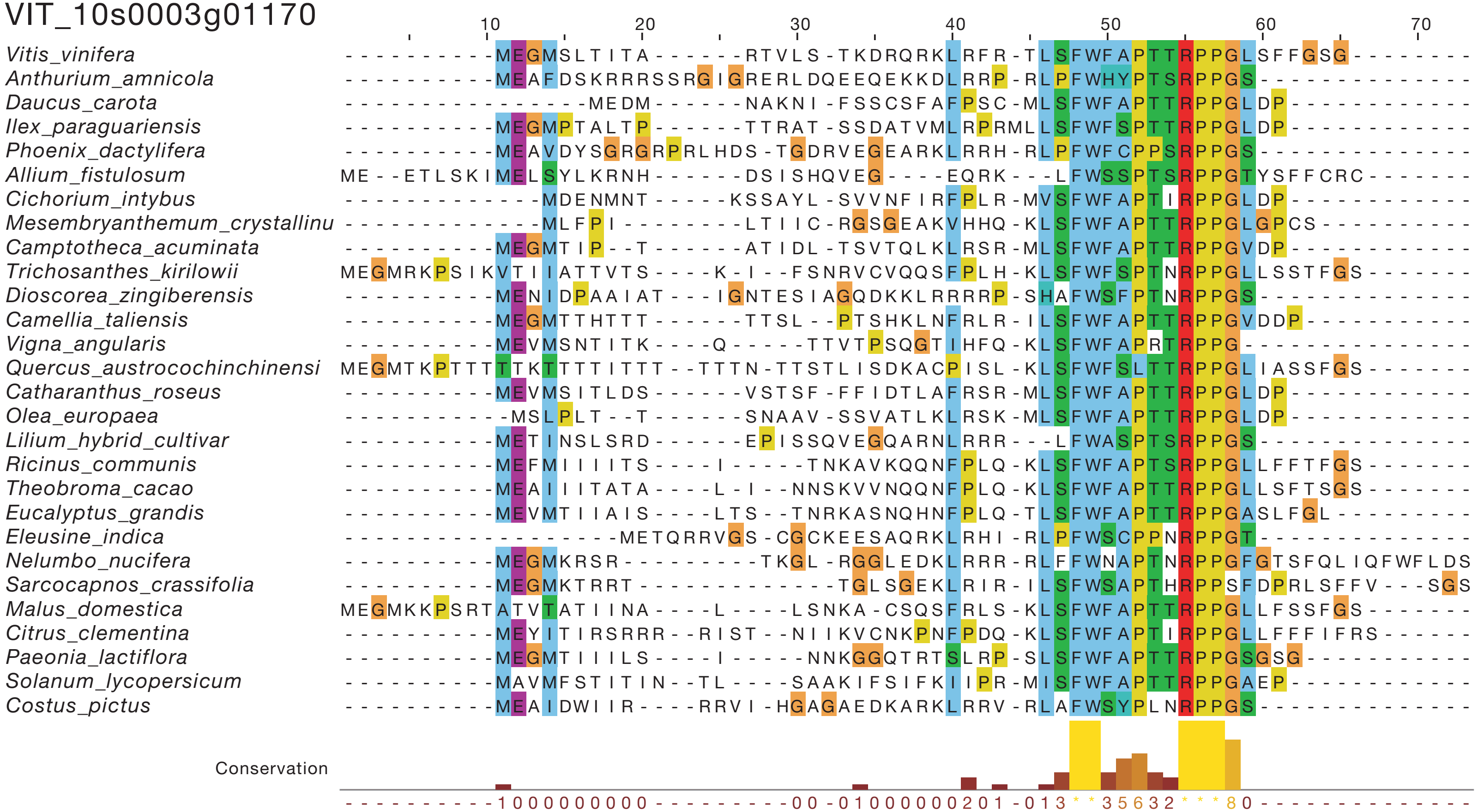

HG056.2
POPTR_0004s05490

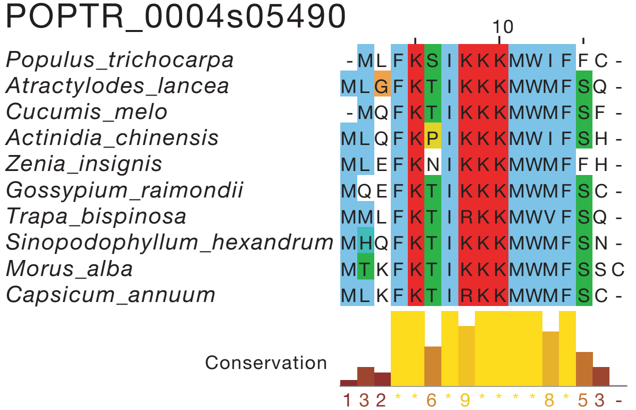

HG056.2
VIT_10s0003g01170

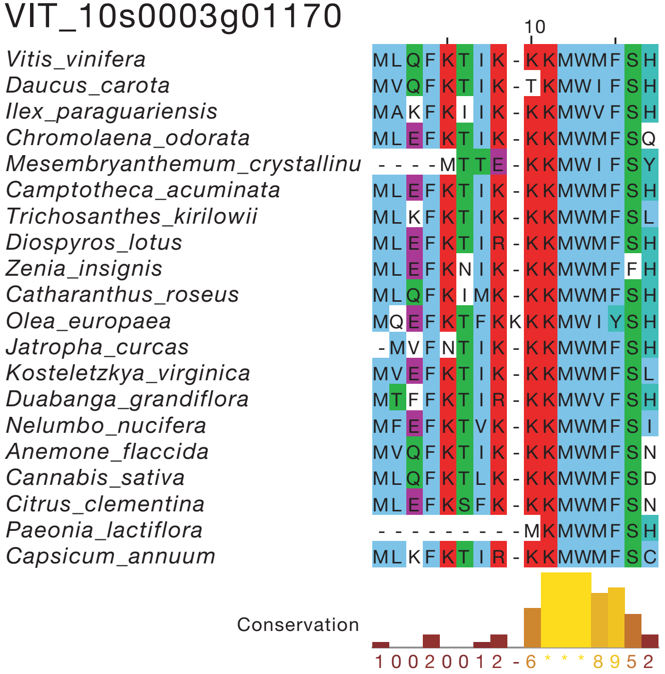

HG057
AT1G54095

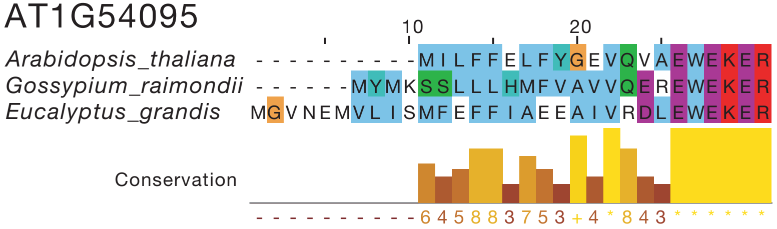

HG057
AT1G72510

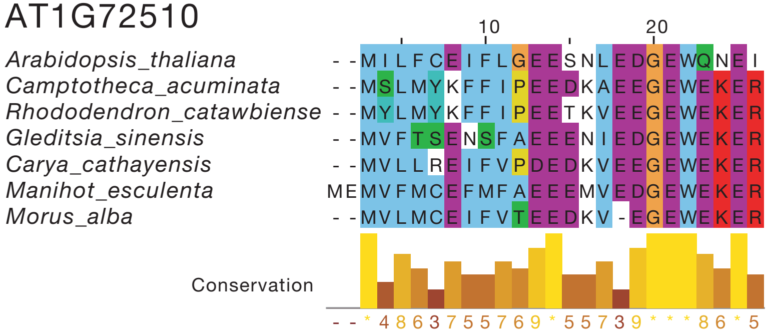

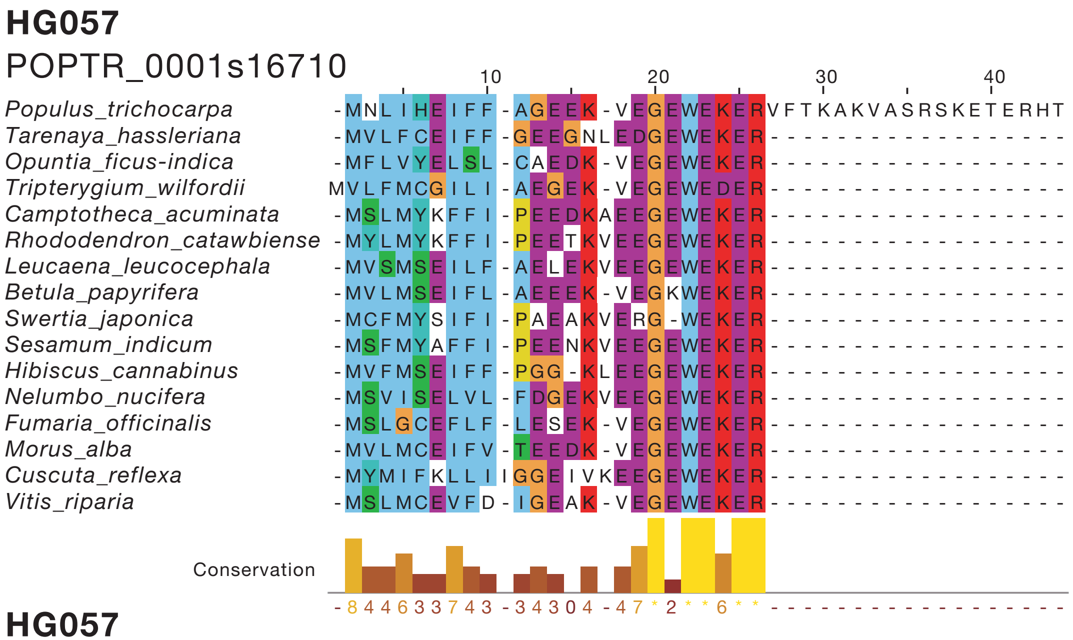
HG057
POPTR_0001s16710
Populus_trichocarpa
Tarenaya_hassleriana
Opuntia_ficus-indica
Tripterygium_wilfordii
Camptotheca_acuminata
Rhododendron_catawbiense
Leucaena_leucocephala
Betula_papyrifera
Swertia_japonica
Sesamum_indicum
Hibiscus_cannabinus
Nelumbo_nucifera
Fumaria_officinalis
Morus_alba
Cuscuta_reflexa
Vitis_riparia
Conservation

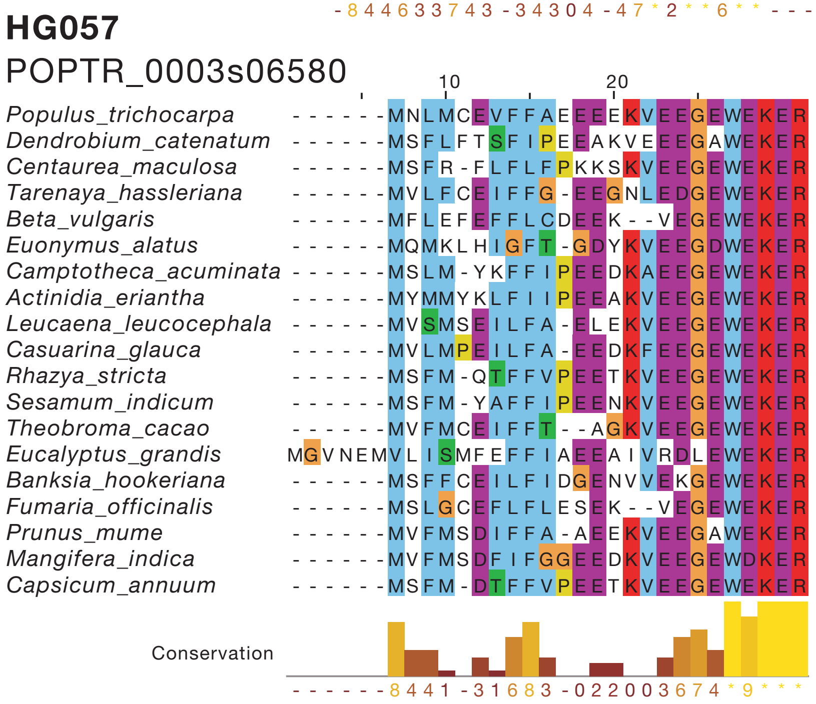
HG057
POPTR_0003s06580
Populus_trichocarpa
Dendrobium_catenatum
Centaurea_maculosa
Tarenaya_hassleriana
Beta_vulgaris
Euonymus_alatus
Camptotheca_acuminata
Actinidia_eriantha
Leucaena_leucocephala
Casuarina_glauca
Rhazya_stricta
Sesamum_indicum
Theobroma_cacao
Eucalyptus_grandis
Banksia_hookeriana
Fumaria_officinalis
Prunus_mume
Mangifera_indica
Capsicum_annuum
Conservation

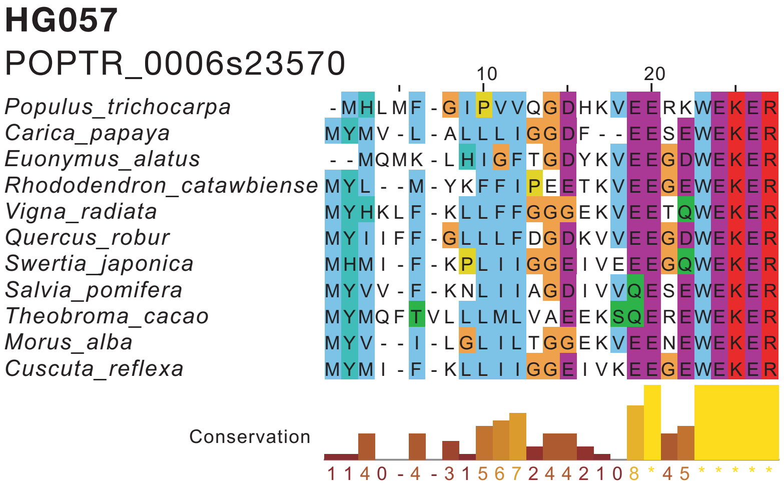
HG057
POPTR_0006s23570
Populus_trichocarpa
Carica_papaya
Euonymus_alatus
Rhododendron_catawbiense
Vigna_radiata
Quercus_robur
Swertia_japonica
Salvia_pomifera
Theobroma_cacao
Morus_alba
Cuscuta_reflexa
Conservation

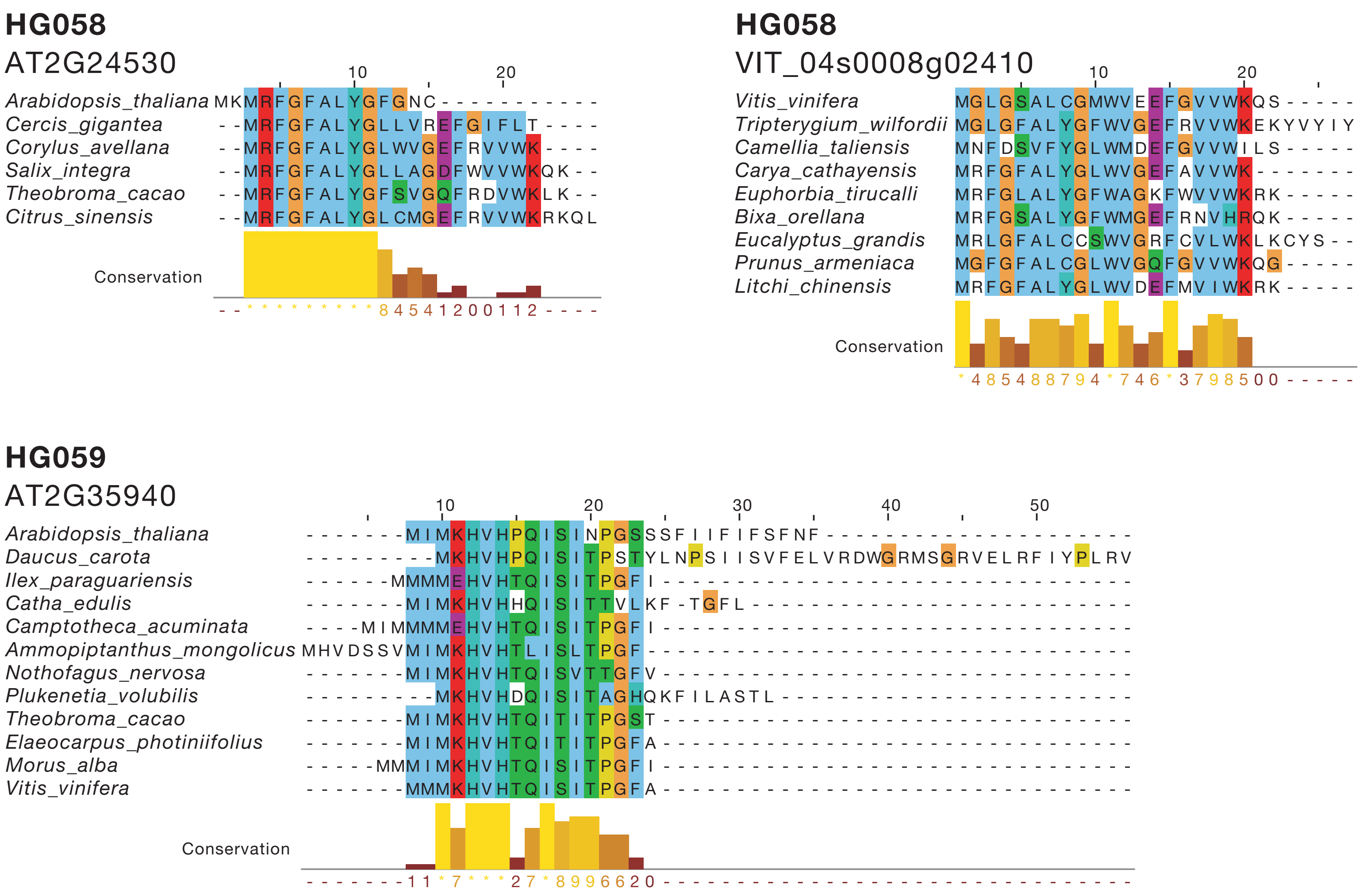
HG058
AT2G24530
10
20
Arabidopsis_thaliana MKMRFGFALYGFGNC-----------
Cercis_gigantea --MRFGFALYGLLVREFGIFLT----
Corylus_avellana --MRFGFALYGLWVGEFRVVWK----
Salix_integra --MRFGFALYGLLAGDFWVVWKQK--
Theobroma_cacao --MRFGFALYGFSVGQFRDVWKLK--
Citrus_sinensis --MRFGFALYGLCMGEFRVVWKRKQL
Conservation
--*********845412001 12----
HG058
VIT_04s0008g02410
10
20
Vitis_vinifera MGLGSALCGMWVEEFGVVWKQS-----
Tripterygium_wilfordii MGLGFALYGFWVGEFRVVWKEKYVYIY
Camellia_taliensis MNFDSVFYGLWMDEFGVVWILS-----
Carya_cathayensis MRFGFALYGLWVGEFAVVWK-------
Euphorbia_tirucalli MRFGLALYGFWAGKFWVVWKRK-----
Bixa_orellana MRFGSALYGFWMGEFRNVHRQK-----
Eucalyptus_grandis MRLGFALCCSWVGRFCVLWKLKCYS--
Prunus_armeniaca MGFGFALCGLWVGQFGVVWKQG-----
Litchi_chinensis MRFGFALYGLWVDEFMVIWKRK-----
Conservation
*4854887 94*746*379850 0-----
HG059
AT2G35940
10
20
30
40
50
Arabidopsis_thaliana -------MIMKHVHPQISINPGSSSFIIFIFSFNF---------------------
Daucus_carota ---------MKHVHPQISITPSTYLNPSIISVFELVRDWGRMSGRVELRFIYPLRV
Ilex_paraguariensis ------MMMMEHVHTQISITPGFI--------------------------------
Catha_edulis -------MIMKHVHHQISITTVLKF-TGFL--------------------------
Camptotheca_acuminata ----MIMMMMEHVHTQISITPGFI--------------------------------
Ammopiptanthus_mongolicus MHVDSSVMIMKHVHTLISLTPGF---------------------------------
Nothofagus_nervosa -------MIMKHVHTQISVTTGFV--------------------------------
Plukenetia_volubilis ---------MKHVHDQISITAGHQKFILASTL------------------------
Theobroma_cacao -------MIMKHVHTQITITPGST--------------------------------
Elaeocarpus_photiniifolius -------MIMKHVHTQITITPGFA--------------------------------
Morus_alba -----MMMIMKHVHTQISITPGFI--------------------------------
Vitis_vinifera -------MMMKHVHTQISITPGFA--------------------------------
Conservation
-------11*7***27*899662 0--------------------------------

HG059

POPTR_0006s21950

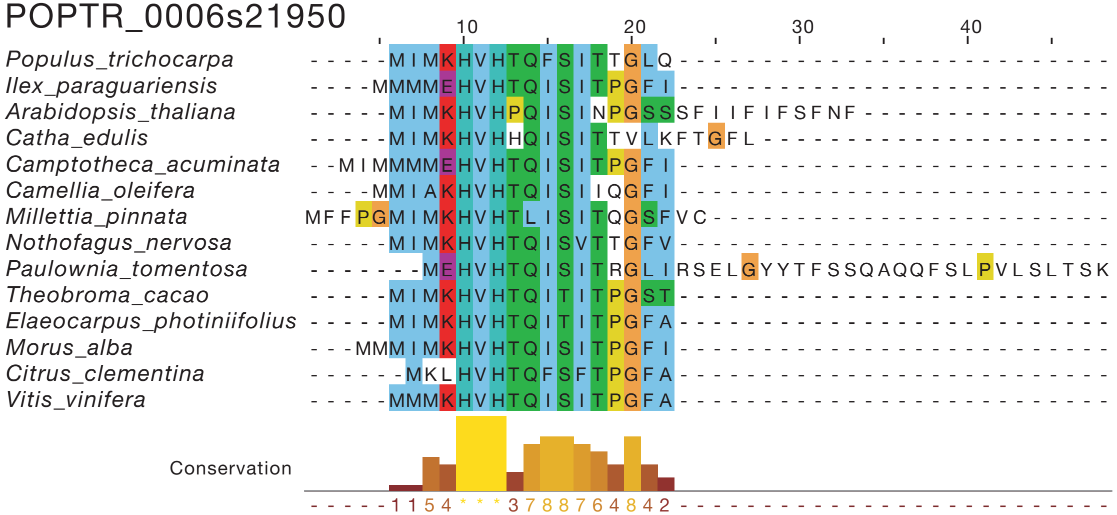

HG059

POPTR_0016s07040

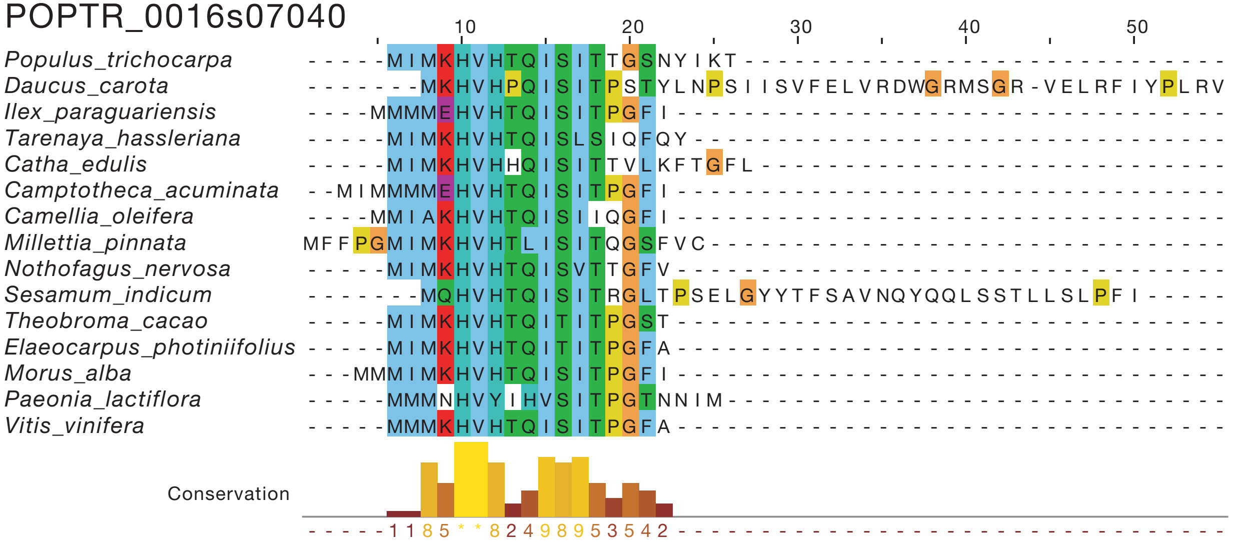

HG060
AT3G12570

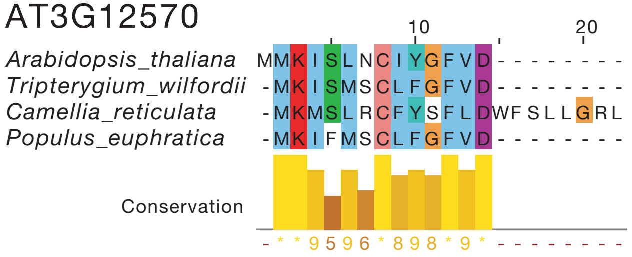

HG060
POPTR_0008s05420

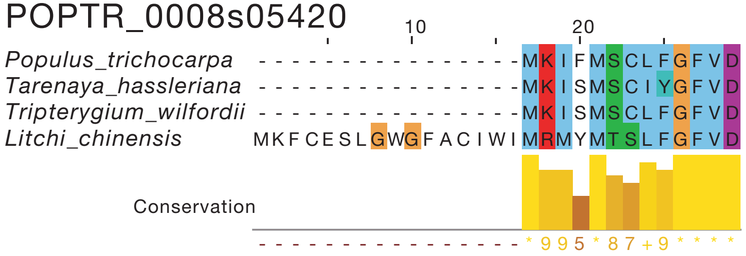

HG060
POPTR_0010s21340

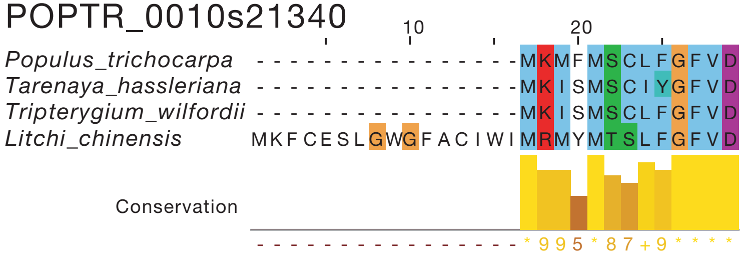

HG061

AT5G55600

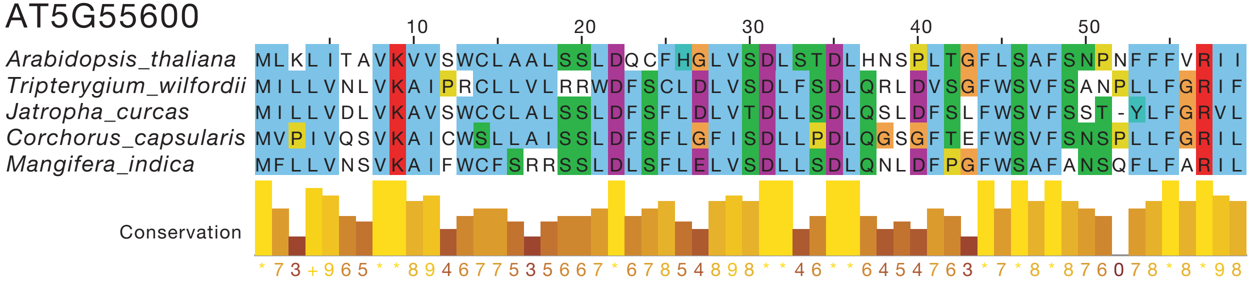

HG061

POPTR_0001s37450

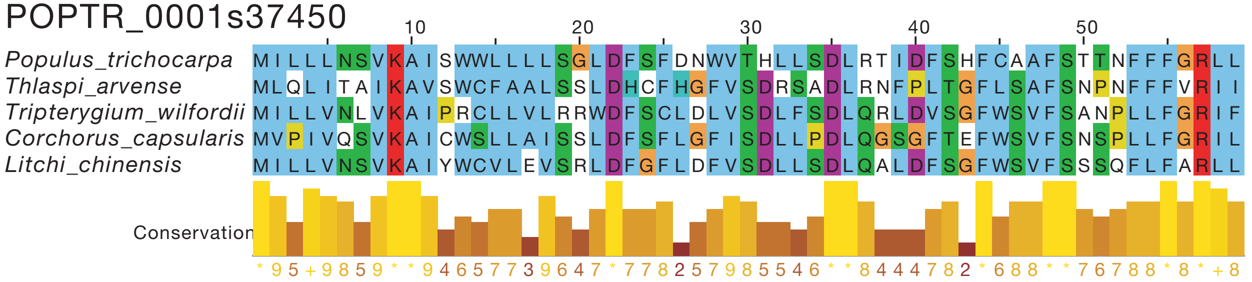

HG062

POPTR_0008s09440

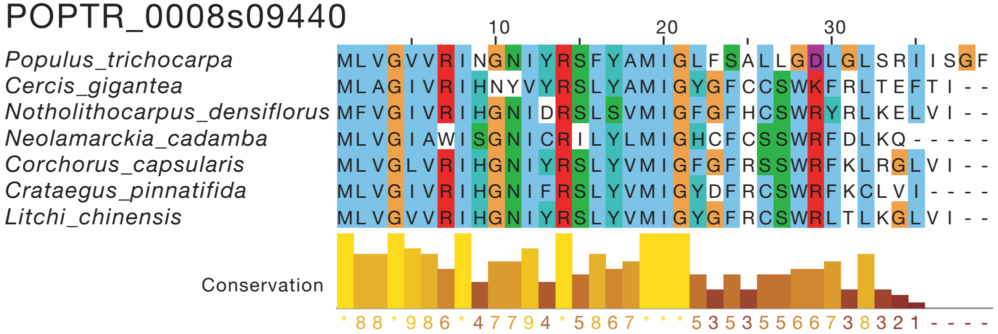

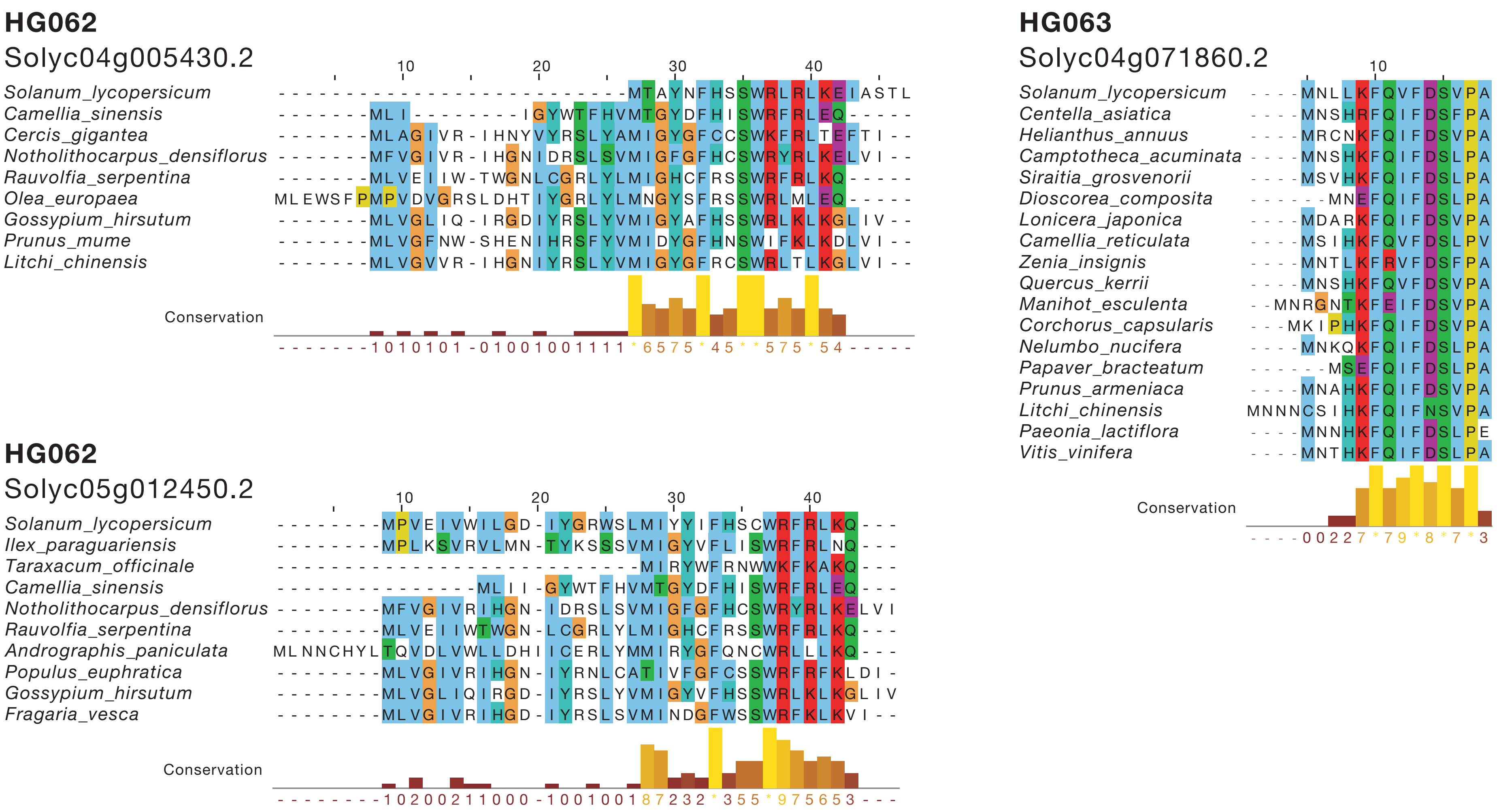
HG062
Solyc04g005430.2
Solanum_lycopersicum
Camellia_sinensis
Cercis_gigantea
Notholithocarpus_densiflorus
Rauvolfia_serpentina
Olea_europaea
Gossypium_hirsutum
Prunus_mume
Litchi_chinensis
Conservation
HG062
Solyc05g012450.2
Solanum_lycopersicum
Ilex_paraguariensis
Taraxacum_officinale
Camellia_sinensis
Notholithocarpus_densiflorus
Rauvolfia_serpentina
Andrographis_paniculata
Populus_euphratica
Gossypium_hirsutum
Fragaria_vesca
Conservation
HG063
Solyc04g071860.2
Solanum_lycopersicum
Centella_asiatica
Helianthus_annuus
Camptotheca_acuminata
Siraitia_grosvenorii
Dioscorea_composita
Lonicera_japonica
Camellia_reticulata
Zenia_insignis
Quercus_kerrii
Manihot_esculenta
Corchorus_capsularis
Nelumbo_nucifera
Papaver_bracteatum
Prunus_armeniaca
Litchi_chinensis
Paeonia_lactiflora
Vitis_vinifera
Conservation

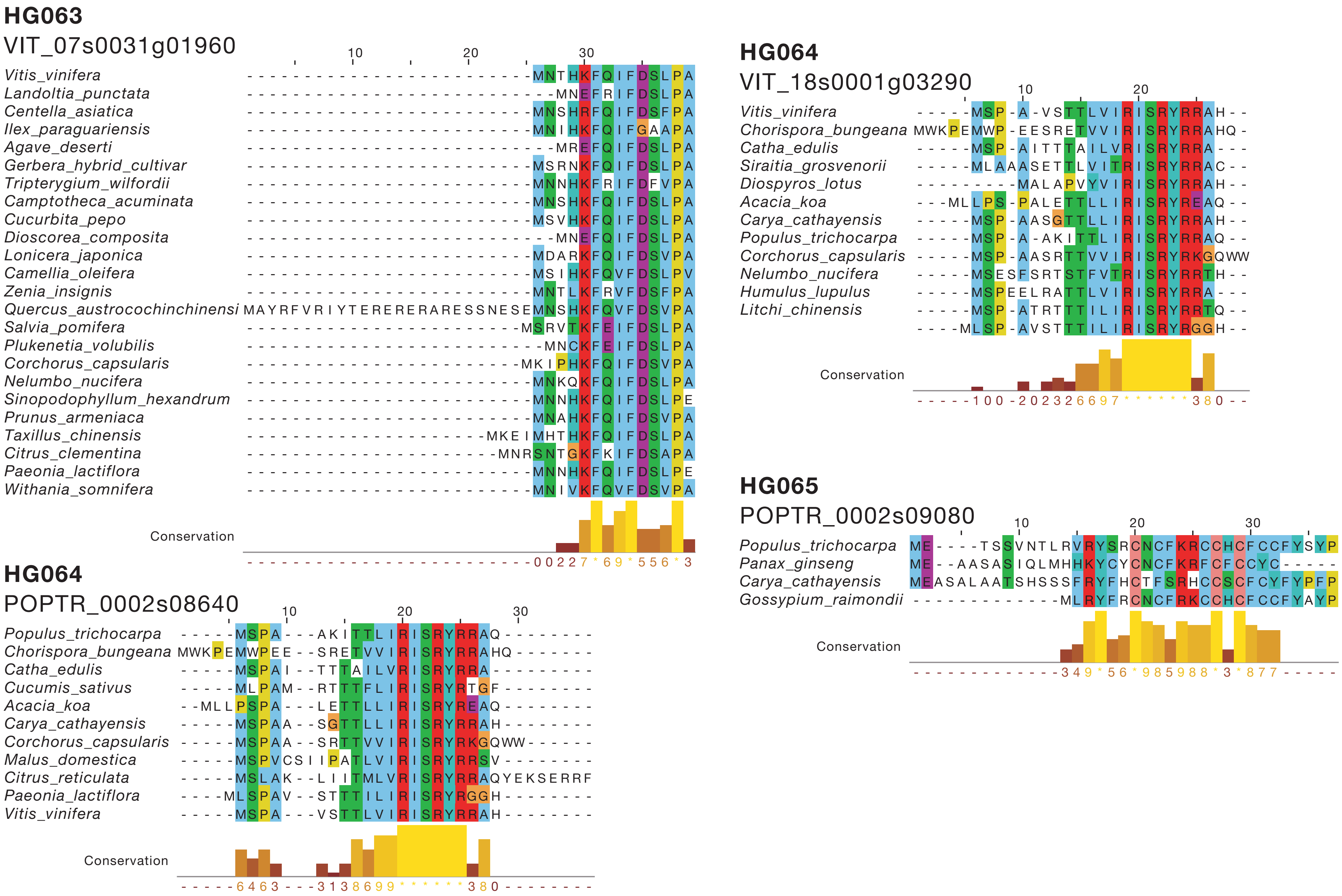
HG063
VIT_07s0031g01960
10
20
30
Vitis_vinifera
Landoltia_punctata
Centella_asiatica
Ilex_paraguariensis
Agave_deserti
Gerbera_hybrid_cultivar
Tripterygium_wilfordii
Camptotheca_acuminata
Cucurbita_pepo
Dioscorea_composita
Lonicera_japonica
Camellia_oleifera
Zenia_insignis
Quercus_austrocochinchinensi
Salvia_pomifera
Plukenetia_volubilis
Corchorus_capsularis
Nelumbo_nucifera
Sinopodophyllum_hexandrum
Prunus_armeniaca
Taxillus_chinensis
Citrus_clementina
Paeonia_lactiflora
Withania_somnifera
Conservation
HG064
POPTR_0002s08640
Populus_trichocarpa
Chorispora_bungeana
Catha_edulis
Cucumis_sativus
Acacia_koa
Carya_cathayensis
Corchorus_capsularis
Malus_domestica
Citrus_reticulata
Paeonia_lactiflora
Vitis_vinifera
Conservation
HG064
VIT_18s0001g03290
Vitis_vinifera
Chorispora_bungeana
Catha_edulis
Siraitia_grosvenorii
Diospyros_lotus
Acacia_koa
Carya_cathayensis
Populus_trichocarpa
Corchorus_capsularis
Nelumbo_nucifera
Humulus_lupulus
Litchi_chinensis
Conservation
HG065
POPTR_0002s09080
Populus_trichocarpa
Panax_ginseng
Carya_cathayensis
Gossypium_raimondii
Conservation

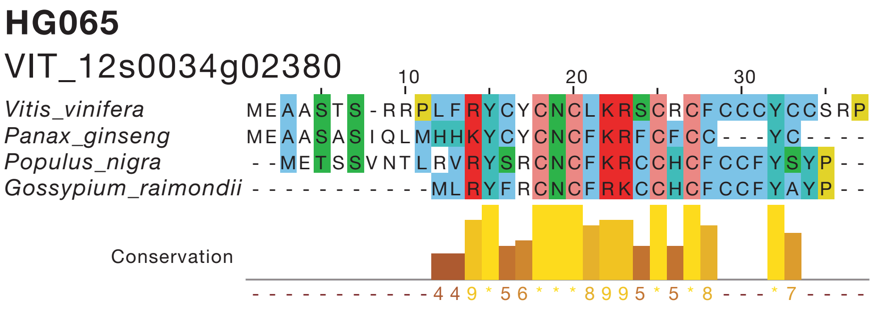
HG065
VIT_12s0034g02380
Vitis_vinifera
Panax_ginseng
Populus_nigra
Gossypium_raimondii
Conservation

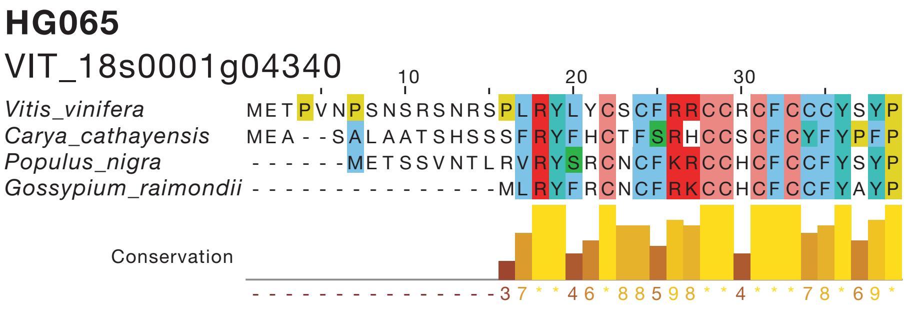
HG065
VIT_18s0001g04340
Vitis_vinifera
Carya_cathayensis
Populus_nigra
Gossypium_raimondii
Conservation

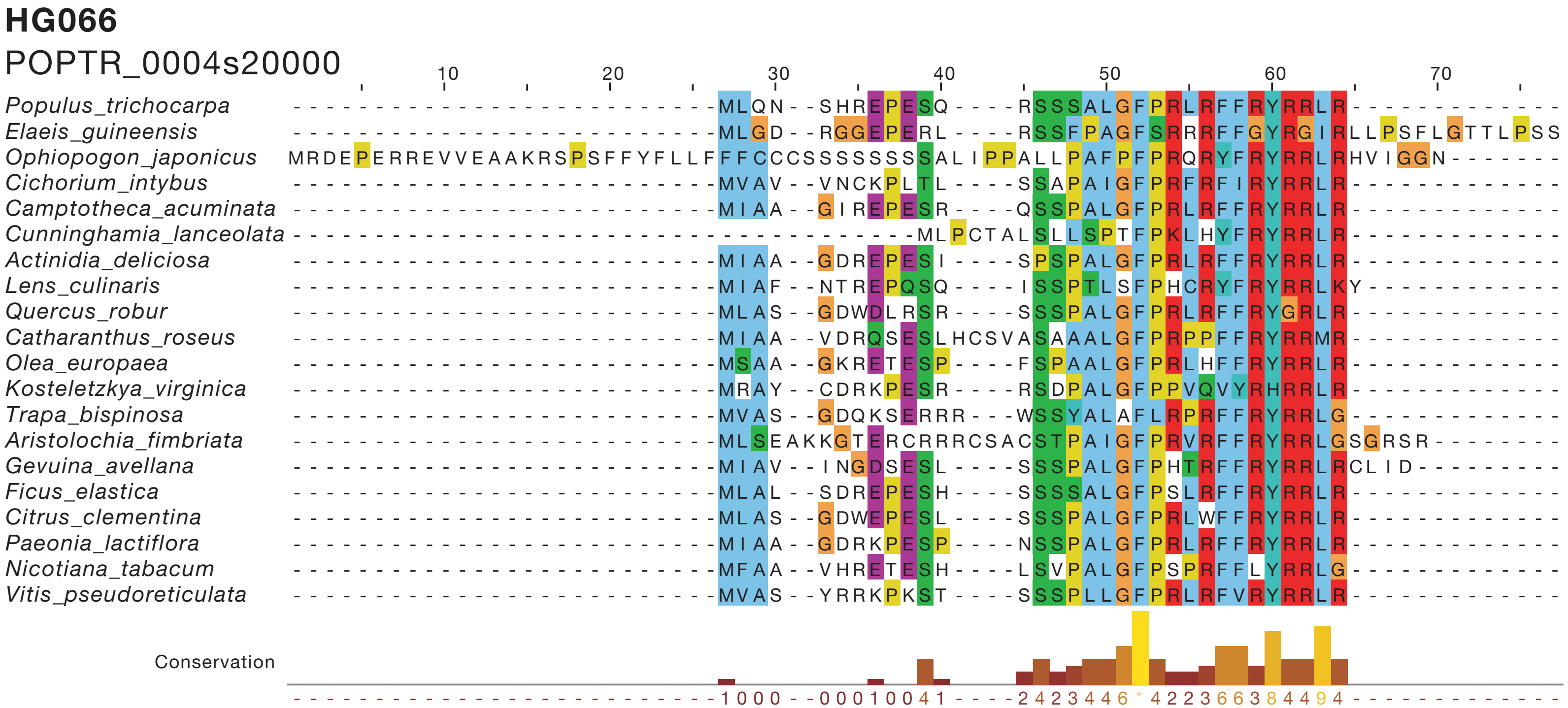
HG066
POPTR_0004s20000
Populus_trichocarpa
Elaeis_guineensis
Ophiopogon_japonicus
Cichorium_intybus
Camptotheca_acuminata
Cunninghamia_lanceolata
Actinidia_deliciosa
Lens_culinaris
Quercus_robur
Catharanthus_roseus
Olea_europaea
Kosteletzkya_virginica
Trapa_bispinosa
Aristolochia_fimbriata
Gevuina_avellana
Ficus_elastica
Citrus_clementina
Paeonia_lactiflora
Nicotiana_tabacum
Vitis_pseudoreticulata
Conservation

HG066

POPTR_0009s15140

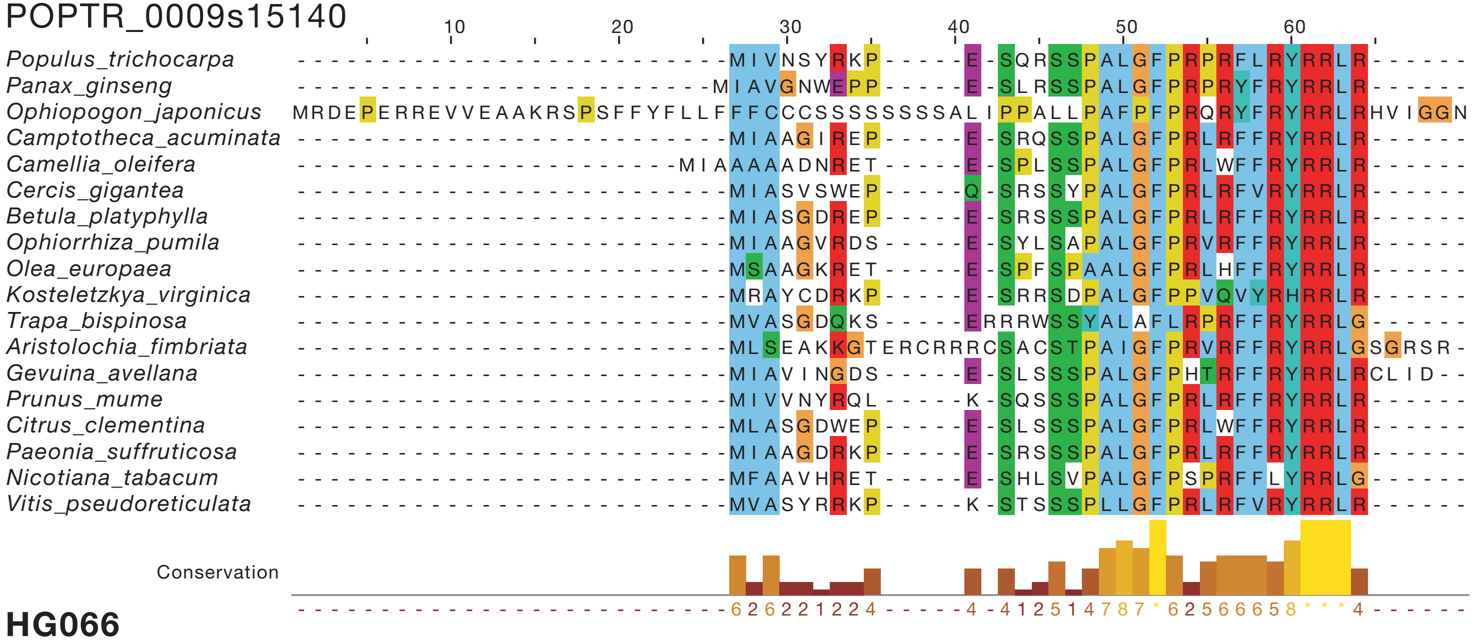

HG066

VIT_06s0061g01250

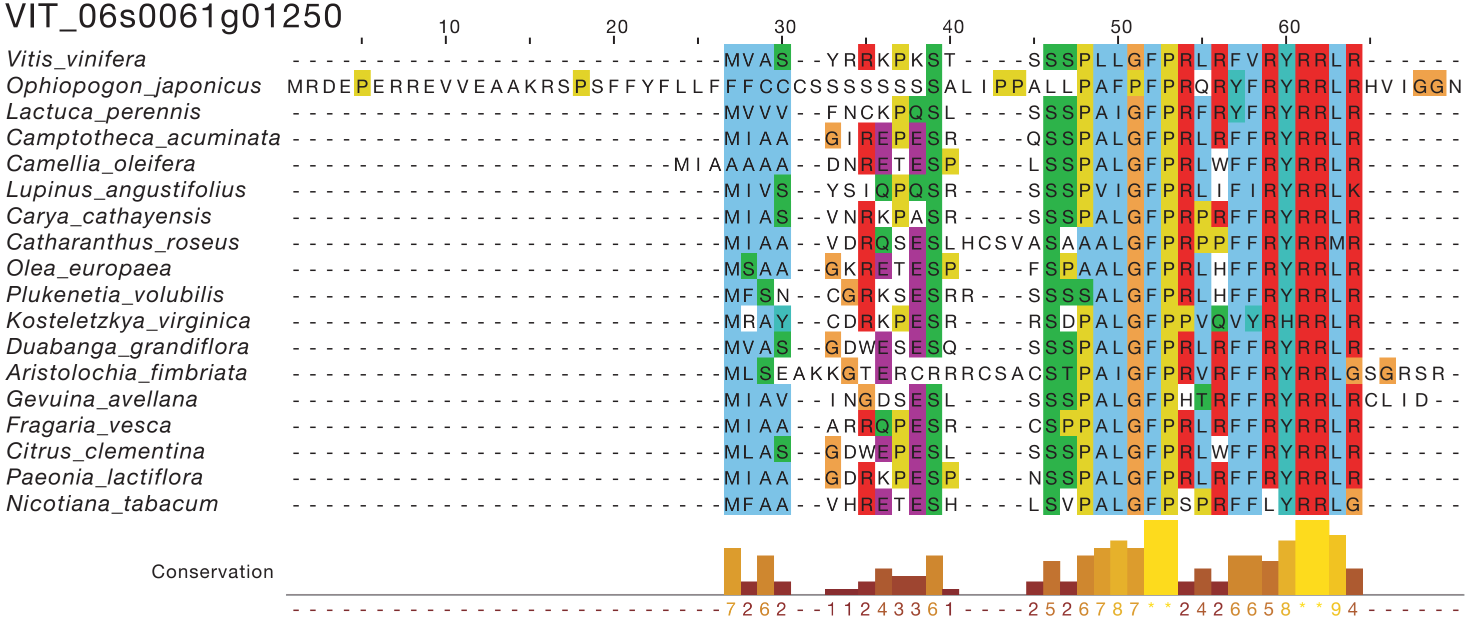

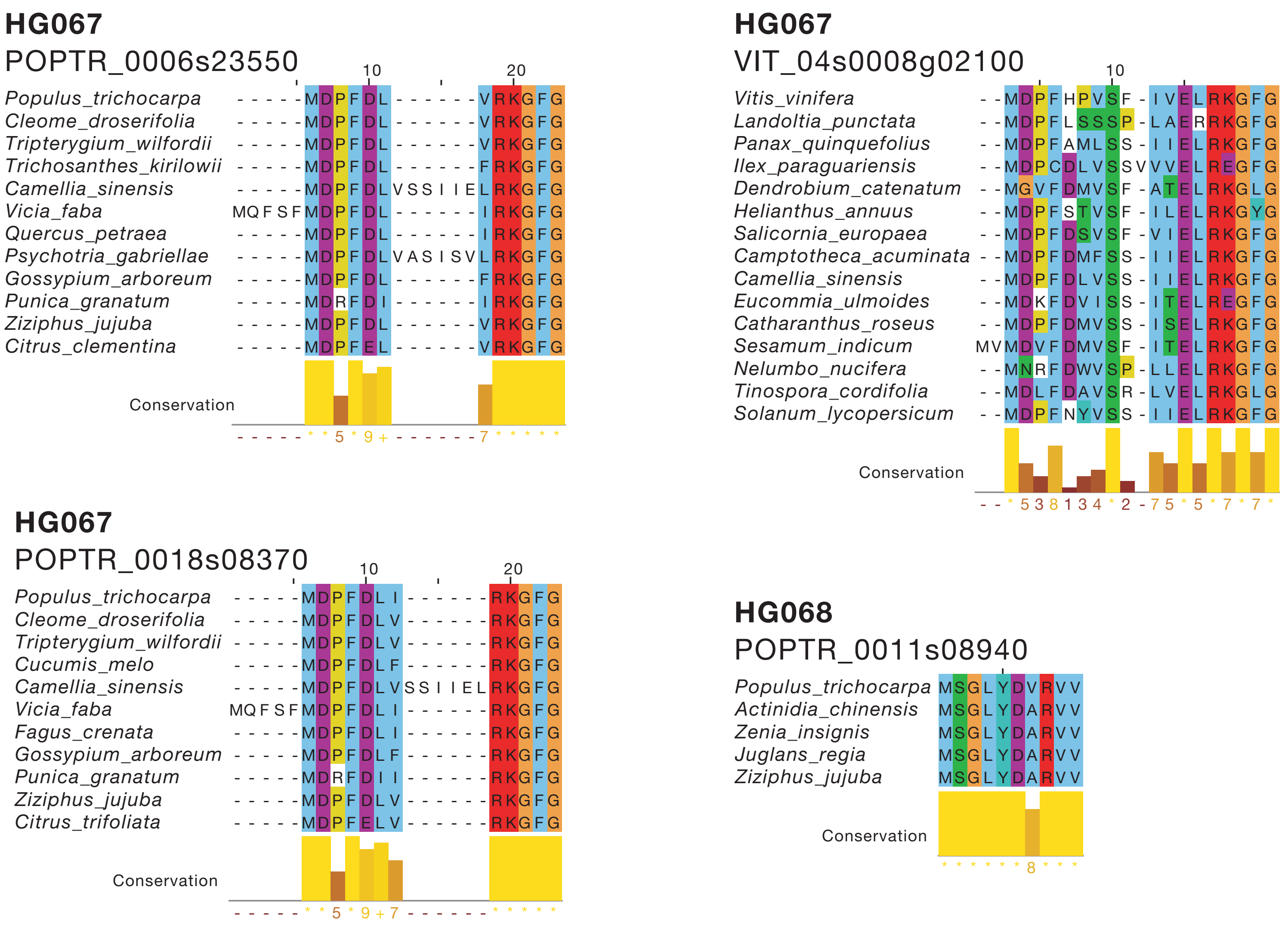

HG067
POPTR_0006s23550
10
20
Populus_trichocarpa -----MDPFDL------VRKGFG
Cleome_droserifolia -----MDPFDL------VRKGFG
Tripterygium_wilfordii -----MDPFDL------VRKGFG
Trichosanthes_kirilowii -----MDPFDL------FRKGFG
Camellia_sinensis -----MDPFDLVSSIIELRKGFG
Vicia_faba MQFSFMDPFDL------IRKGFG
Quercus_petraea -----MDPFDL------IRKGFG
Psychotria_gabriellae -----MDPFDLVASISVLRKGFG
Gossypium_arboreum -----MDPFDL------FRKGFG
Punica_granatum -----MDRFDI------IRKGFG
Ziziphus_jujuba -----MDPFDL------VRKGFG
Citrus_clementina -----MDPFEL------VRKGFG
Conservation
-----**5*9+------7*****
HG067
VIT_04s0008g02100
10
Vitis_vinifera --MDPFHPVSF-IVELRKGFG
Landoltia_punctata --MDPFLSSSP-LAERRKGFG
Panax_quinquefolius --MDPFAMLSS-IIELRKGFG
Ilex_paraguariensis --MDPCDLVSSVVVELREGFG
Dendrobium_catenatum --MGVFDMVSF-ATELRKGLG
Helianthus_annuus --MDPFSTVSF-ILELRKGYG
Salicornia_europaea --MDPFDSVSF-VIELRKGFG
Camptotheca_acuminata --MDPFDMFSS-IIELRKGFG
Camellia_sinensis --MDPFDLVSS-IIELRKGFG
Eucommia_ulmoides --MDKFDVISS-ITELREGFG
Catharanthus_roseus --MDPFDMVSS-ISELRKGFG
Sesamum_indicum MVMDVFDMVSF-ITELRKGFG
Nelumbo_nucifera --MNRFDWVSP-LLELRKGFG
Tinospora_cordifolia --MDLFDAVSR-LVELRKGLG
Solanum_lycopersicum --MDPFNYVSS-IIELRKGFG
Conservation
--*538134*2-75*5*7*7*
HG067
POPTR_0018s08370
10
20
Populus_trichocarpa -----MDPFDLI------RKGFG
Cleome_droserifolia -----MDPFDLV------RKGFG
Tripterygium_wilfordii -----MDPFDLV------RKGFG
Cucumis_melo -----MDPFDLF------RKGFG
Camellia_sinensis -----MDPFDLVSSIIELRKGFG
Vicia_faba MQFSFMDPFDLI------RKGFG
Fagus_crenata -----MDPFDLI------RKGFG
Gossypium_arboreum -----MDPFDLF------RKGFG
Punica_granatum -----MDRFDII------RKGFG
Ziziphus_jujuba -----MDPFDLV------RKGFG
Citrus_trifoliata -----MDPFELV------RKGFG
Conservation
-----**5*9+7------*****
HG068
POPTR_0011s08940
Populus_trichocarpa MSGLYDVRVV
Actinidia_chinensis MSGLYDARVV
Zenia_insignis MSGLYDARVV
Juglans_regia MSGLYDARVV
Ziziphus_jujuba MSGLYDARVV
Conservation
******8***

HG069

POPTR_0013s05200

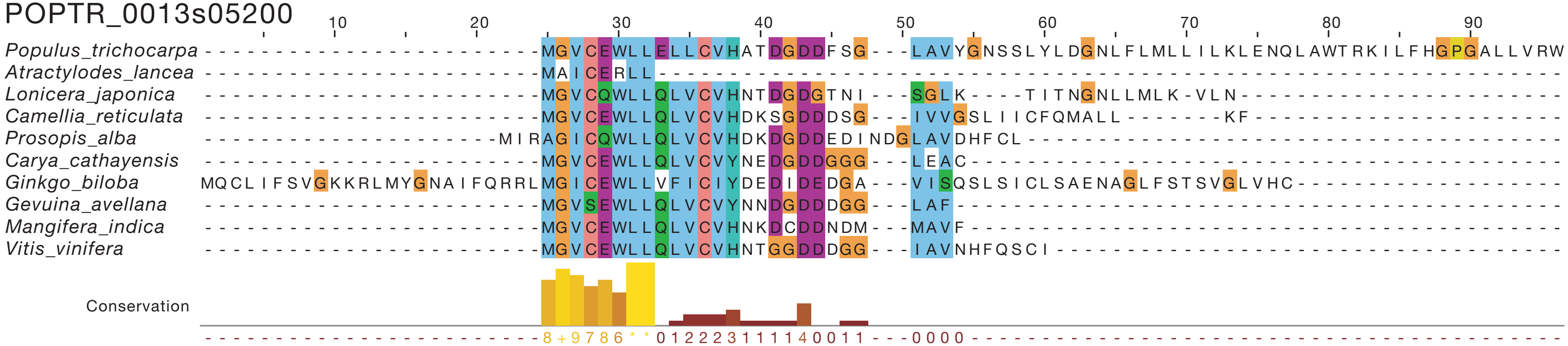

HG069

VIT_08s0032g00280

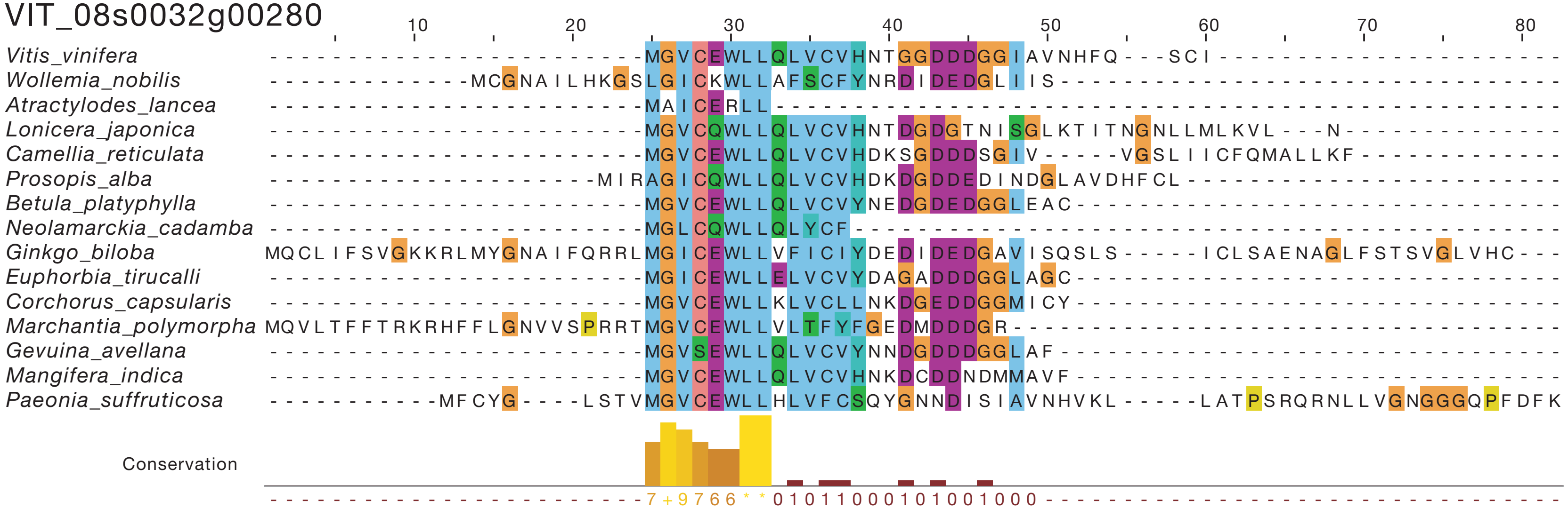

HG070

AT3G52490

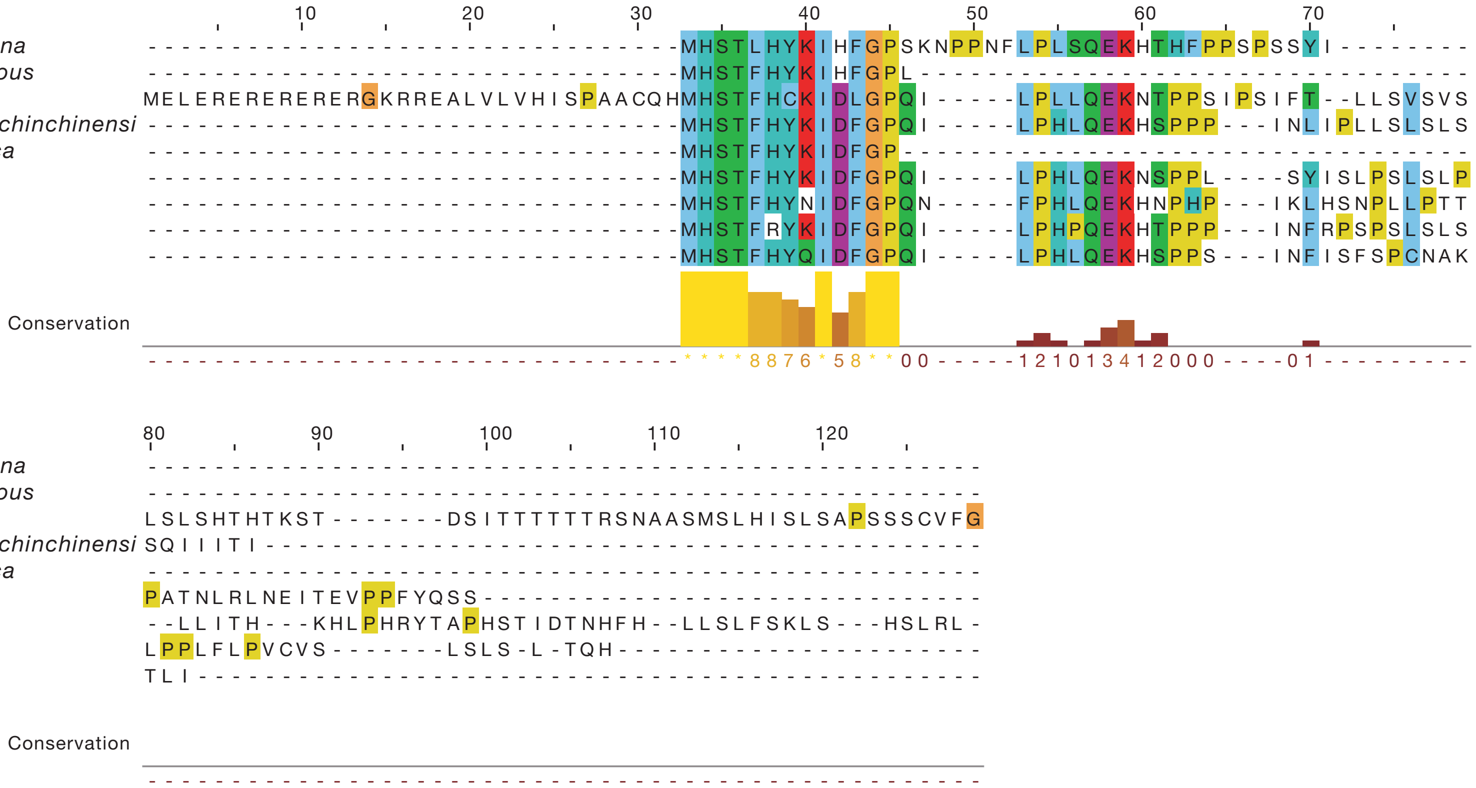

HG071

AT3G61970

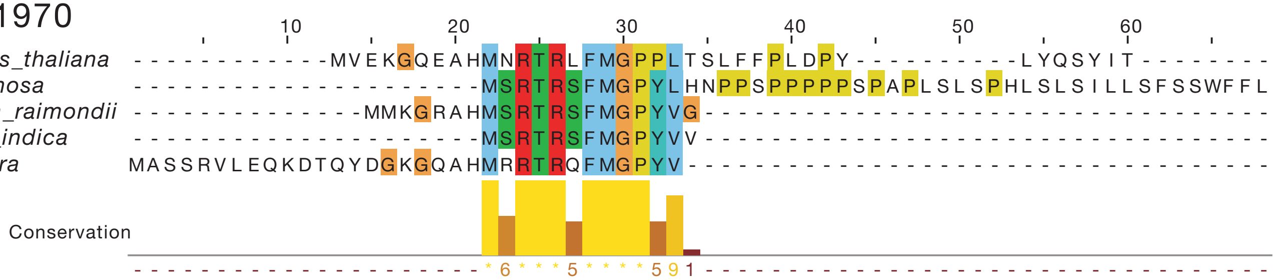

HG072.1
AT4G34610

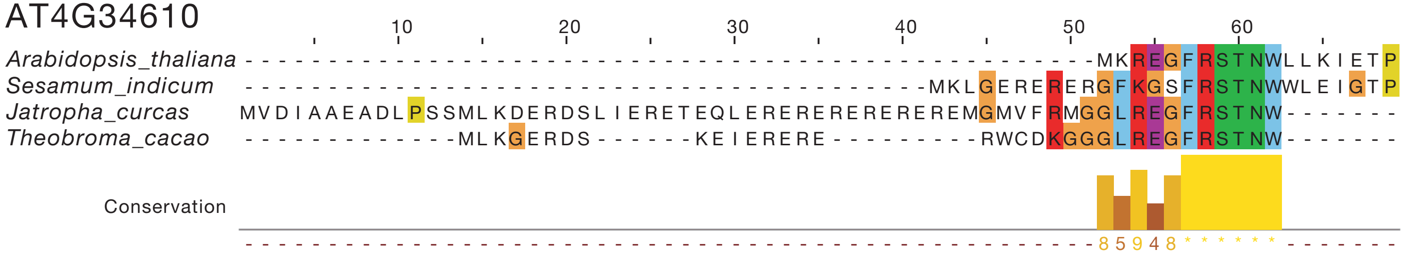

HG072.2
VIT_03s0038g00050

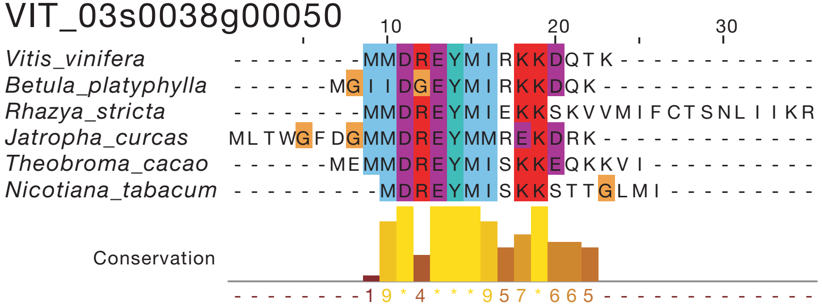

HG073
AT5G53660

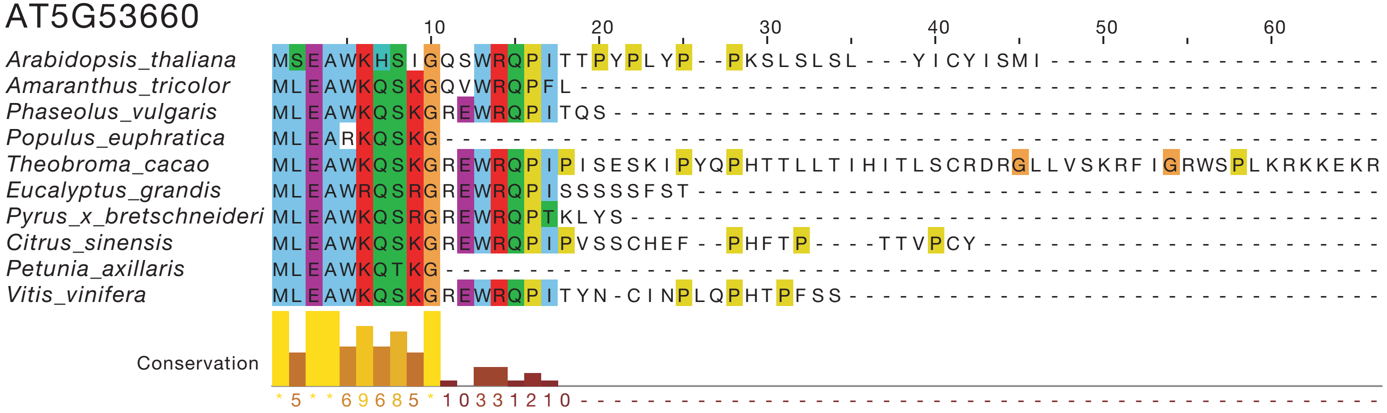

HG074

LOC_Os04g36058

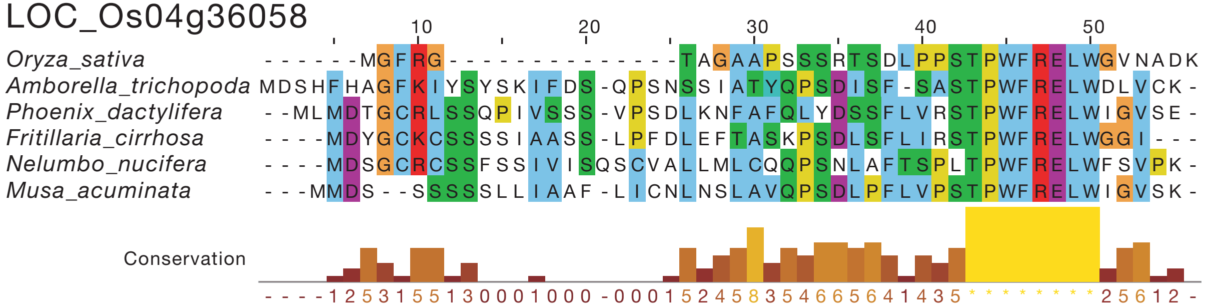

HG075

LOC_Os11g16280

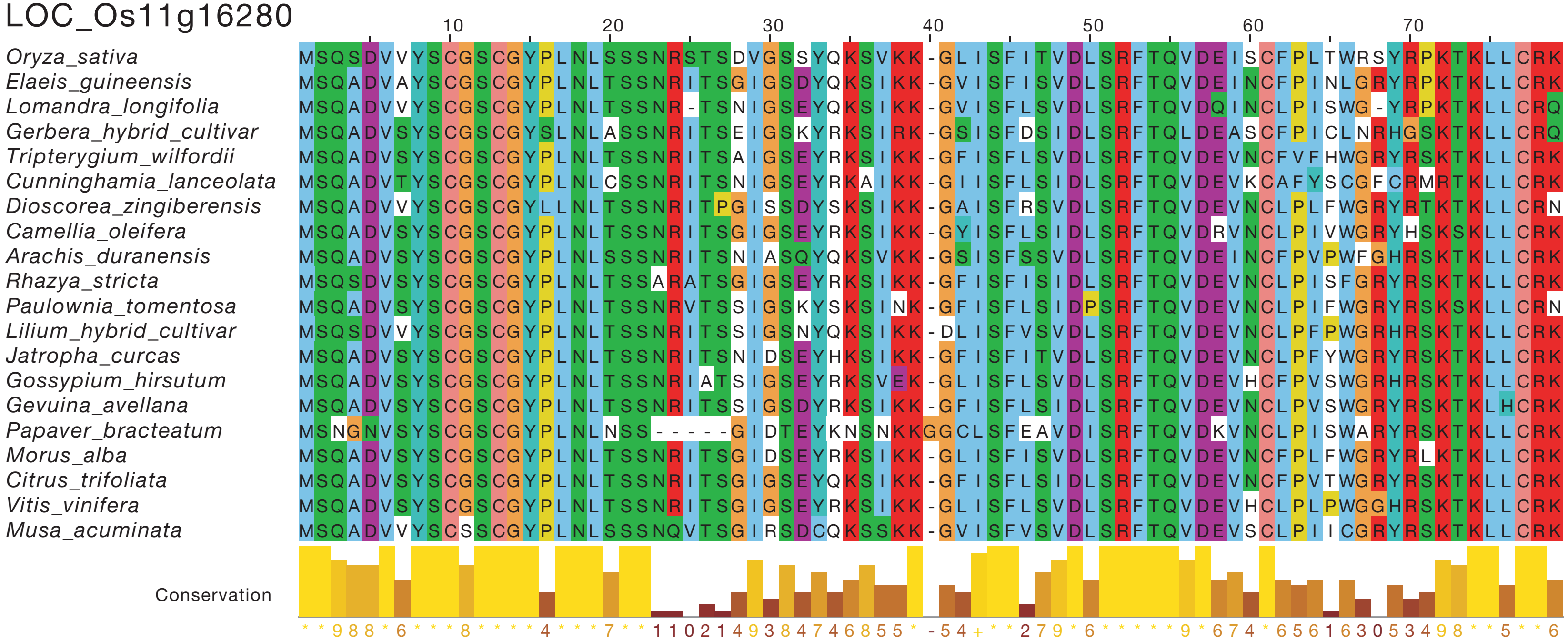

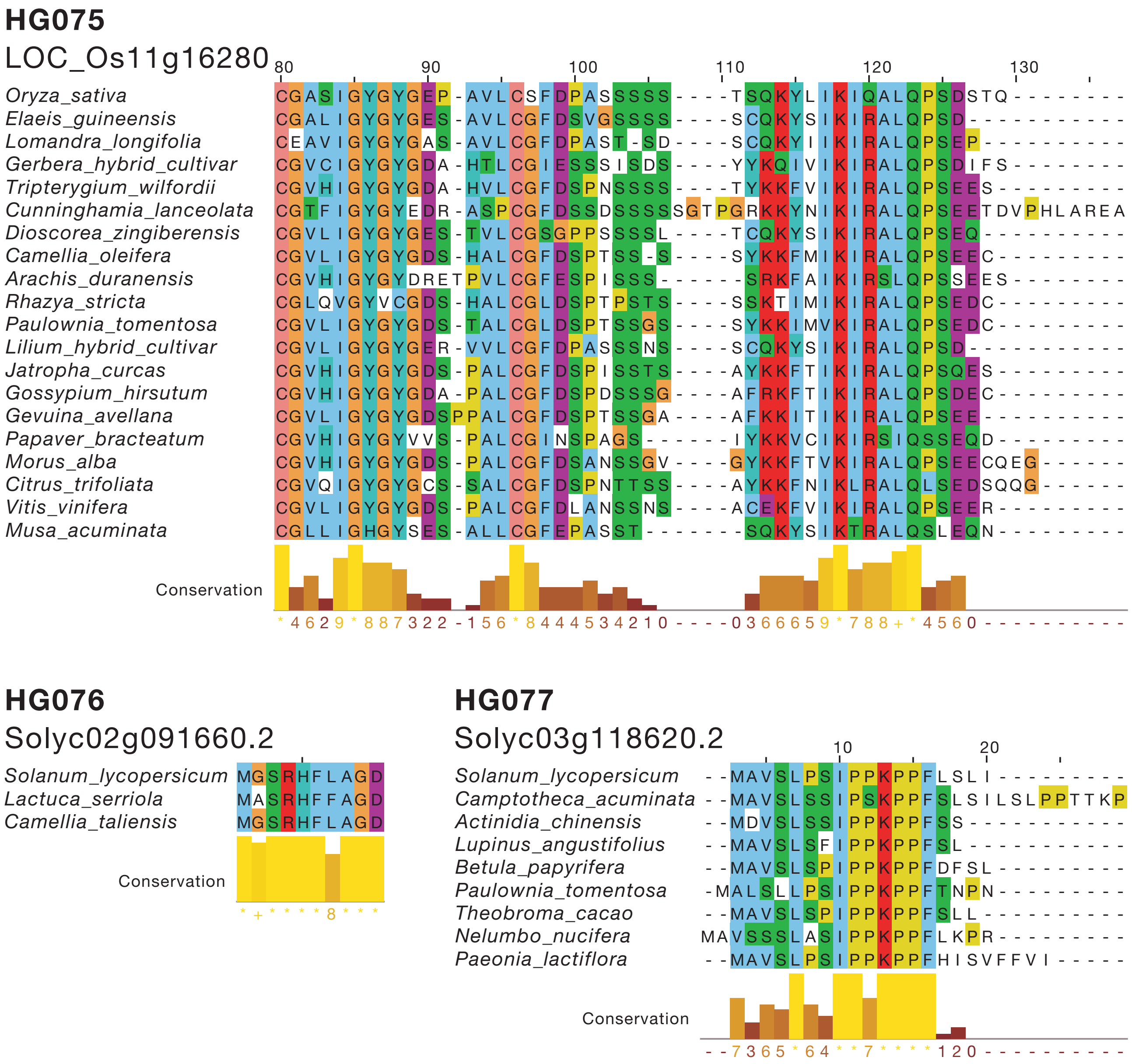
HG075
LOC_Os11g16280
Oryza_sativa
Elaeis_guineensis
Lomandra_longifolia
Gerbera_hybrid_cultivar
Tripterygium_wilfordii
Cunninghamia_lanceolata
Dioscorea_zingiberensis
Camellia_oleifera
Arachis_duranensis
Rhazya_stricta
Paulownia_tomentosa
Lilium_hybrid_cultivar
Jatropha_curcas
Gossypium_hirsutum
Gevuina_avellana
Papaver_bracteatum
Morus_alba
Citrus_trifoliata
Vitis_vinifera
Musa_acuminata
Conservation
HG076
Solyc02g091660.2
Solanum_lycopersicum
Lactuca_serriola
Camellia_taliensis
Conservation
HG077
Solyc03g118620.2
Solanum_lycopersicum
Camptotheca_acuminata
Actinidia_chinensis
Lupinus_angustifolius
Betula_papyrifera
Paulownia_tomentosa
Theobroma_cacao
Nelumbo_nucifera
Paeonia_lactiflora
Conservation

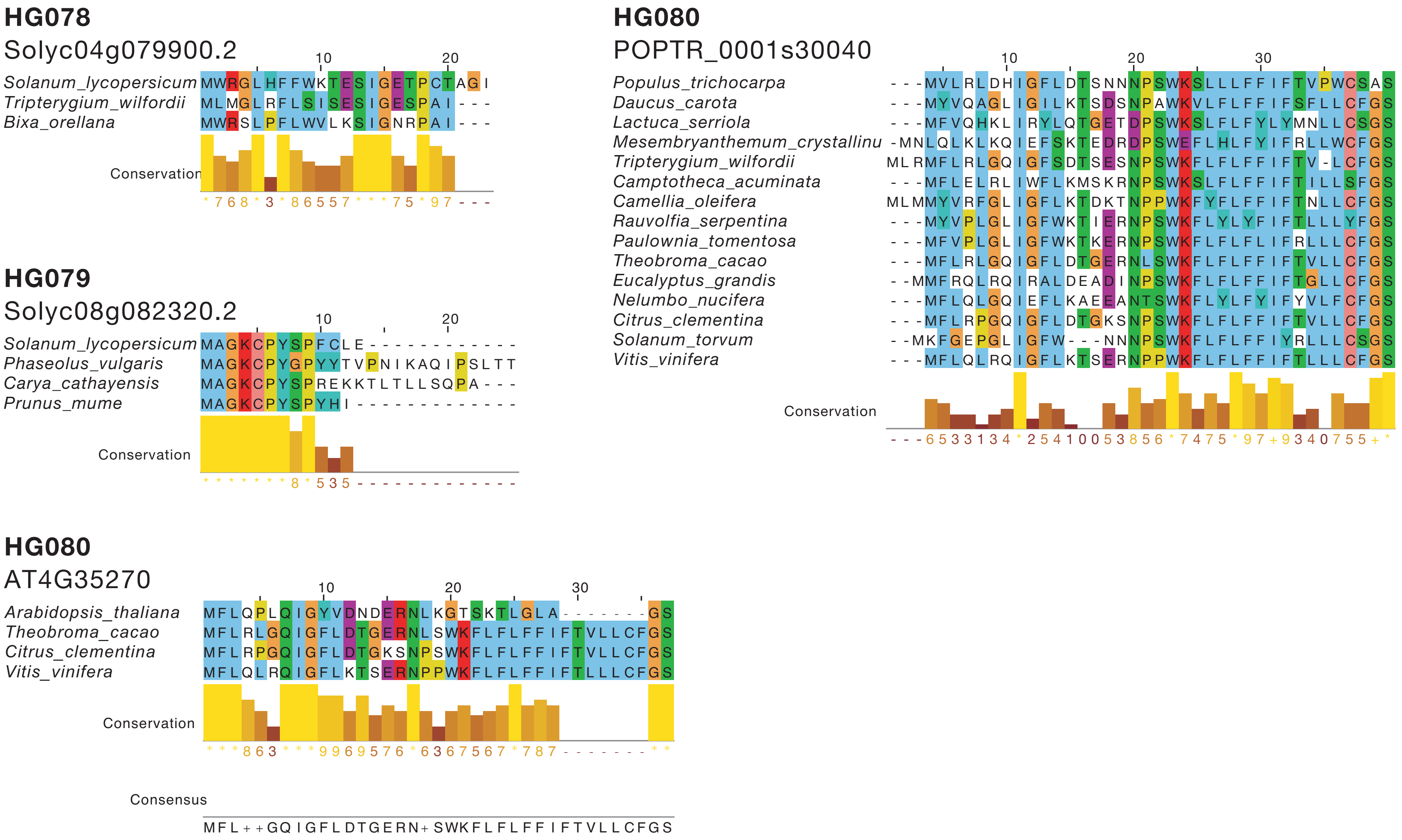
HG078
Solyc04g079900.2
Solanum_lycopersicum
Tripterygium_wilfordii
Bixa_orellana
Conservation
HG079
Solyc08g082320.2
Solanum_lycopersicum
Phaseolus_vulgaris
Carya_cathayensis
Prunus_mume
Conservation
HG080
AT4G35270
Arabidopsis_thaliana
Theobroma_cacao
Citrus_clementina
Vitis_vinifera
Conservation
Consensus
MFL++GQIGFLDTGERN+SWKFLFLFFIFTVLLCFGS
HG080
POPTR_0001s30040
Populus_trichocarpa
Daucus_carota
Lactuca_serriola
Mesembryanthemum_crystallinu
Tripterygium_wilfordii
Camptotheca_acuminata
Camellia_oleifera
Rauvolfia_serpentina
Paulownia_tomentosa
Theobroma_cacao
Eucalyptus_grandis
Nelumbo_nucifera
Citrus_clementina
Solanum_torvum
Vitis_vinifera
Conservation

HG080

POPTR_0004s21550

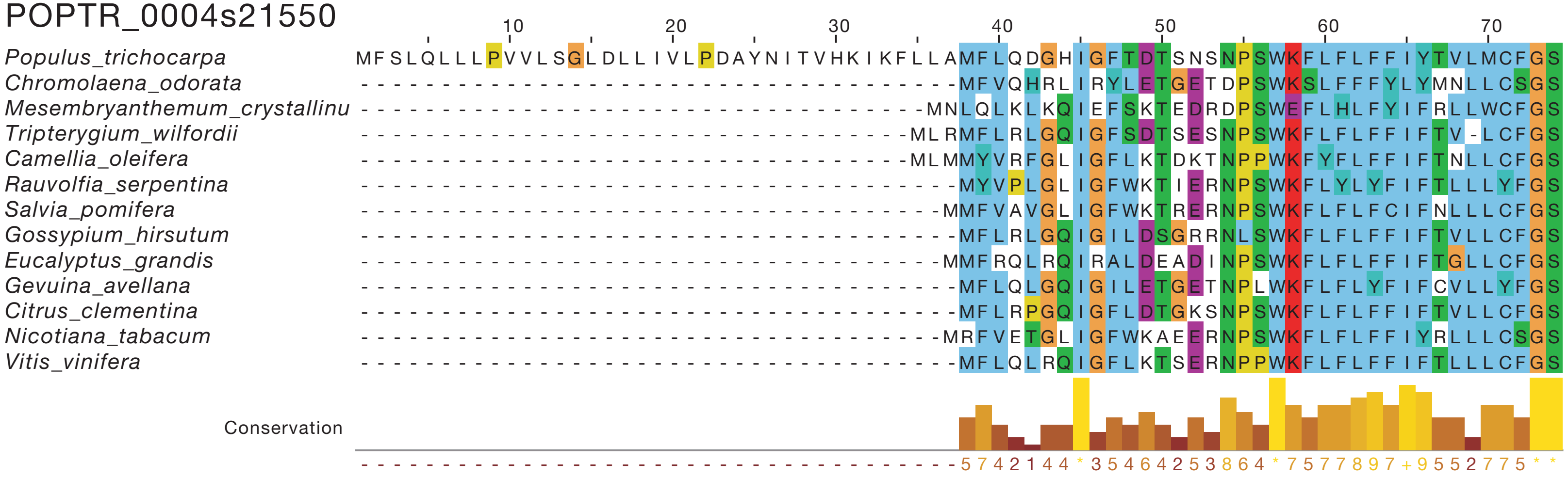

HG080

POPTR_0009s16830

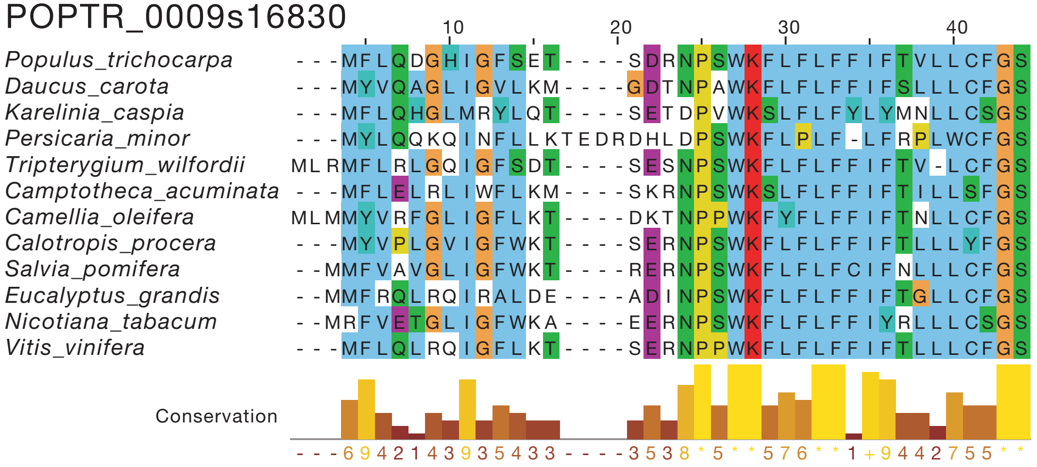

HG081

POPTR_0011s09450

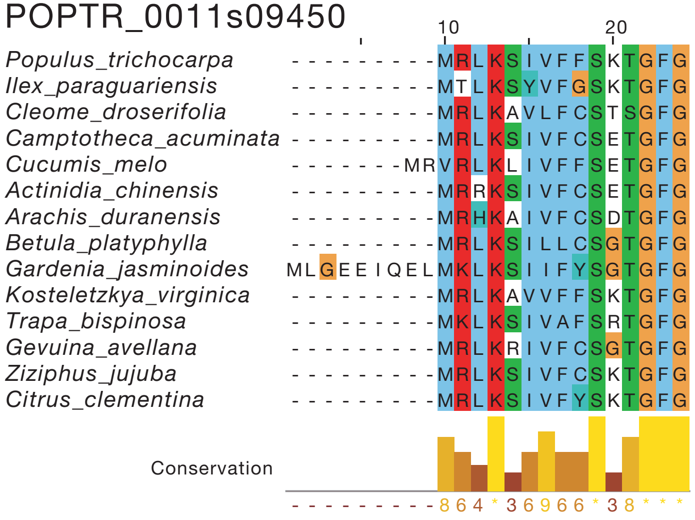

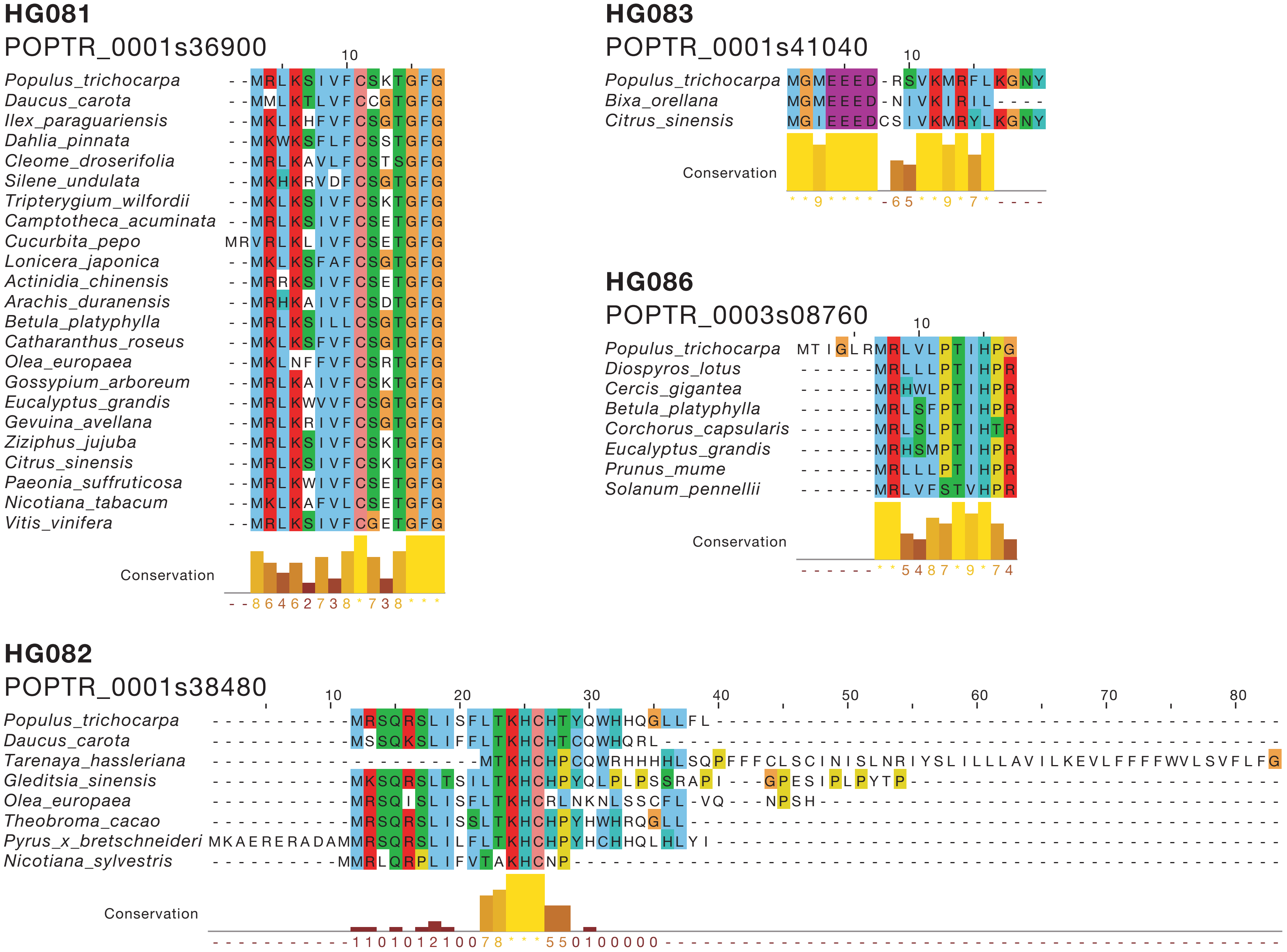
HG081
POPTR_0001s36900
10
Populus_trichocarpa --MRLKSIVFCSKTGFG
Daucus_carota --MMLKTLVFCCGTGFG
Ilex_paraguariensis --MKLKHFVFCSGTGFG
Dahlia_pinnata --MKWKSFLFCSSTGFG
Cleome_droserifolia --MRLKAVLFCSTSGFG
Silene_undulata --MKHKRVDFCSGTGFG
Tripterygium_wilfordii --MKLKSIVFCSKTGFG
Camptotheca_acuminata --MRLKSIVFCSETGFG
Cucurbita_pepo MRVRLKLIVFCSETGFG
Lonicera_japonica --MKLKSFAFCSGTGFG
Actinidia_chinensis --MRRKSIVFCSETGFG
Arachis_duranensis --MRHKAIVFCSDTGFG
Betula_platyphylla --MRLKSILLCSGTGFG
Catharanthus_roseus --MKLKSFVFCSGTGFG
Olea_europaea --MKLNFFVFCSRTGFG
Gossypium_arboreum --MRLKAIVFCSKTGFG
Eucalyptus_grandis --MRLKWVVFCSGTGFG
Gevuina_avellana --MRLKRIVFCSGTGFG
Ziziphus_jujuba --MRLKSIVFCSKTGFG
Citrus_sinensis --MRLKSIVFCSKTGFG
Paeonia_suffruticosa --MRLKWIVFCSETGFG
Nicotiana_tabacum --MKLKAFVLCSETGFG
Vitis_vinifera --MRLKSIVFCGETGFG
Conservation
--86462738*738***
HG083
POPTR_0001s41040
10
Populus_trichocarpa MGMEEED-RSVKMRFLKGNY
Bixa_orellana MGMEEED-NIVKIRIL----
Citrus_sinensis MGIEEEDCSIVKMRYLKGNY
Conservation
9**-65**9*7*----
HG086
POPTR_0003s08760
10
Populus_trichocarpa MTIGLRMRLVLPTIHPG
Diospyros_lotus ------MRLLLPTIHPR
Cercis_gigantea ------MRHWLPTIHPR
Betula_platyphylla ------MRLSFPTIHPR
Corchorus_capsularis ------MRLSLPTIHTR
Eucalyptus_grandis ------MRHSMPTIHPR
Prunus_mume ------MRLLLPTIHPR
Solanum_pennellii ------MRLVFSTVHPR
Conservation
------**5487*9*74
HG082
POPTR_0001s38480
10 20 30 40 50 60 70 80
Populus_trichocarpa -----------MRSQRSLISFLTKHCHTYQWHHQGLLFL--
Daucus_carota -----------MSSQKSLIFFLTKHCHTCQWHQRL---
Tarenaya_hassleriana --------------------MTKHCHPCQWRHHHHLSQPFFFCLSCINISLNRIYSLILLLAVILKEVLFFFFWVLSVFLFG
Gleditsia_sinensis -----------MKSQRSLTSILTKHCHPYQLPLPSSRAPI---GPESIPLPYTP----------------------------
Olea_europaea -----------MRSQISLISFLTKHCRLNKNLSSCFL-VQ---NPSH-----------------------------------
Theobroma_cacao -----------MRSQRSLISSLTKHCHPYHWHRQGLL---
Pyrus_x_bretschneideri MKAERERADAMMRSQRSLILFLTKHCHPYHCHHQLHLYI---
Nicotiana_sylvestris ----------MMRLQRPLIFVTAKHCNP---
Conservation
-----------110101210078***550100000---

HG087

POPTR_0004s03900

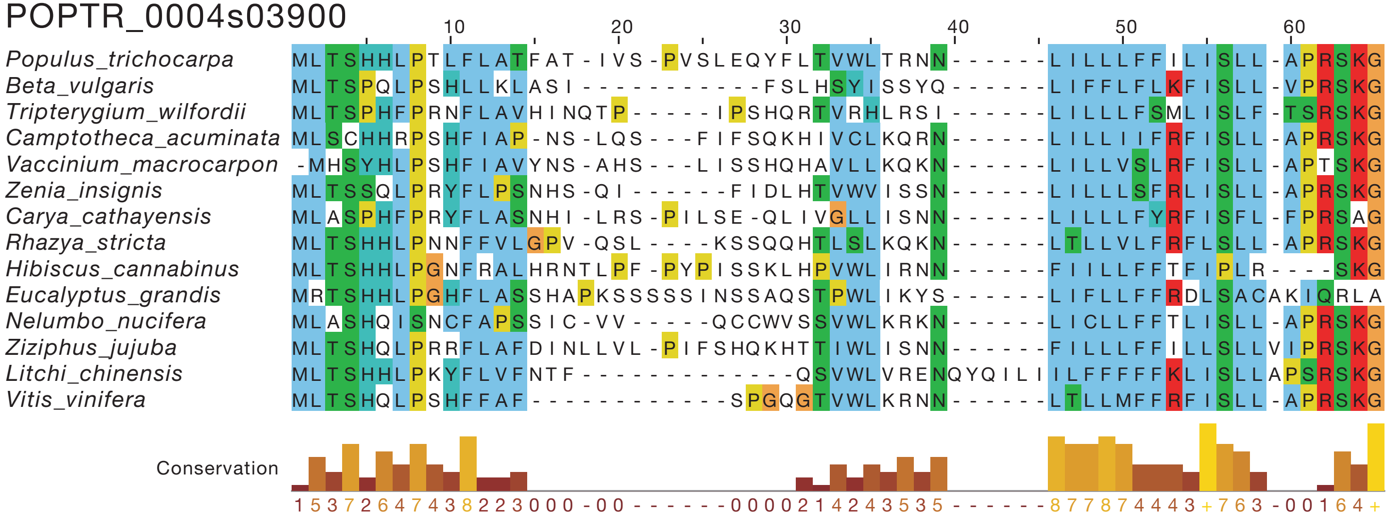

HG087

POPTR_0011s04730

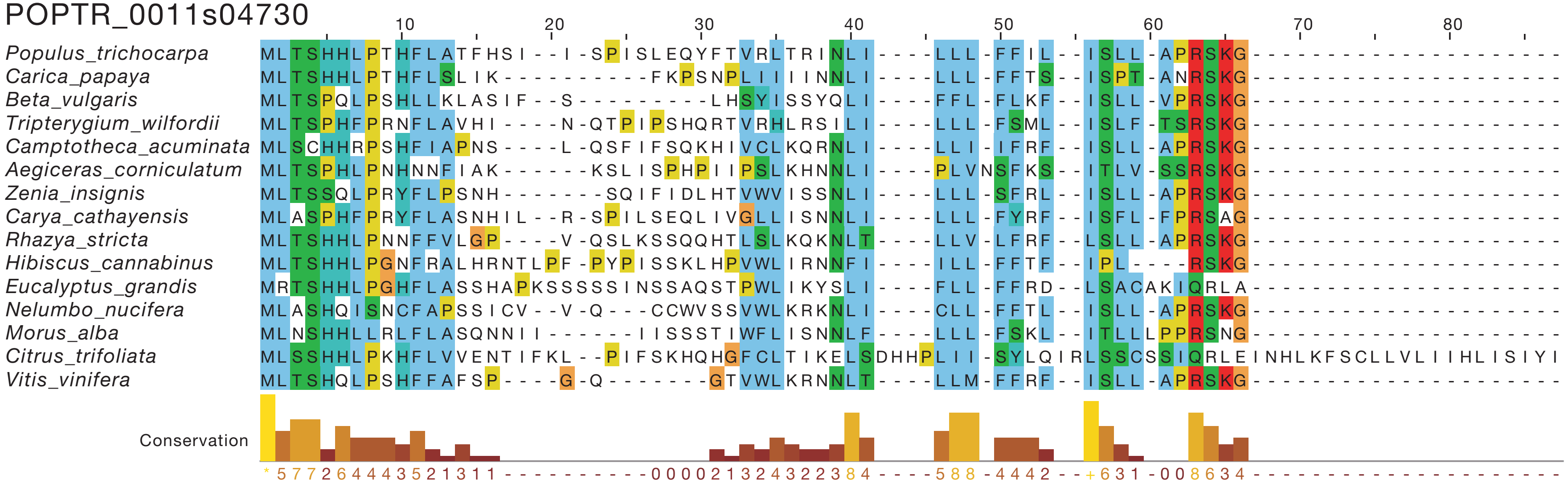

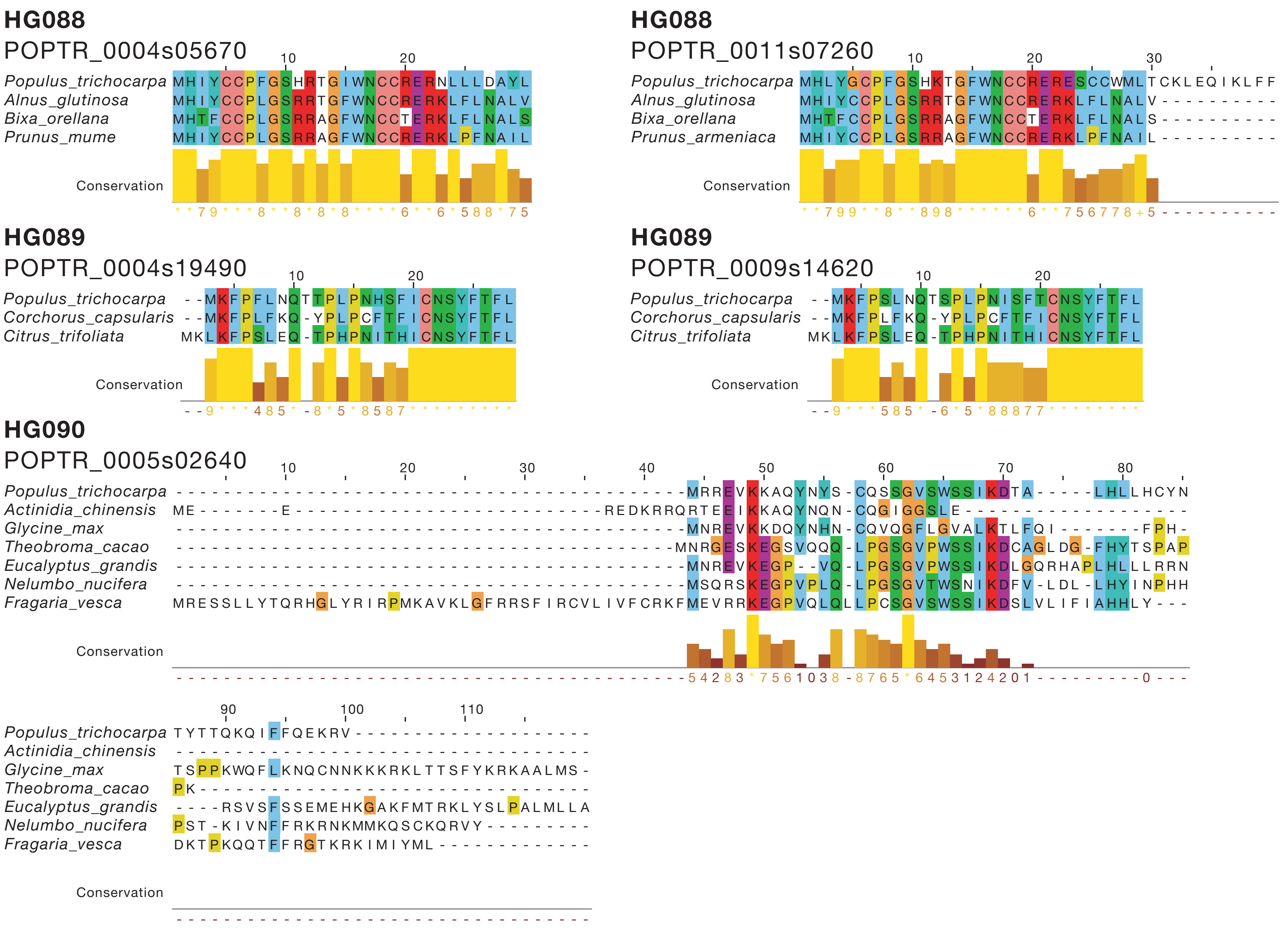
HG088
POPTR_0004s05670
Populus_trichocarpa
Alnus_glutinosa
Bixa_orellana
Prunus_mume
Conservation
HG088
POPTR_0011s07260
Populus_trichocarpa
Alnus_glutinosa
Bixa_orellana
Prunus_armeniaca
Conservation
HG089
POPTR_0004s19490
Populus_trichocarpa
Corchorus_capsularis
Citrus_trifoliata
Conservation
HG089
POPTR_0009s14620
Populus_trichocarpa
Corchorus_capsularis
Citrus_trifoliata
Conservation
HG090
POPTR_0005s02640
Populus_trichocarpa
Actinidia_chinensis
Glycine_max
Theobroma_cacao
Eucalyptus_grandis
Nelumbo_nucifera
Fragaria_vesca
Conservation

HG091

POPTR_0005s07290

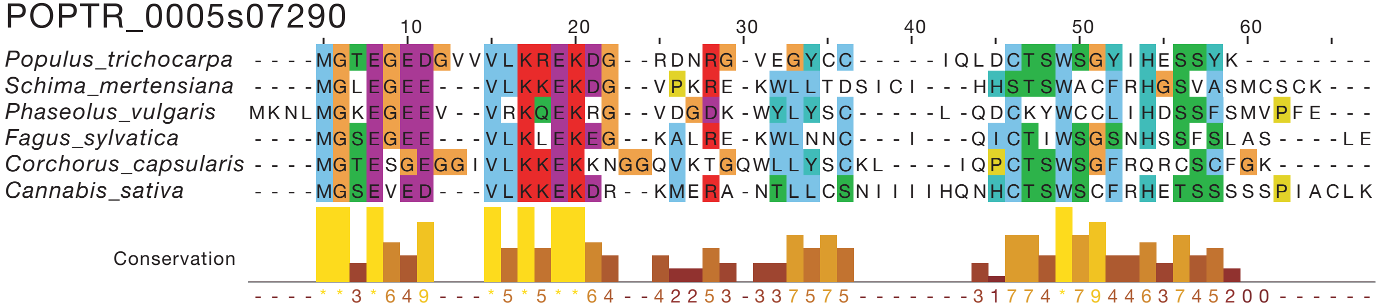

HG091

POPTR_0007s05010

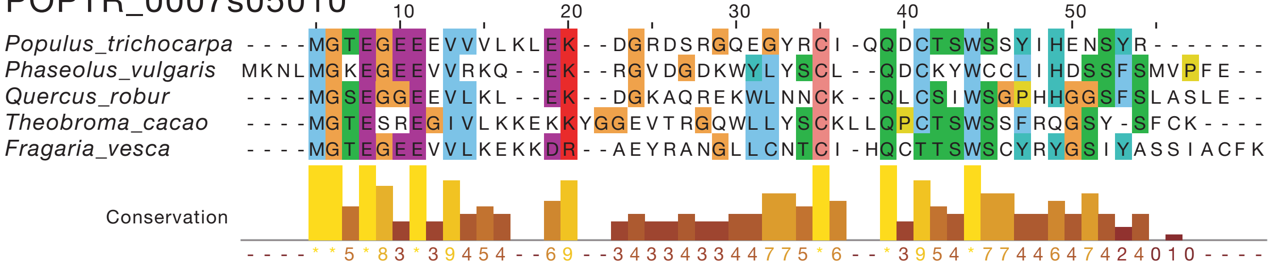

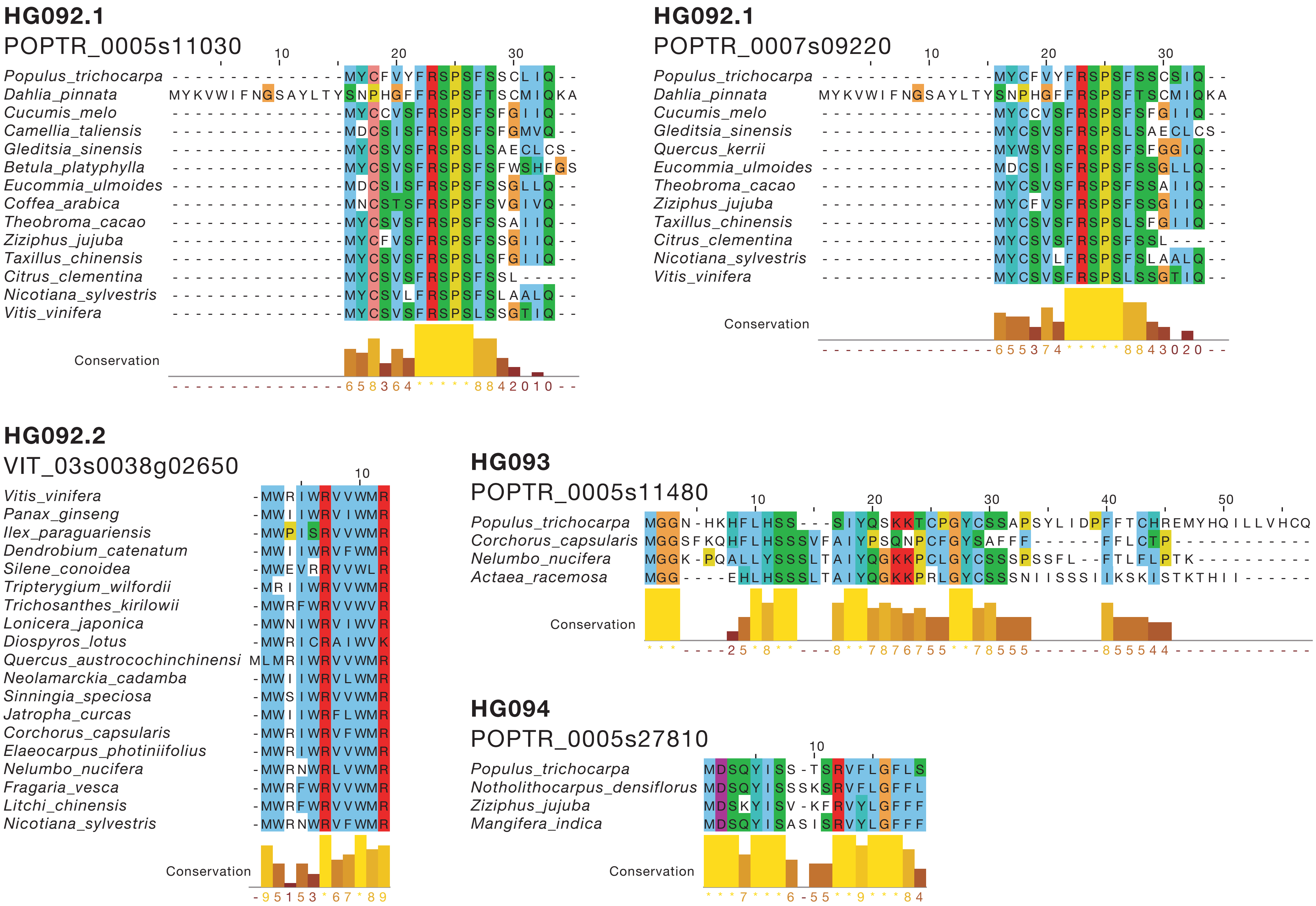
HG092.1
POPTR_0005s11030
Populus_trichocarpa
Dahlia_pinnata
Cucumis_melo
Camellia_taliensis
Gleditsia_sinensis
Betula_platyphylla
Eucommia_ulmoides
Coffea_arabica
Theobroma_cacao
Ziziphus_jujuba
Taxillus_chinensis
Citrus_clementina
Nicotiana_sylvestris
Vitis_vinifera
Conservation
HG092.1
POPTR_0007s09220
Populus_trichocarpa
Dahlia_pinnata
Cucumis_melo
Gleditsia_sinensis
Quercus_kerrii
Eucommia_ulmoides
Theobroma_cacao
Ziziphus_jujuba
Taxillus_chinensis
Citrus_clementina
Nicotiana_sylvestris
Vitis_vinifera
Conservation
HG092.2
VIT_03s0038g02650
Vitis_vinifera
Panax_ginseng
Ilex_paraguariensis
Dendrobium_catenatum
Silene_conoidea
Tripterygium_wilfordii
Trichosanthes_kirilowii
Lonicera_japonica
Diospyros_lotus
Quercus_austrocochinchinensis
Neolamarckia_cadamba
Sinningia_speciosa
Jatropha_curcas
Corchorus_capsularis
Elaeocarpus_photiniifolius
Nelumbo_nucifera
Fragaria_vesca
Litchi_chinensis
Nicotiana_sylvestris
Conservation
HG093
POPTR_0005s11480
Populus_trichocarpa
Corchorus_capsularis
Nelumbo_nucifera
Actaea_racemosa
Conservation
HG094
POPTR_0005s27810
Populus_trichocarpa
Notholithocarpus_densiflorus
Ziziphus_jujuba
Mangifera_indica
Conservation

HG095

POPTR_0006s07660

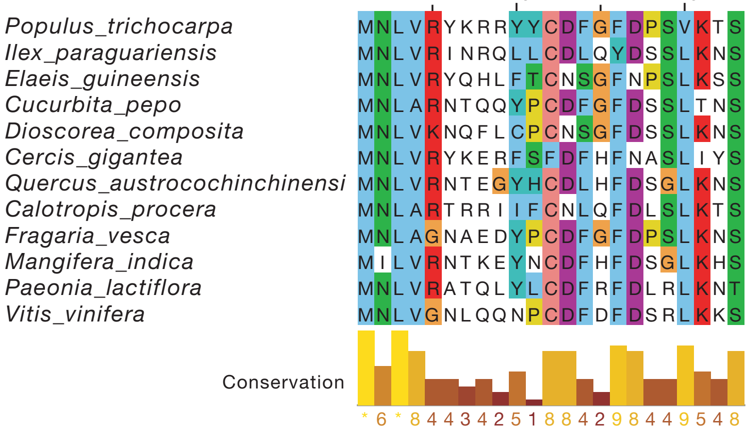

HG096

POPTR_0006s13860

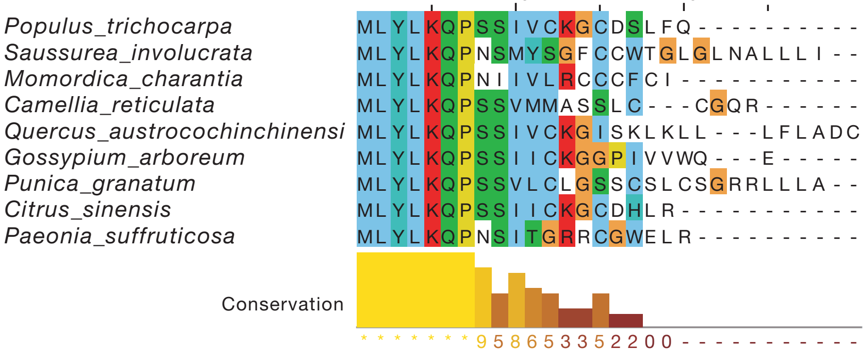

HG096

POPTR_0018s07360

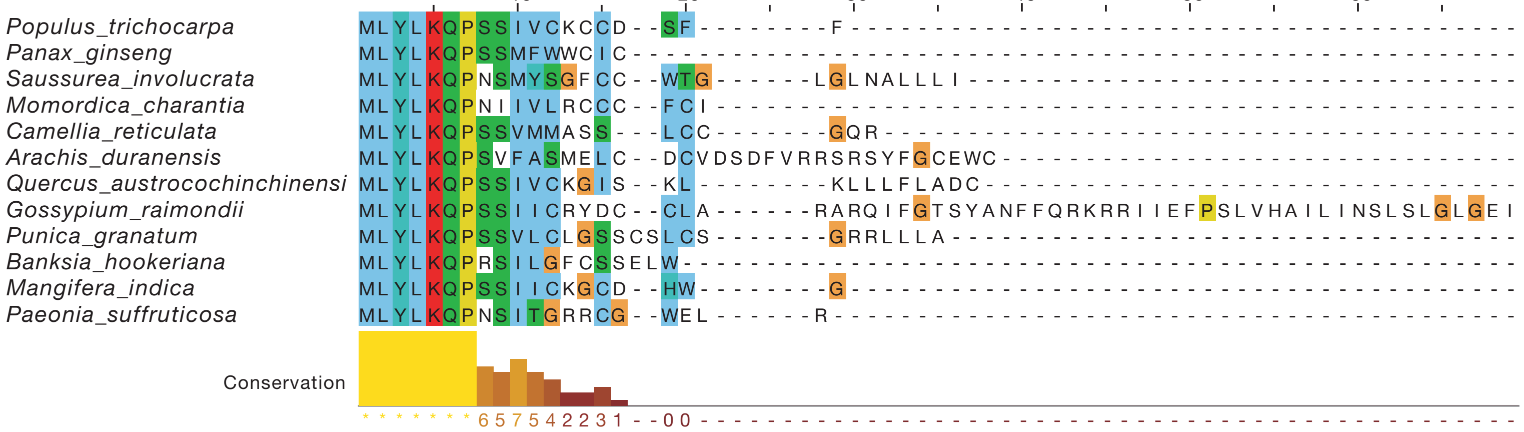

HG097

POPTR_0006s14740

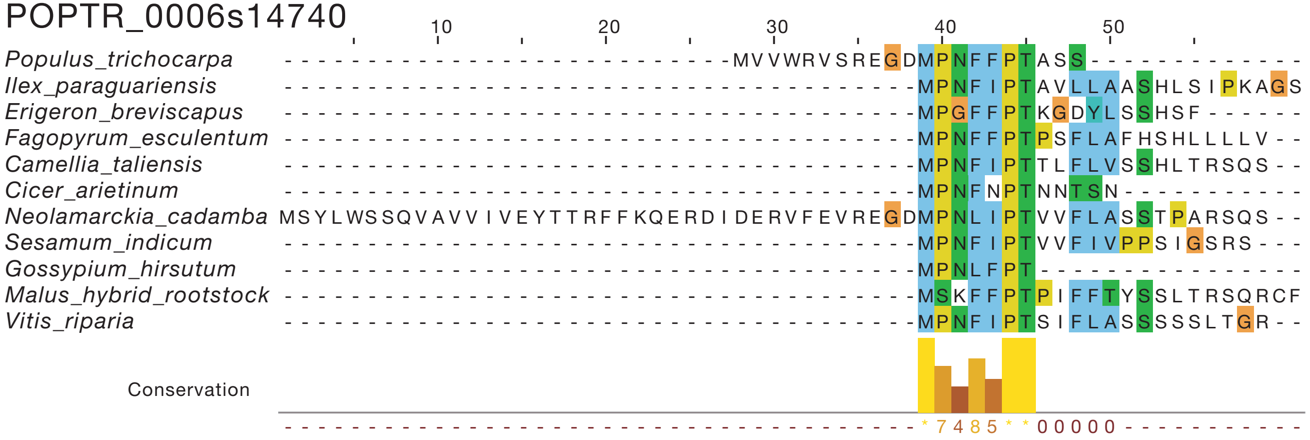

HG098

POPTR_0006s22890

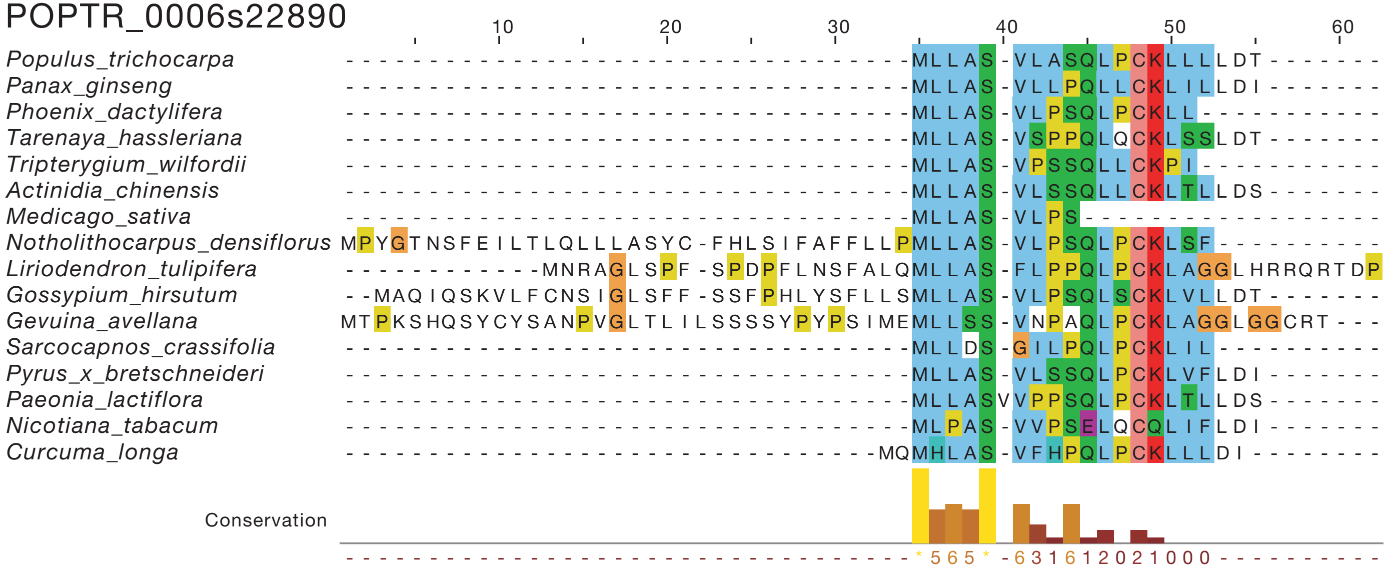

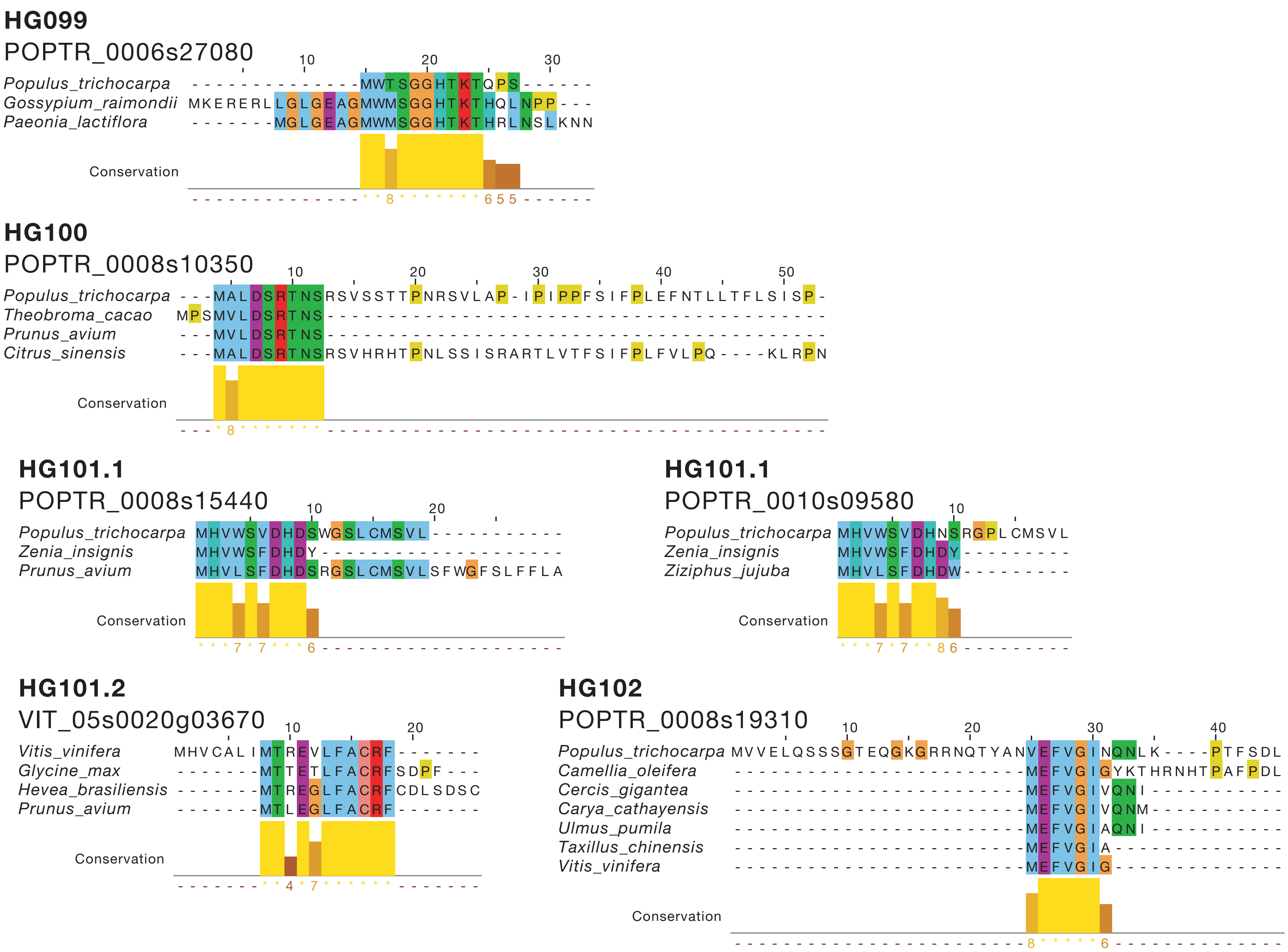
HG099
POPTR_0006s27080
Populus_trichocarpa ---------------MWTSGGHTKTQPS------
Gossypium_raimondii MKERERLLGLGEAGMWMSGGHTKTHQLNPP---
Paeonia_lactiflora -------MGLGEAGMWMSGGHTKTHRLNSLKNN
Conservation
HG100
POPTR_0008s10350
Populus_trichocarpa ---MALDSRTNSRSVSSTTPNRSVLAP-IPIPPFSIFPLEFNTLLTFLSISP-
Theobroma_cacao MPSMVLDSRTNS
Prunus_avium ---MVLDSRTNS
Citrus_sinensis ---MALDSRTNSRSVHRHTPNLSSISRARTLVTFSIFPLFVLPQ----KLRPN
Conservation
HG101.1
POPTR_0008s15440
Populus_trichocarpa MHVWSVDHDSWGSLCMSVL
Zenia_insignis MHVWSFDHDY
Prunus_avium MHVLSFDHDSRGSLCMSVLSFWGFSLFFLA
Conservation
HG101.1
POPTR_0010s09580
Populus_trichocarpa MHVWSVDHNSRGPLCMSVL
Zenia_insignis MHVWSFDHDY
Ziziphus_jujuba MHVLSFDHDW
Conservation
HG101.2
VIT_05s0020g03670
Vitis_vinifera MHVCALIMTREVLFACRF
Glycine_max -------MTTETLFACRFSDPF---
Hevea_brasiliensis -------MTREGLFACRFCDLSDSC
Prunus_avium -------MTLEGLFACRF
Conservation
HG102
POPTR_0008s19310
Populus_trichocarpa MVVELQSSSGTEQGKGRRNQTYANVEFVGINQNLK----PTFSDL
Camellia_oleifera MEFVGIGYKTHRNHTPAFPDL
Cercis_gigantea MEFVGIVQNI
Carya_cathayensis MEFVGIVQNM
Ulmus_pumila MEFVGIAQNI
Taxillus_chinensis MEFVGIA
Vitis_vinifera MEFVGIG
Conservation

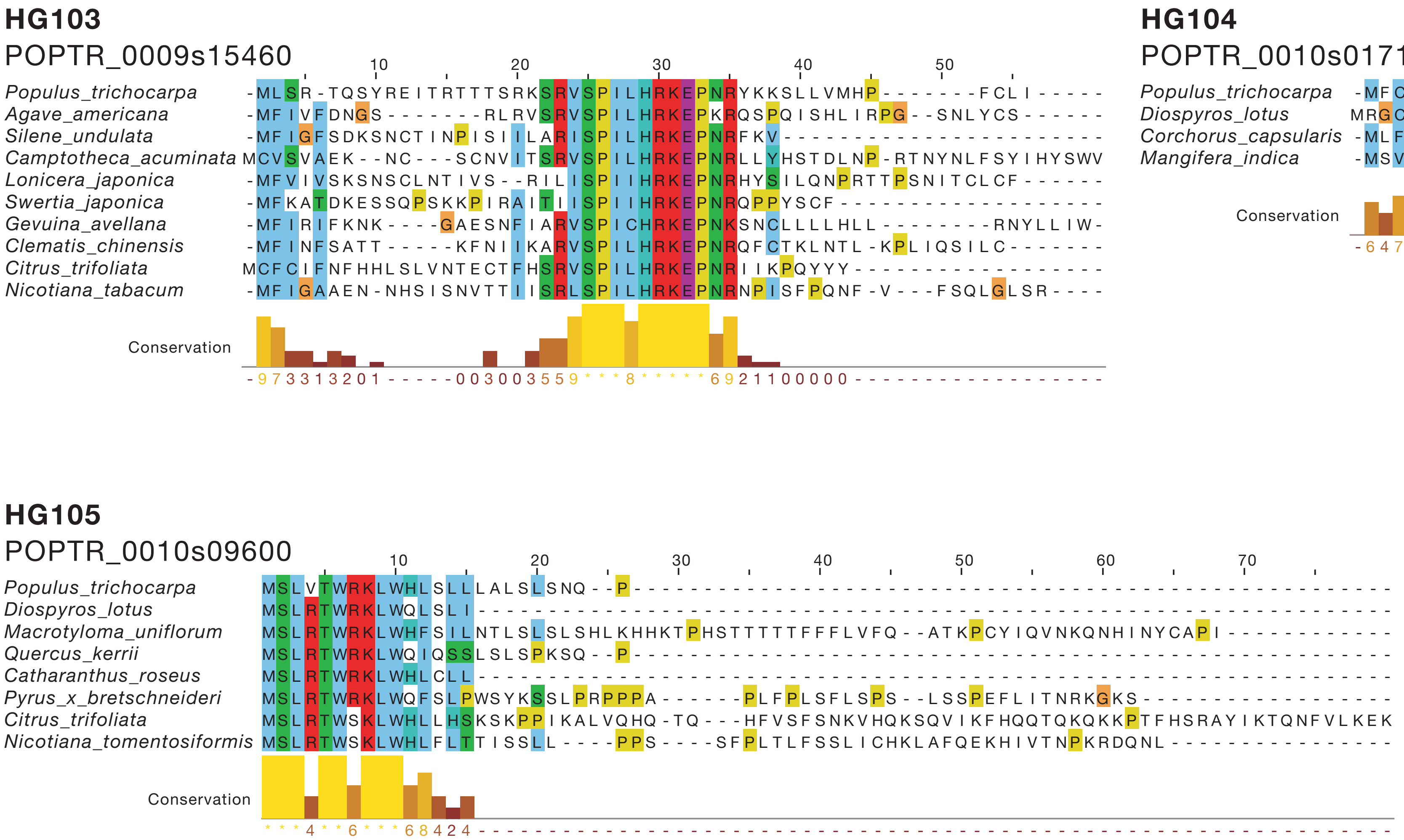
HG103
POPTR_0009s15460
10 20 30 40 50
Populus_trichocarpa -MLSR-TQSYREITRTTTSRKSRVSPILHRKEPNRYKKSLLVMHP------FCLI-----
Agave_americana -MFIVFDNGS-------RLRVSRVSPILHRKEPKRQSPQISHLIRPG--SNLYCS-----
Silene_undulata -MFIGFSDKSNCTINPISIILARISPILHRKEPNRFKV-------------------
Camptotheca_acuminata MCVSVAEK--NC---SCNVITSRVSPILHRKEPNRLLYHSTDLNP-RTNYNLFSYIHYSWV
Lonicera_japonica -MFVIVSKSNSCLNTIVS--RILISPIIHRKEPNRHYSILQNPRTTPSNITCLCF------
Swertia_japonica -MFKATDKESSQPSKKPIRAITIISPIIHRKEPNRQPPYSCF------------------
Gevuina_avellana -MFIRIFKNK----GAESNFIARVSPICHRKEPNKSNCLLLLHLL--------RNYLLIW-
Clematis_chinensis -MFINFSATT-----KFNIIKARVSPILHRKEPNRQFCTKLNTL-KPLIQSILC-------
Citrus_trifoliata MCFCIFNFHHLSLVNTECTFHSRVSPILHRKEPNRIIKPQYYY------------------
Nicotiana_tabacum -MFIGAAEN-NHSISNVTTIISRLSPILHRKEPNRNPISFPQNF-V---FSQLGLSR----
Conservation
-973313201-----003003559***8*****6921100000------------------
HG104
POPTR_0010s01710
10
Populus_trichocarpa -MFCFGFGFYPPSVVINRS
Diospyros_lotus MRGCFGLEFTPPRL-INRS
Corchorus_capsularis -MLFFEFGFSPPSL-INRS
Mangifera_indica -MSVFGFGFSPLLVSFNRS
Conservation
-647*484*6*639-8***
HG105
POPTR_0010s09600
10 20 30 40 50 60 70
Populus_trichocarpa MSLVTWRKLWHLSLLLALSLSNQ--P--
Diospyros_lotus MSLRTWRKLWQLSLI--
Macrotyloma_uniflorum MSLRTWRKLWHFSILNTLSLSLSHLKHHKTPHSTTTTTFFFLVFQ--ATKPCYIQVNKQNHINYCAPI-----------
Quercus_kerrii MSLRTWRKLWQIQSSLSLSPKSQ--P--
Catharanthus_roseus MSLRTWRKLWHLCLL--
Pyrus_x_bretschneideri MSLRTWRKLWQFSLPWSYKSSLPRPPPA------PLFPLSFLSPS--LSSPEFLITNRKGKS----------------
Citrus_trifoliata MSLRTWSKLWHLLHSKSKPPIKALVQHQ-TQ---HFVSFSNKVHQKSQVIKFHQQTQKQKKPTFHSRAYIKTQNFVLKEK
Nicotiana_tomentosiformis MSLRTWSKLWHLFLTTISSLL----PPS----SFPLTLFSSLICHKLAFQEKHIVTNPKRDQNL----------------
Conservation
4**668424---

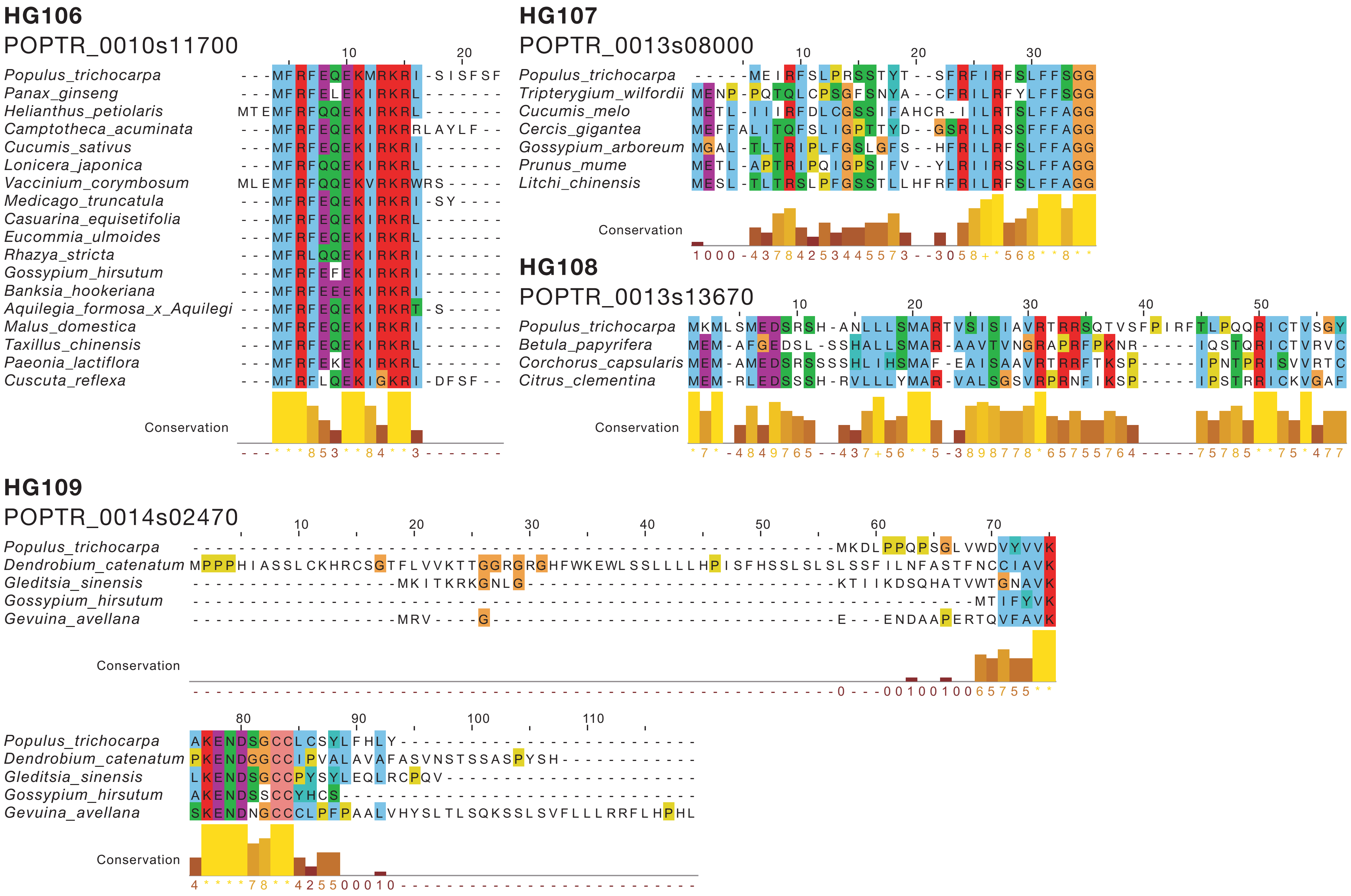
HG106
POPTR_0010s11700
Populus_trichocarpa
Panax_ginseng
Helianthus_petiolaris
Camptotheca_acuminata
Cucumis_sativus
Lonicera_japonica
Vaccinium_corymbosum
Medicago_truncatula
Casuarina_equisetifolia
Eucommia_ulmoides
Rhazya_stricta
Gossypium_hirsutum
Banksia_hookeriana
Aquilegia_formosa_x_Aquilegi
Malus_domestica
Taxillus_chinensis
Paeonia_lactiflora
Cuscuta_reflexa
Conservation
HG107
POPTR_0013s08000
Populus_trichocarpa
Tripterygium_wilfordii
Cucumis_melo
Cercis_gigantea
Gossypium_arboreum
Prunus_mume
Litchi_chinensis
Conservation
HG108
POPTR_0013s13670
Populus_trichocarpa
Betula_papyrifera
Corchorus_capsularis
Citrus_clementina
Conservation
HG109
POPTR_0014s02470
Populus_trichocarpa
Dendrobium_catenatum
Gleditsia_sinensis
Gossypium_hirsutum
Gevuina_avellana
Conservation

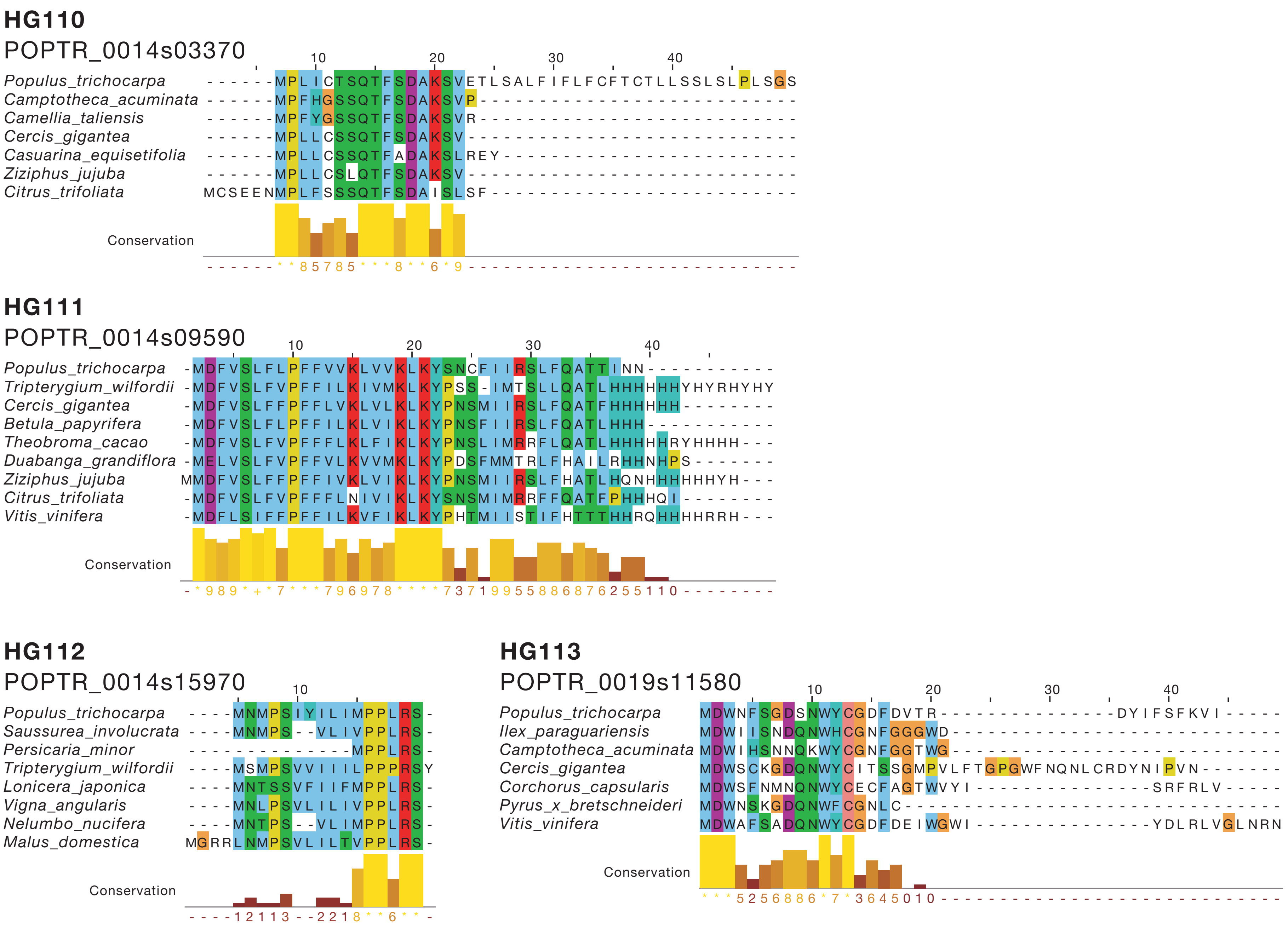
HG110
POPTR_0014s03370
Populus_trichocarpa
Camptotheca_acuminata
Camellia_taliensis
Cercis_gigantea
Casuarina_equisetifolia
Ziziphus_jujuba
Citrus_trifoliata
Conservation
HG111
POPTR_0014s09590
Populus_trichocarpa
Tripterygium_wilfordii
Cercis_gigantea
Betula_papyrifera
Theobroma_cacao
Duabanga_grandiflora
Ziziphus_jujuba
Citrus_trifoliata
Vitis_vinifera
Conservation
HG112
POPTR_0014s15970
Populus_trichocarpa
Saussurea_involucrata
Persicaria_minor
Tripterygium_wilfordii
Lonicera_japonica
Vigna_angularis
Nelumbo_nucifera
Malus_domestica
Conservation
HG113
POPTR_0019s11580
Populus_trichocarpa
Ilex_paraguariensis
Camptotheca_acuminata
Cercis_gigantea
Corchorus_capsularis
Pyrus_x_bretschneideri
Vitis_vinifera
Conservation

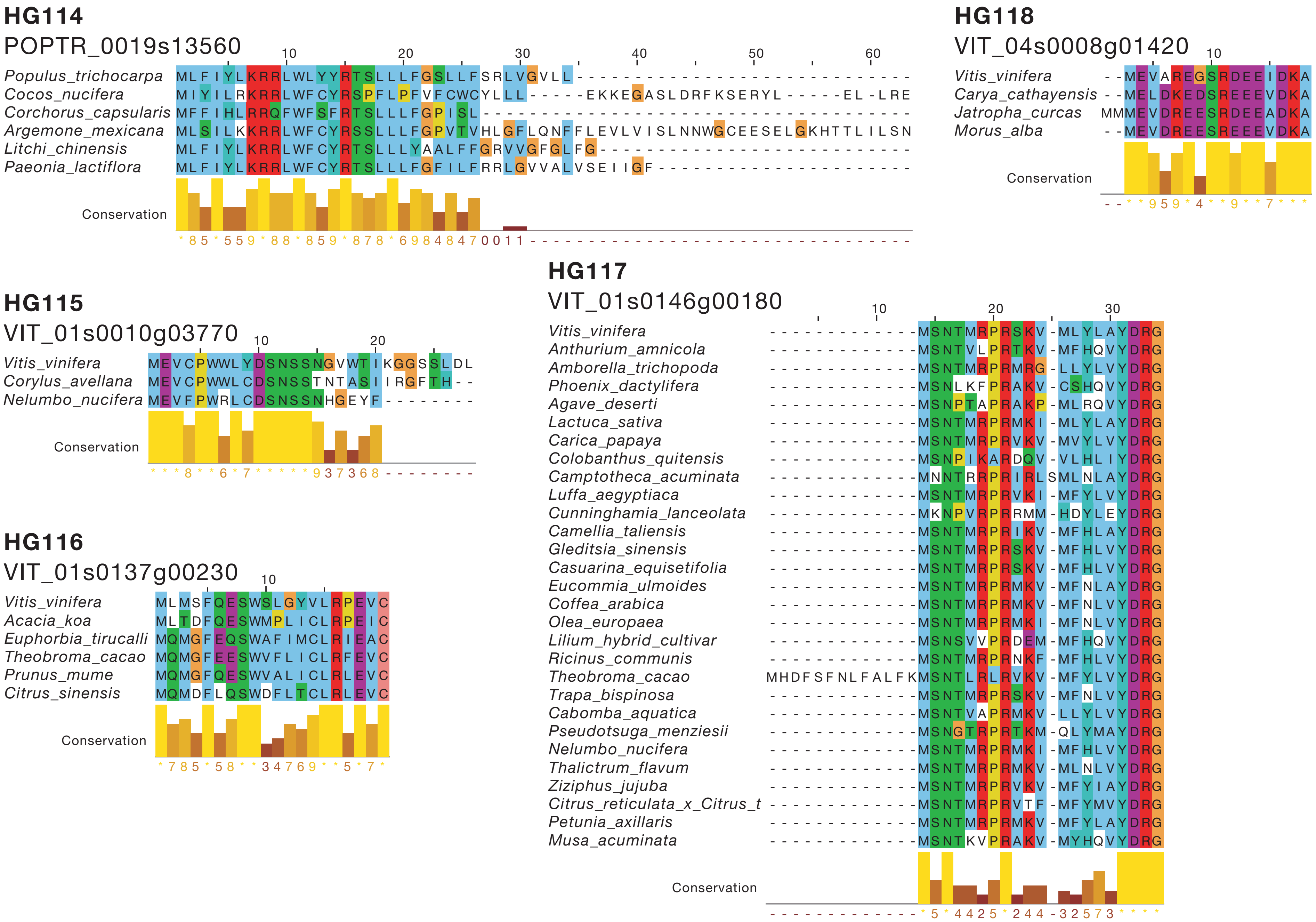
HG114
POPTR_0019s13560
Populus_trichocarpa
Cocos_nucifera
Corchorus_capsularis
Argemone_mexicana
Litchi_chinensis
Paeonia_lactiflora
Conservation
HG118
VIT_04s0008g01420
Vitis_vinifera
Carya_cathayensis
Jatropha_curcas
Morus_alba
Conservation
HG115
VIT_01s0010g03770
Vitis_vinifera
Corylus_avellana
Nelumbo_nucifera
Conservation
HG117
VIT_01s0146g00180
Vitis_vinifera
Anthurium_amnicola
Amborella_trichopoda
Phoenix_dactylifera
Agave_deserti
Lactuca_sativa
Carica_papaya
Colobanthus_quitensis
Camptotheca_acuminata
Luffa_aegyptiaca
Cunninghamia_lanceolata
Camellia_taliensis
Gleditsia_sinensis
Casuarina_equisetifolia
Eucommia_ulmoides
Coffea_arabica
Olea_europaea
Lilium_hybrid_cultivar
Ricinus_communis
Theobroma_cacao
Trapa_bispinosa
Cabomba_aquatica
Pseudotsuga_menziesii
Nelumbo_nucifera
Thalictrum_flavum
Ziziphus_jujuba
Citrus_reticulata_x_Citrus_t
Petunia_axillaris
Musa_acuminata
Conservation
HG116
VIT_01s0137g00230
Vitis_vinifera
Acacia_koa
Euphorbia_tirucalli
Theobroma_cacao
Prunus_mume
Citrus_sinensis
Conservation

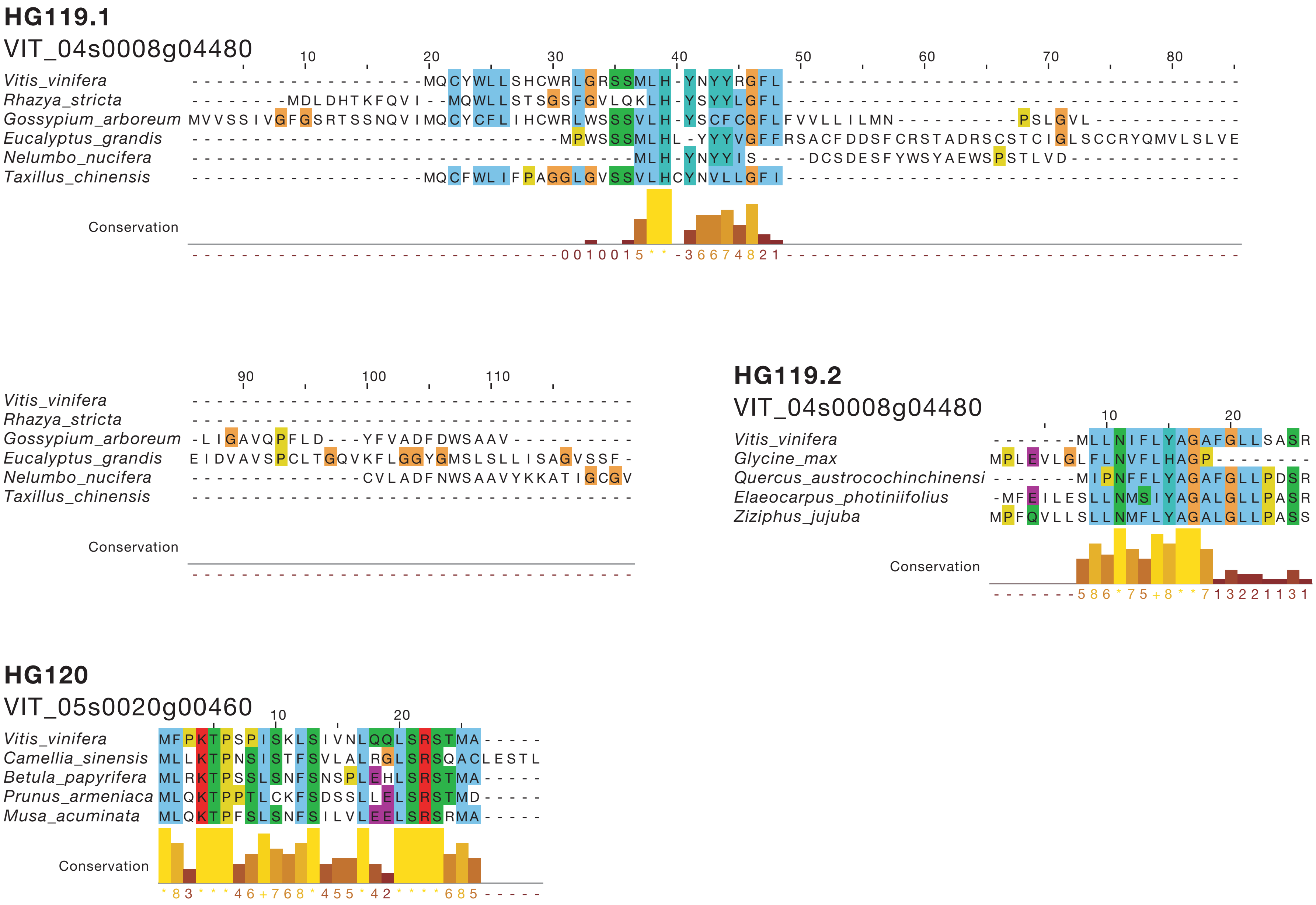
HG119.1
VIT_04s0008g04480
Vitis_vinifera
Rhazya_stricta
Gossypium_arboreum
Eucalyptus_grandis
Nelumbo_nucifera
Taxillus_chinensis
Conservation
HG119.2
VIT_04s0008g04480
Vitis_vinifera
Glycine_max
Quercus_austrocochinchinensi
Elaeocarpus_photiniifolius
Ziziphus_jujuba
Conservation
HG120
VIT_05s0020g00460
Vitis_vinifera
Camellia_sinensis
Betula_papyrifera
Prunus_armeniaca
Musa_acuminata
Conservation

HG121

VIT_05s0029g00470

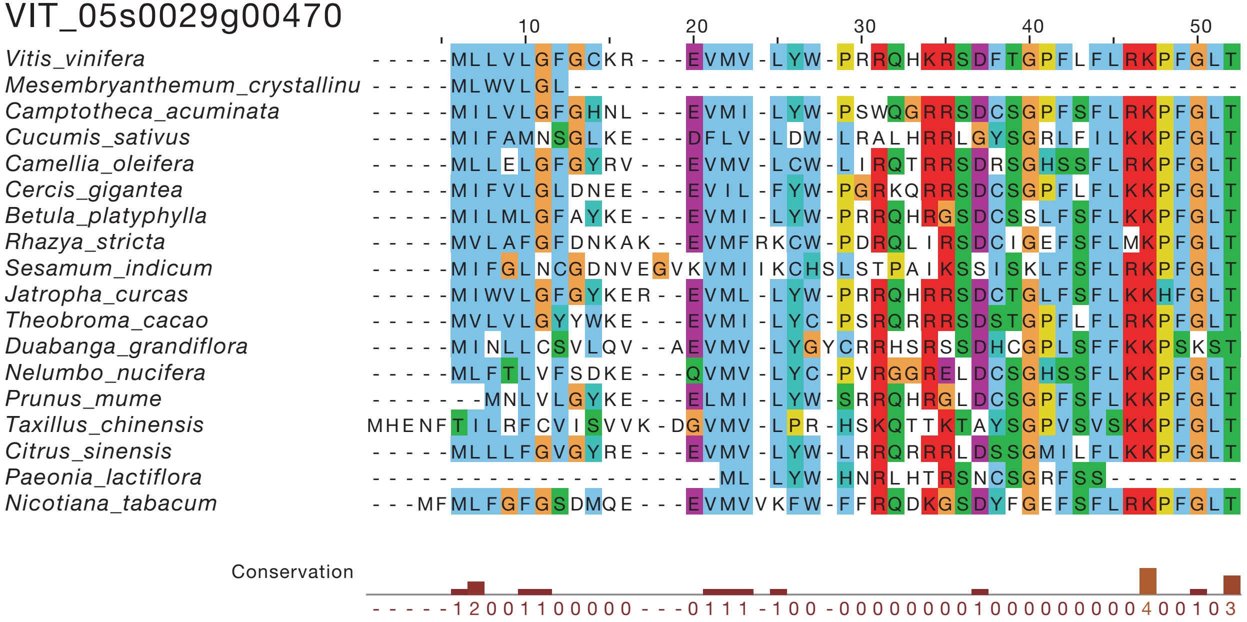

HG122

VIT_06s0004g02980

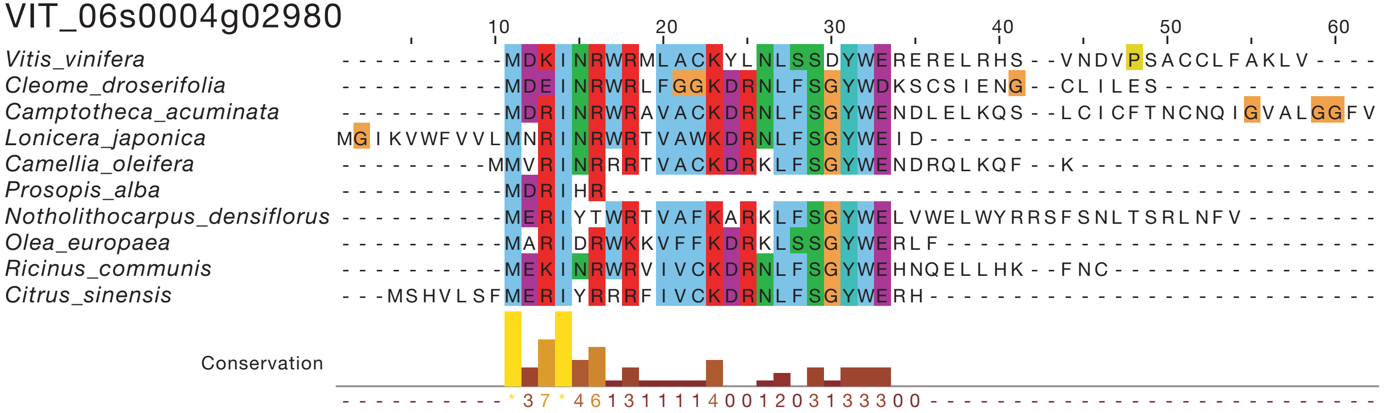

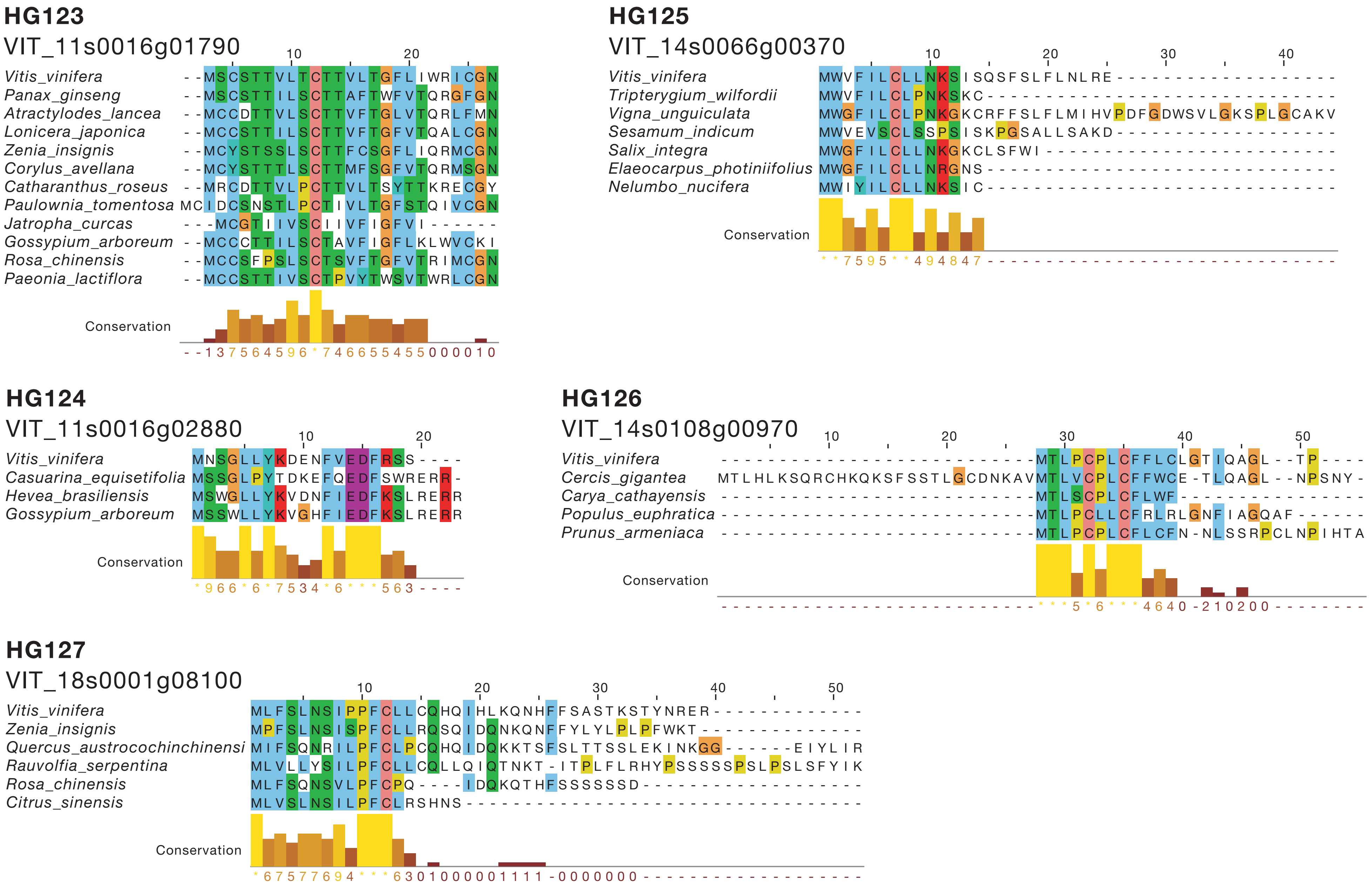

HG123
VIT_11s0016g01790
Vitis_vinifera
Panax_ginseng
Atractylodes_lancea
Lonicera_japonica
Zenia_insignis
Corylus_avellana
Catharanthus_roseus
Paulownia_tomentosa
Jatropha_curcas
Gossypium_arboreum
Rosa_chinensis
Paeonia_lactiflora
Conservation
HG125
VIT_14s0066g00370
Vitis_vinifera
Tripterygium_wilfordii
Vigna_unguiculata
Sesamum_indicum
Salix_integra
Elaeocarpus_photiniifolius
Nelumbo_nucifera
Conservation
HG124
VIT_11s0016g02880
Vitis_vinifera
Casuarina_equisetifolia
Hevea_brasiliensis
Gossypium_arboreum
Conservation
HG126
VIT_14s0108g00970
Vitis_vinifera
Cercis_gigantea
Carya_cathayensis
Populus_euphratica
Prunus_armeniaca
Conservation
HG127
VIT_18s0001g08100
Vitis_vinifera
Zenia_insignis
Quercus_austrocochinchinensi
Rauvolfia_serpentina
Rosa_chinensis
Citrus_sinensis
Conservation

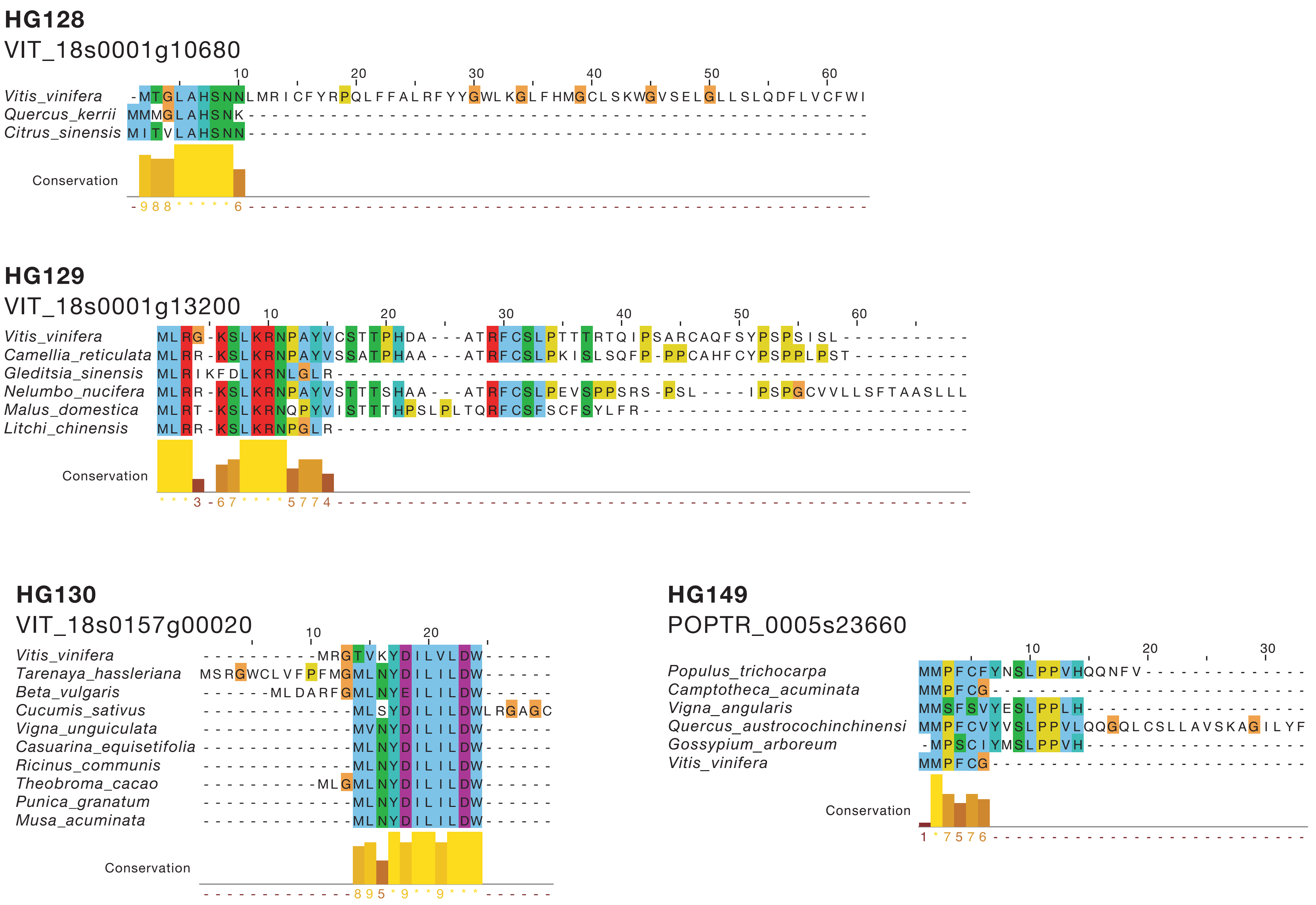
HG128
VIT_18s0001g10680
Vitis_vinifera
Quercus_kerrii
Citrus_sinensis
Conservation
HG129
VIT_18s0001g13200
Vitis_vinifera
Camellia_reticulata
Gleditsia_sinensis
Nelumbo_nucifera
Malus_domestica
Litchi_chinensis
Conservation
HG130
VIT_18s0157g00020
Vitis_vinifera
Tarenaya_hassleriana
Beta_vulgaris
Cucumis_sativus
Vigna_unguiculata
Casuarina_equisetifolia
Ricinus_communis
Theobroma_cacao
Punica_granatum
Musa_acuminata
Conservation
HG149
POPTR_0005s23660
Populus_trichocarpa
Camptotheca_acuminata
Vigna_angularis
Quercus_austrocochinchinensi
Gossypium_arboreum
Vitis_vinifera
Conservation

HG150

POPTR_0007s12170

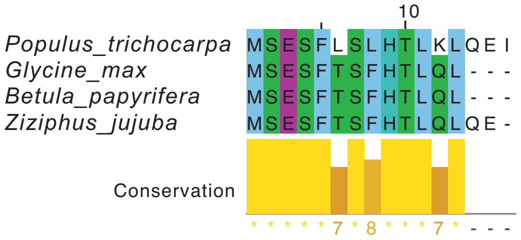

HG151

VIT_01s0137g00280

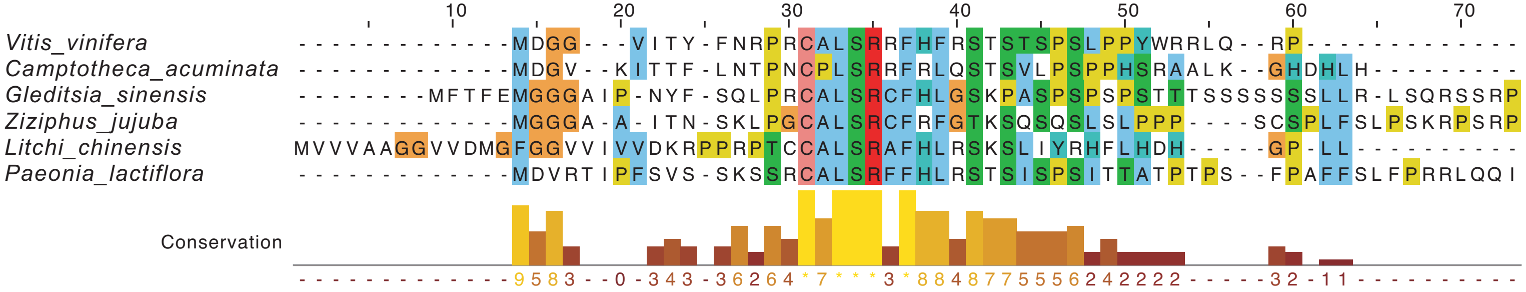

Supplementary Figure S1. Alignments of the newly identified CPuORF sequences. The amino acid sequences of the novel CPuORFs and their homologous putative uORFs were aligned using ClustalO ver. 1.2.2 and displayed using Jalview ver. 2.10.2. For each alignment, a putative uORF sequence was selected from each order in which uORF-tBLASTn and mORF-tBLASTn hits were found. In the alignments of the LOC_Os11g16280 (HG075), POPTR_0009s15460 (HG103), and POPTR_0019s11580 (HG113) CPuORFs, some putative uORF sequences were manually removed.
