# Supplementary material for: Comprehensive genome-wide identification of angiosperm upstream ORFs with peptide sequences conserved in various taxonomic ranges using a novel pipeline, ESUCA
Source: BMC Genomics. 2020 Mar 30;21:260. doi: 10.1186/s12864-020-6662-5 (PMC7106846; doi:10.1186/s12864-020-6662-5)
Supplement: Supplementary file 8 — Additional file 8 : Supplementary Figure S2. 5 ´-UTR nucleotide and deduced amino acid sequences of the poplar CPuORFs analyzed in the transient expression study. [file 12864_2020_6662_MOESM8_ESM.pdf]

Supplementary Figure S2

A HG46

```
  1 AGAAGCCAAAAAAAAAAGAAAAGATACAGGACAGTAGAACGTTGGAGAAACCTCCCTGCC

 61 TCCTTCCTTAATCTTTCCTTCATAGGTGAACAGAACAGAACAGGGTTGCCGATACTTTTA

121 TGCGGTTTTGCTAAATTCACTAGCCTGTTCTCCTGTGACCCAAGAAAGAATCATTTACTG

181 GTAGGTTTTCTCCAACAATTAATAAACAGAAGTGTAAAAACTGTGTTAATGTTGATTGAT

241 CTCAATTGGGTTTTGTTTATTTGGTCATTGGGTTAGCATTATAAAAGGGTTATGAGAATT

301 CTGTACTGTTTGATTGAAAAGTTTTGATTTTGAGACAAGGGTTTGGATTTTTTGGTCTGG

361 GATTGATTGGATTGTTGTTCTTGCTGGTTTGGCTGAGGTTATTGGATCTGGTTTTTTATT

421 TTGGATTGGTAGTGTTTGATAGAAGTGGTTATCTTGGAATGATTGTTGGTGGGTTTGTGA

481 TAGATCCATGGGAGAATTGTCGTGATTGAAGGGATTTGATTACTTGGTCTGGTGTGACTA

541 GTTTCTTTGTGTTTGTTGATGAATATTGTTATTTTTGAAGAGGAGGATCGGCTTGTGAAC
                      M  N  I  V  I  F  E  E  E  D  R  L  V  N
                      M  N  I  V  I  F  E  E  E  D  R  L  V  N

601 TTTCCCAATTCGCTTCTGGGTGTTCTCAGGCCCTTGGATTTACTTGTTGAACGAAAGGTC
    F  P  N  S  L  L  G  V  L  R  P  L  D  L  L  V  E  R  K  V
    F  P   I  R  F  W  V  F  S  G  P  W  I  Y  L  L  N  E  R  S
                                                          G
661 TTTTCTTTTTGTATCCCTAGAGATAGATGGGATAAGGAGTTTTTCAAGCTTCAGAGTGGA
    F  S  F  C  I  P  R  D  R  W  D  K  E  F  F  K  L  Q  S  G
     F  L  F  V  S  L  E  I  D  G  I  R  S  F  S  S  F  R  VG

721 TAATTACTATTATCACCATCATCCATCTAATTTTCAAAGGTTGTGATTGTATAATTTTGG
    *
    *

781 TTTGGAAATTGGAATTTTGGGAAATGAG
```

B HG55

```
  1 CTCTTTCTATGTACTATACCTCTCACCTTACCATTTCCACATCTCTTCTACATCATCATC

 61 GTCATCATTACACCACCTTTTCGCTCACTTGACATCACTTTCCGGCAACAAGTCCGATCT

121 CTTTTCCTTCTCTTTAGACAGGATTATTAAATTACTTCCTGGCAAGTGAAGTCTTTTTTT

181 GCTTACTTTTCTTTCTATGATTGATTCAATTGAGGAAATTCTAGAGCTGGAAGTCGGCTA

241 AGGGTTTTTGGTTTTTTGTTAAGTGGGTTTTGGAGGAATAGGTAGTTTGATTAAAGGGTA

301 ATCTGGTATGTGGGTTGCGGTTAACTGGAATCATGGGTGTGTGGGTGTGGTTTTTTTGTA
           M  W  V  A  V  N  W  N  H  G  C  V  G  V  V  F  L  Y
           M  W  V  A  V  N  W  N  H  G  C  V  G   W  F  F  C  T
                                    C
361 CTTTTGGGGAAGAAGCGACGCTGATTAGGTGTGGTTTTCATTTAAATCTTTCGTCTTTTT
     F  W  G  R  S  D  A  D  *
      F  G  E  E  A  T  A  D  *

421 TGGTTTAGTTTATCTGGGGTTTTGCTGTTTTTCTTTTTAGTTCTCAGTGTTGGTTCAGTT

481 TGTGAAATTTTGGATCTTTTGGGTACTCGTTATGGA
```

C HG57

```
  1 AACAGATACAACACAATCTCCATACTGAAAACATTGTTTTTCAGATGAAATACTACCGGG

 61 TTTTAGACAGAATATCGCGAAAGTTCTGAGATGCATCTAATGTTTGGAGTTCCTGTTGTT
                                  M  H  L  M  F  G  V  P  V  V
                                  M  H  L  M  F  G  V  P   L  F
                                              C
121 CAAGGAGATCATAAAGTTGAAGAGAGAAAGTGGGAGAAAGAGAGATAGATATTAGCTCAG
    Q  G  D  H  K  V  E  E  R  K  W  E  K  E  R  *
     K  E  I  I  K  L  K  R  E  S  G  R  K  SR  *

181 GCCAAGAAAACTTCAGATTTAGTATTAATTCTCTTTAGTGCGTAAAGGGTTTTTTCTTTT

241 TTCTATTTTTTTCTATTTTTTGCATTCAGACTCTTTTATTGCCCATAAAGCTGAAAGCAT

301 TGAAGGATTTTTAAGAGTGATTTAAGGGAAACGAATGGC
```

D HG65

```
  1 GACAACCCTCTCCAAACTCCAAAACAACAAGTCTCTCAAGAGTTTCCAAATTAGGGTTTG

 61 TTCTTATTTTAATGGAAACCTCCTCCGTTAACACATTAAGAGTTCGTTACTCCCGTTGCA
               M  E  T  S  S  V  N  T  L  R  V  R  Y  S  R  C  N
               M  E  T  S  S  V  N  T  L  R     F  V  T  P  V  A
                                             T
121 ATTGCTTTAAACGTTGTTGTCACTGTTTCTGTTGTTTCTACTCTTACCCCTGAAAACTTA
      C  F  K  R  C  C  H  C  F  C  C  F  Y  S  Y  P  *
    I  A  L  N  V  V  V  T  V  S  V  V  S  T  S Y  P  *

181 TAACCAAAAATCCCTGAAAAAAAAGCCCCAATTTTTATTTTTATTAGAGATGGA
```

E HG66

```
  1 AGCCCAGAATGTCCATCTCCTATAATTCGCTTCTCAATTACCCCTTGATTTCCTTTTACT

 61 CACGGTCTTACATTACAGGTTATGATAGTAAACAGCTACCGGAAACCTGAGAGCCAACGC
                         M  I  V  N  S  Y  R  K  P  E  S  Q  R
                         M  I  V  N  S  Y  R  K  P  E  S  Q  R
                                                            C
121 TCCTCTCCTGCTCTCGGTTTCCCTCGCCCGCGTTTCCTTCGCTACCGCCGTTTGAGGTAA
    S  S  P  A  L  G  F  P  R  P  R  F  L  R  Y  R  R  L  R  *
    S  S   L  L  S  V  S  L  A  R  V  S  F  A  T  A  VL  R  *

181 CCGGTGAAATAGAGTAACTGGAGGGAAAGAGATTTTTTATTGTATTTTTTTATTTTTTCG

241 TGTAACACCGTTACTGTCGCTTACAATTATGCC
```

F HG80

```
  1 GAGATTATAATCAAGGTGGTCAATTGATCATATTTTGTACTTGCTAATAGTATAATTAAC

 61 TTGTTGTGTTGGTTCTGAATGATGGTGTAGTAGGTATTTCTTCCTCTGAAATGCATTTGT

121 GAGTTGCAGCAGTTCACAAAAACAATTTTTTGTTGACAATGTTTCTGCAAGATGGGCATA
                                          M  F  L  Q  D  G  H  I
                                          M  F  L  Q  D  G  H  I
           C
181 TTGGGTTTTCGGATACAAGCGATCGCAATCCTTCCTGGAAGTTCCTCTTTTTGTTTTTCA
      G  F  S  D  T  S  D  R  N  P  S  W  K  F  L  F  L  F  F  I
      G  F F  G  Y  K  R  S  Q  S  F  L  E  V  P  L  F  V  F  H

241 TCTTTACAGTTCTTTTGTGTTTCGGATCGTGATAAAAGTTTGCAAATTCAGATTTACTAG
      F  T  V  L  L  C  F  G  S  *
     L  Y  S  S  F  V  F  R   S  *

301 GAGTTGTTGGATACAAATTTTCAATGCA
```

G HG81

```
  1 AAATGTCCTCTTCATTGATTGAGACCCCATTAAAAAAGAAAAGAGAAAAGAGAAAAACAA

 61 AGAAAGAGGTAAAAGCAGAAGCATCAGCAGCAAGCAACACAACCACGTACTATAATATCC

121 ATCGTTACTGGGAAAAATAAACTCTCCCCATAAATCTTCCGTTTCTTCTCCTCTGTTAAC

181 ATGTTGTGAATATAGGTAGCATACAAGTGCTTGAAGCAGTGTGGTGTGGTAGCTATGAAA

241 ATGTTGCGCACTCGGTTTTTAGTCCATGGTTAATGCTATTTGGGGTGTGAAAAGATCTGG

301 GTTTTCTCAGAGAACTCAAAATTTAGTGACATTCTACTAAGATCTAGCTTAAATGAGCCG

361 CACAAGTGAGTGCTTTTGTCGGCTGGTTAAAGCTTATTTGAAAAGCGGATGAGGCTGAAA
                                                    M  R  L  K
                                                    M  R  L  K
                                            T
421 TCGATCGTGTTTTGTAGCAAAACCGGTTTCGGTTGAGGATTTAGAAACTGGGTCCGTTTC
    S  I  V  F  C  S  K  T  G  F  G  *
     R  S  C  F  V  A  K  P  V  SG  *

481 TTTGCTTTTCTTGGCTTCCAGATGAACTTGTCTTTGATGGTTTAATAGTTTAGTCATTCT

541 TTTTTGGGTAAGAGGGAAGGTAAAAAGGTGAAGAACAGGAAGAAGAAGGCATGATAGTGT

601 CATTGTGTGAAGGGAAGAAATTAGTGAAGATTGTTTGGGTTGTGGGTCTAAACTTGTTGC

661 GATTGTATTATTAAATCTCTCCCCCTCTCTCAGTCTTAATGAG
```

H HG87

```
  1 AAGGAAAGGGTGCTGAGTATATCAATCAAGAATTTCTGAGACCACAAAGAAGCTAGGTAG

 61 CTTGAGTAAGCTTGATCTTCTCAAGTTCTTGTTATCGTCATTAATTTCCCTGTAAGAGAT

121 AGTTTAATATAGACTGGTATTTAGGGAGGAGACTATACTAATAAGATAGATAGTGACTCG

181 AATAGACATGCTCACTTCTCATCACCTTCCTACCCATTTCCTAGCAACATTTCACTCAAT
           M  L  T  S  H  H  L  P  T  H  F  L  A  T  F  H  S  I
           M  L  T  S  H  H  L  P  T  H  F  L  A  T  F  H  S  I
                           C
241 TATATCACCGATTTCACTTGAGCAATATTTCACTGTTCGCCTCACAAGGATCAACCTCAT
     I  S  P  I  S  L  E  Q  Y  F  T  V  R  L  T  R  I  N  L  I
     I  S  P  I  S  L  E  H I  F  H  C  S  P  H  K  D  Q  P  H

301 CCTCTTGTTATTCTTCATATTGATTTCTCTTCTTGCTCCAAGGTCCAAGGGCTAGCCAGT
     L  L  L  F  F  I  L  I  S  L  L  A  P  R  S  K  G  *
    P  L  V  I  L  H  I  D  F  S  S  C  S  K  V  Q   G  *

361 TAGAATTTGTTTGCTTTGTTCCTGTCTGAAAATAGAGAAAATTTGCTTCCTCTCACTCTC

421 TTGGTTGTTATTTAGTTGCTAATCTTGTAGTGTTTTAAGAGATGGG
```

I HG88

```
  1 TGATTAAACAATTTCGAAGACTTTCCTTTCTCTTCTCTGGTGCTATAAGCTATTTGAGAC

 61 TGGGCTTGATTAATGGCAATGCATAATATATTGCTGTCCTTTTGGTTCGCATAGGACTGGA
                 M  A  M  H  I  Y  C  C  P  F  G  S  H  R  T  G
                 M  A  M  H   Y  I  A  V  L  L  V  R  I  G  L  E
                                         C
121 ATTTGGAATTGTTGCAGAGAGAGAAACTTGCTTTTGGATGCTTACTTGTAAACTAGAGCA
    I  W  N  C  C  R  E  R  N  L  L  L  D  A  Y  L  *
     F  G  I  V  A  E  R  DN  L  L  L  D  A  Y  L  *

181 GCCCAAGTTGGTTTTTTTTTTTTTAATTTTATTTGGATGCTCAATTTTTAATACAAGTTCG

241 GTGATTGATAAGTGGTTCTTCGAGTTAGTTTCAGTGATTTCTGTTATTAGATTTGGGTTG

301 GTGGGTTAGCTGCTACATTCTTTGCATCATGCC
```

J HG103

```
  1 ACTGGTATCTCTCTCTCCCTTTTCTAATTATGCTTTCAAGAACACAGTCATATAGAGAAA
                                 M  L  S  R  T  Q  S  Y  R  E  I
                                 M  L  S  R  T  Q  S  Y  R  E  I

 61 TAACAAGAACAACAACATCAAGAAAATCCAGGGTTTCTCCGATCCTCCATAGAAAAGAAC
      T  R  T  T  T  S  R  K  S  R  V  S  P  I  L  H  R  K  E  P
      T  R  T  T  T   Q  E  N  P  G  F  L  R  S  S  I  E  K  N
             C
121 CTAACAGACATAAAAAATCTCTTCTTGTTATGCACCCCTTTTGCCTTATTTAGTGGGCCT
      N  R  H  K  K  S  L  L  V  M  H  P  F  C  L  I  *
    L  T  D  H K  K  S  L  L  V  M  H  P  F  C  L  I  *

181 ATACTTTCTCTTACACAAATTCCTAACTTGGGTTTTTCTTGTTTTGTCTAATTCAGTGGT

241 TCTTGTTTGTTTTTGTTGTTGTCGTTGTTGTTAAATTGTTGTTGAAGTTTGTTTTAGGGT

301 TTTAAGATGGG
```

K HG107

1 GAGTTTCCGCGTCAAAAATCAGTGAGCCAGCAGAACACACCACTCGTCCTTCAATTTCAT

61 TCTCTGCCGCCGATGCCGCTGCTGCTCGTTTGTTTTCGTTTTTAAAAAAACCAAACAAAA

121 ACCAATCCATCCATCTATCTGCCTTTATATCATATTAGATAAGAAAACAAGGGAATTTCG

A

181 AAAACAGATCGGTGAAGGGAAGGAAAAAAAACCAAACCCTAAAAGGGAAGAAAATGAAAA

241 GTCAGCAATTCTGA**ATGGAAATCCGATTCTCTTTACCCAGATCATCCACTTACACCTCTT**

M E I R F S L P R S S T Y T S F

M E I R F S L P R S S T Y T L

C

301 **TCCGGTTCATCCGCTTCTCTCTCTTCTTCTCTGGAGGCTGATTGATTTTCTTTTTCTTTT**

R F I R F S L F F S G G *

S G S S A S L S S S L E A *

361 TTGTTTCCTGTAAAATAAAAACTGCATTTACATATATATATATATATATAGAGAGAGAGA

421 GAGAGAGCGCGCGCGCTCGTGGATGGA

Supplementary Figure S2. 5´-UTR nucleotide and deduced amino acid sequences of the poplar CPuORFs analyzed in the transient expression study. (A-J) The 5´-UTR nucleotide sequences are based on the sequences of the *Populus nigra* full-length cDNA clones, pds25559 (A), pds10965 (B), pds14390 (C), pds12940 (D), pds13862 (E), pds15817 (F), pds28294 (G), pds26157 (H), pds14623 (I) and pds23234 (J) (GenBank accession no. DB890234, DB875826, DB879210, DB877779, DB878687, DB880616, DB892931, DB890822, DB879437 and DB887941, respectively). We determined the pds25559 and pds28294 5´-UTR nucleotide sequences by sequencing because sequence information of these regions was not available in public databases. Arrows indicate the positions of primers used for the 5´-UTR cloning. (K) The 5´-UTR nucleotide sequence of the *Poplus trichocarpa* POPTR_0013s08000 gene is based on NCBI Refseq XM_002319213.3. The nucleotide 'A' shown in a black background indicates the nucleotide change from T to A for the removal of the start codon of the uORF located immediately upstream of the HG107 CPuORF. In (A)-(K), the CPuORF nucleotide and deduced amino acid sequences are shown in bold. The nucleotide sequences of uORFs other than the CPuORFs are underlined. The initiation codon of the mORF in each sequence is boxed. The nucleotides that were deleted and inserted in the frameshift mutants are shaded, and the deduced amino sequences of the frameshift mutant CPuORFs are indicated.
